# Novel Antineoplastic Inducers of Mitochondrial Apoptosis in Human Cancer Cells

**DOI:** 10.3390/molecules29040914

**Published:** 2024-02-19

**Authors:** Andreas J. Kesel

**Affiliations:** Independent Researcher, Chammünsterstr. 47, D-81827 München, Bavaria, Germany; andreasj.kesel@gmx.de; Tel.: +49-(0)176-87974849

**Keywords:** cancer growth, tumor metastasis, cancer’s *Warburg* effect, p53, p53 (re)activators, mitochondrial apoptosis, cytochrome *c* release, tumoricidal drug effect

## Abstract

I propose a new strategy to suppress human cancer completely with two entirely new drug compounds exploiting cancer’s *Warburg* effect characterized by a defective mitochondrial aerobic respiration, substituted by cytosolic aerobic fermentation/glycolysis of D-(+)-glucose into L-(+)-lactic acid. The two essentially new drugs, compound **1** [**P(op)T(est)162**] and compound **3** (**PT167**), represent new highly symmetric, four-bladed propeller-shaped polyammonium cations. The in vitro antineoplastic highly efficacious drug compound **3** represents a covalent combination of compound **1** and compound **2** (**PT166**). The intermediate drug compound **2** is an entirely new colchic(in)oid derivative synthesized from colchicine. Compound **2**’s structure was determined using X-ray crystallography. Compound **1** and compound **3** were active in vitro versus 60 human cancer cell lines of the National Cancer Institute (NCI) Developmental Therapeutics Program (DTP) 60-cancer cell testing. Compound **1** and compound **3** not only stop the growth of cancer cells to ±0% (cancerostatic effect) but completely kill nearly all 60 cancer cells to a level of almost −100% (tumoricidal effect). Compound **1** and compound **3** induce mitochondrial apoptosis (under cytochrome *c* release) in all cancer cells tested by (re)activating (in most cancers impaired) p53 function, which results in a decrease in cancer’s dysregulated cyclin D1 and an induction of the cell cycle-halting cyclin-dependent kinase inhibitor p21^Waf1^/p21^Cip1^.

## 1. Introduction

Adenosine 5′-triphosphate (ATP) is required for normal cell proliferation and survival and comes primarily from two sources. The first is glycolysis, which comprises a series of reactions that metabolizes D-(+)-glucose to pyruvate in the cytoplasm to produce a net of 2 molecules ATP from each D-(+)-glucose. The other is the citric acid cycle (CAC), also known as the *Krebs* cycle, *Szent Györgyi*−*Krebs* cycle or the tricarboxylic acid cycle (TCA cycle), which uses pyruvate formed from glycolysis in a series of reactions that donate electrons via nicotinamide adenine dinucleotide (NADH/H^+^) and flavin adenine dinucleotide (FADH_2_) to the respiratory chain complexes in the mitochondria. With oxygen (O_2_) serving as the final electron acceptor, electron transfer across the mitochondrial inner membrane creates a proton gradient, which forms in summary about 30–32 molecules ATP per one D-(+)-glucose molecule by catalysis of F_1_F_O_–ATP synthase. In conditions of oxygen limitation, such as within muscles under prolonged exercise, pyruvate is not utilized in the citric acid cycle (CAC) but is converted into L-(+)-lactic acid (‘Fleischmilchsäure’) by lactate dehydrogenase (LDH) in a process termed anaerobic glycolysis.

In 1930, *Otto Heinrich Warburg* (1883–1970) published [1] his theory on the origin of cancer cells based on a series of preceding investigations [2,3,4]. He summarized his theory on the origin of cancer cells in 1956 [5]. This theory was coined the *Warburg* hypothesis [6]. The essence of this hypothesis on the origin of cancer is that, under aerobic conditions, malignant tissues metabolize approximately tenfold more glucose to lactate in a constant time window than primary tissues, a phenomenon known as the *Warburg* ‘malignant’ effect. *Warburg* claimed that cancer cells heavily rely on aerobic glycolysis as an energy source for malignant growth rather than aerobic respiration, thereby claiming a defect in cancer cell respiration. The *Warburg* hypothesis has been heavily debated for many years since its introduction [6], especially regarding the respiratory defect misinterpreted by *Warburg* himself [6], and only in recent years many details of the *Warburg* hypothesis were confirmed as correct [7,8,9,10,11,12]. Importantly, the *Warburg* effect itself, which was proved to constitute a fact in vivo [13], has to be differentiated from the complete *Warburg* hypothesis, which was the cause of ongoing scientific debate [14,15,16].

Many cancer cells consume D-(+)-glucose heavily and produce L-(+)-lactic acid rather than catabolizing D-(+)-glucose via the citric acid cycle (CAC), which is normal for generating ATP in non-hypoxic healthy cells. The avid uptake of D-(+)-glucose by tumors is the prerequisite for the detection and monitoring of human tumors by 2-deoxy-2-[^18^F]fluoroglucose positron emission tomography (PET). More than 90 years ago, *Otto Heinrich Warburg* observed that thin slices of human and animal tumors ex vivo displayed high levels of D-(+)-glucose uptake and L-(+)-lactate production. The shift toward L-(+)-lactate production in cancers, even in the presence of adequate oxygen, is termed the *Warburg* effect or aerobic glycolysis [1,2,3,4,5,6,7,8]. These observations have been confirmed, although the nuances of aerobic glycolysis and its molecular characteristics are still under investigation. Tumors display aerobic glycolysis partly through activation of proto-oncogenes or loss of tumor suppressors, which is then further intensified by stabilization of the evolutionary conserved hypoxia-inducible factor (HIF) via adaptive response to hypoxic microenvironment or via pathways that stabilize HIF under non-hypoxic conditions.

In 2002, it was shown that human cancer cells indeed suffer from defects in cellular respiration, either a marked depletion in cellular mitochondrial content or a selective repression in expression of the catalytic β-subunit of mitochondrial Complex V F-type ATPase (β-F_1_-ATPase) concurrent with an increase in the expression of the glycolytic enzyme glyceraldehyde 3-phosphate dehydrogenase [7]. Both mechanisms impair mitochondrial respiration and give support to the *Warburg* hypothesis [7]. In rat rhabdomyosarcoma, R1H cells’ mitochondrial function was found to be deficient by a dysregulation of the mitochondrial protein-to-cardiolipin ratio [10]. Mitochondrial respiration of R1H cells was significantly impaired, joined by the incapacity of the rhabdomyosarcoma cells to differentiate into mature striated skeletal muscle cells [10]. The intriguing abnormalities in cardiolipin content were confirmed in vivo utilizing brain tumors grown in mice [9]. The compositional cardiolipin abnormalities involved an abundance of immature molecular species and deficiencies of mature molecular species, suggesting major defects in cardiolipin synthesis and remodeling in rodent brain tumor tissue [9]. The tumor cardiolipin abnormalities were also associated with significant reductions in both individual and linked electron transport chain activities [9]. The acidic phospholipid cardiolipin [1,3-bis(*sn*-3′-phosphatidyl)-*sn*-glycerol, 1,3-diphosphatidyl-*sn*-glycerol] is known to anchor cytochrome *c* together with cytochrome c oxidase at the inner mitochondrial membrane [17,18,19,20]. Abnormalities in cardiolipin content and composition thus strongly impair cellular mitochondrial respiration by impairment of cytochrome *c*/cytochrome *c* oxidase function(s) of Complexes III (coenzyme Q—cytochrome *c* reductase) and IV (cytochrome *c* oxidase) of the electron transport chain [9]. It was reported in 2015 that the acyl fatty acid composition of cardiolipin correlates with prostate cancer PC-3 cell proliferation [21]. Cardiolipin was reviewed in 2020 to play a generally important role in cancer [22]. Integrated lipidomics and proteomics revealed in 2021 acyl fatty acid alterations in pancreatic cancer stem cell cardiolipin [23], an upregulation of hydroxyacyl-coenzyme A (CoA) dehydrogenase/3-ketoacyl-CoA thiolase/enoyl-CoA hydratase (trifunctional protein HADHA, the α-subunit of mitochondrial long chain fatty acid β-oxidation trifunctional enzyme) in pancreatic cancer stem cells [23], and upregulation of long chain fatty acid elongation enzymes in pancreatic cancer stem cells [23]. The data indicated a crucial role of fatty acid elongation and alteration in cardiolipin acyl chain composition in pancreatic cancer stem cells, suggesting these characteristics as attractive therapeutic targets in pancreatic ductal adenocarcinomas [23]. In 2023, it was reported that mass spectrometry imaging revealed abnormalities in cardiolipin composition and distribution in banked human astrocytoma/glioblastoma brain tumor tissues in comparison to healthy control brain cortex tissue [24]. Hence, there is accumulating growing evidence that cardiolipin abnormalities represent a characteristic trait of cancer as its signature phospholipid both in vitro and in vivo.

The intrinsic pathway of programmed cell death (apoptosis [25,26,27,28,29,30,31]) triggers cytochrome *c* release from the inner mitochondrial membrane into the cytosol [30], where cytochrome *c* associates with apoptotic protease activating factor 1 (Apaf-1, apoptotic peptidase activating factor 1) and procaspase 9 to form the apoptosome [30]. The apoptosome, in turn, activates the effector caspases 3, 6, and 7 to execute programmed cell death [30]. In cancer cells this mechanism is defective or inefficient [31]. Therefore, in cancer therapy, one possibility to selectively and completely kill cancer cells is to induce their apoptosis over the intrinsic pathway by pharmacological intervention [31]. Targeting mitochondria with antineoplastic drugs was suggested as a novel strategy for cancer therapy [31]. Recently, I isolated a lipophilic polyammonium cation, compound **1**, as a by-product of the reaction of 1-aminoadamantane (amantadine) with 1,3-bis(chloromethyl)benzene (α,α′-dichloro-*m*-xylene) in refluxing absolute ethanol. Amantadine is known as an antiparkinsonian (*N*-methyl-D-aspartate ionotropic glutamate receptor antagonist) drug [32] and chemotherapeutic antiviral drug inhibiting influenza A virus M2 protein transmembrane proton channel [33]. This synthesis was inspired by the potential binding of a polyammonium cationic drug to DNA and/or p53 tumor suppressor protein tetramerization domain [34]. It was found that compound **1** (**PT162**, **NSC 796018**), a new compound never synthesized before [according to Chemical Abstracts Service (CAS^®^) SciFinder^®^ search], induced apoptosis in all cell lines of the National Cancer Institute (NCI) Developmental Therapeutics Program (DTP) 60-cancer cell 5-dose testing, excluding leukemia cell lines, in the micromolar range of growth inhibition 50% (**GI50**). I decided to merge compound **1** with the colchic(in)oid compound **2** (**PT166**, **NSC 750423**), which showed submicromolar **GI50** in the NCI DTP 60-cancer cell 5-dose testing but did not induce cancer cell apoptosis. Compound **2** was synthesized from colchicine and thiosemicarbazide in a one-step procedure and represented a new compound never synthesized before [according to Chemical Abstracts Service (CAS^®^) SciFinder^®^ search], just as compound **1**. Compound **1** reacted with compound **2** under the impact of sodium hydroxide (NaOH) to give compound **3**. Compound **3** (**PT167**, **NSC 799315**), a new compound never synthesized before [according to Chemical Abstracts Service (CAS^®^) SciFinder^®^ search], showed submicromolar **GI50** in the NCI DTP 60-cancer cell 5-dose testing constantly in most cell lines including leukemia cells. Importantly, compound **3** was able to induce apoptosis in all investigated cancer cells, including leukemia cell lines, with a Mean of Inhibition Data (**MID**) for total growth inhibition (**TGI**, growth inhibition 100%) of 4.57 µM and a **MID** for lethal concentration 50% (**LC50**) of 15.85 µM. I report here the chemistry and NCI DTP 60-cancer cell 5-dose testing data for compound **1**, compound **2,** and compound **3**, and demonstrate the apoptotic release of cytochrome *c* into the cytosol and activation of effector caspases induced by compounds **1** and **3**. I propose that compound **1** and compound **3** induce apoptosis according to the *Warburg* hypothesis of pre-damaged respiration as a hallmark of cancer by exploiting the defect in mitochondrial cardiolipin–cytochrome *c* association in cancer cells.

## 2. Results

### 2.1. Compound ***1***

#### 2.1.1. The Synthesis of Salt-Containing Compound **1** (**PENTA**)

To find a potential complexation and/or stabilization partner for retinazone [35,36], an attempt was made to synthesize a polyammonium polycation from the adamantane [37]-derived influenza A virus inhibitor [33] and *N*-methyl-D-aspartate (NMDA) subtype glutamate receptor antagonist [32] amantadine × HCl. For that purpose, 1-aminoadamantane hydrochloride and a 1.5-fold molar excess of 1,3-bis(chloromethyl)benzene were dissolved in aqueous ethanol. A solution of sodium hydroxide in water (3-fold molar excess) was added, and the mixture was refluxed for 3 h. Successively, acetone was added through the reflux condenser. After filtration, dilution with water, acidification with HCl, and volume reduction, the reaction mixture was extracted with ethyl acetate to remove unreacted 1,3-bis(chloromethyl)benzene. Following additional volume reduction of the aqueous phase, a crude product could be isolated by freezing. The crude product was dissolved in refluxing aqueous acetone and was hot filtrated. The filtrate was evaporated from the acetone and was acidified with HCl. Instantly, a white precipitate formed, which represented the salt-containing compound **1** (**PENTA**).

#### 2.1.2. The Synthesis of Pure Compound **1** [**PT162** (**NSC 796018**)]

**Figure 1:**
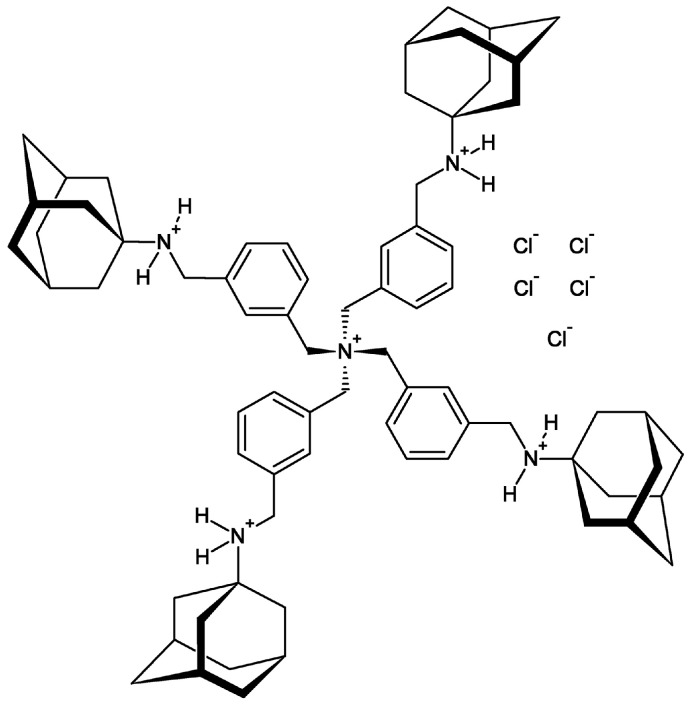
The organic chemical structure formula of compound **1** [**PT162** (**NSC 796018**)]. Four adamantan-1-ammonium cages are included within the four-bladed propeller-shaped polyammonium cation.

Pure compound **1** [**PT162** (**NSC 796018**)] (Figure 1) was synthesized from 1-aminoadamantane (amantadine) with 1,3-bis(chloromethyl)benzene (α,α′-dichloro-*m*-xylene) in refluxing absolute ethanol. Compound **1** was a by-product of the synthesis, obtained in 12.4% yield. The lipophilic main product was not isolated and removed by extraction with ethyl acetate. Crucial for the successful synthesis of compound **1** was the utilization of the free base 1-aminoadamantane instead of its commercial hydrochloride. Preceding synthesis, the free base 1-aminoadamantane was prepared from the hydrochloride by neutralization with NaOH, and this free base was used in situ for the synthesis of compound **1**.

#### 2.1.3. Structure Elucidation of Compound **1** [**PT162** (**NSC 796018**)]

**Scheme 1:**
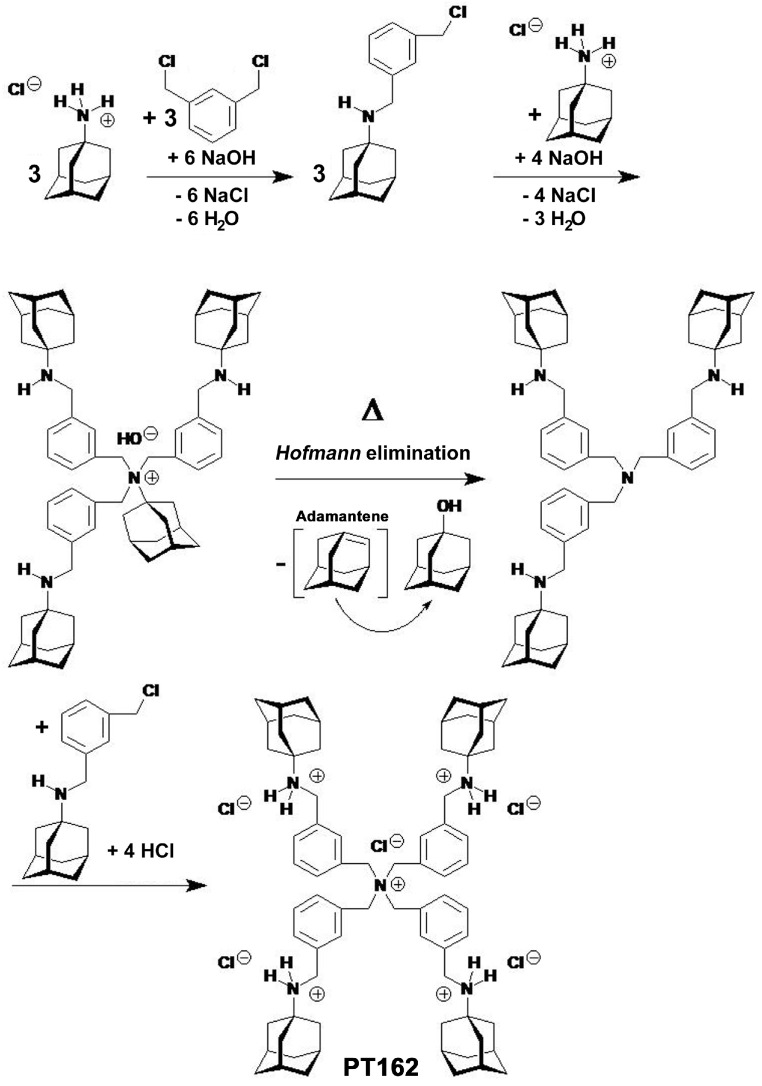
The synthesis of the polyammonium polycation compound **1** from 1-aminoadamantane × HCl and 1,3-bis(chloromethyl)benzene. The intermediate *N*-[3-(chloromethyl)benzyl]-1-adamantanamine reacted with amantadine × HCl under the influence of NaOH to *N*,*N*,*N*-tris{3-[(tricyclo[3.3.1.1^3,7^]decan-1-ylamino)methyl]benzyl}-1-adamantanammonium hydroxide, which in turn underwent *Hofmann* elimination [38] on heating at reflux under elimination of 1-adamantanol (produced from adamantene [39,40,41]). Compound **1** resulted from quaternarization with another molecule *N*-[3-(chloromethyl)benzyl]-1-adamantanamine and acidification with hydrochloric acid (HCl).

According to the structure elucidation by nuclear magnetic resonance (NMR) spectroscopy, compound **1** [**PT162** (**NSC 796018**)] was formed from the intermediate *N*-[3-(chloromethyl)benzyl]-1-adamantanamine (Scheme 1). The latter intermediate quaternarized 1-aminoadamantane to give *N*,*N*,*N*-tris{3-[(tricyclo[3.3.1.1^3,7^]decan-1-ylamino)methyl]benzyl}-1-adamantanammonium hydroxide (Scheme 1), which underwent *Hofmann* elimination [38] with 1-adamantanol being expelled. 1-Adamantanol is produced from adamantene, a known reaction intermediate [39,40,41]. The resulting tris{3-[(tricyclo[3.3.1.1^3,7^]decan-1-ylamino)methyl]benzyl}amine, in turn, was quaternarized by another *N*-[3-(chloromethyl)benzyl]-1-adamantanamine to yield, after acidification with HCl, tetrakis{3-[(tricyclo[3.3.1.1^3,7^]decan-1-ammonio)methyl]benzyl}ammonium pentachloride (compound **1**) (Scheme 1).

The structure of salt-containing compound **1** (**PENTA**) was secured by analysis of its ^1^H- (Figure S1) and ^13^C-NMR (Figure S2) spectra recorded in DMSO-*d*_6_. The proton NMR of the aliphatic part of salt-containing compound **1** (**PENTA**) was analyzed for (Figure S1): *δ* 1.62 (3 H, d; ^2^*J*_gem_ = −12.1 Hz; δ-CH*_axial_*), 1.69 (3 H, d; ^2^*J*_gem_ = −12.4 Hz; δ-CH*_equatorial_*), 2.02 (6 H, s; β-CH_2_), 2.15 (3 H, s; γ-CH), 4.10 (2 H, t; ^3^*J*_vicinal_ = 6.3 Hz; 8-CH_2_), 4.78 (2 H, s; 7-CH_2_). The proton NMR of the aromatic part of salt-containing compound **1** (**PENTA**) was analyzed for (Figure S1): *δ* 7.42–7.49 (2 H, m; H-4, H-6), 7.65 (1 H, d; ^3^*J*_ortho_ = 7.1 Hz; H-5), 7.69 (1 H, s; H-2). The three axial δ-protons of the adamantane cage were split from the corresponding three equatorial δ-protons. A broad ammonium resonance was found at *δ* 9.24 (2 H, br s; 8-NH_2_^+^ ammonium). The aliphatic resonances contained an adamantane part, two methylene groups, and an ammonium resonance in the integrated proton ratio 15:2:2:2.

**Figure 2:**
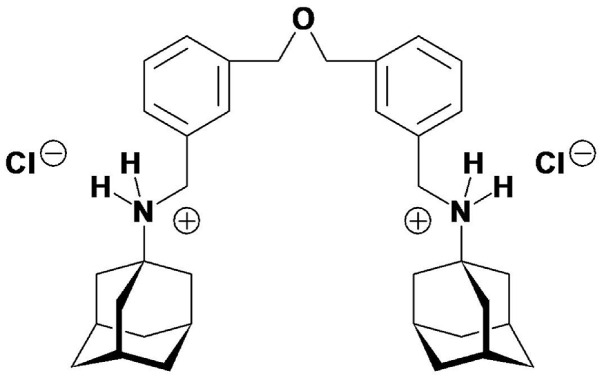
The structure of the dibenzyl ether derivative di{3-[(tricyclo[3.3.1.1^3,7^]decan-1-ammonio)methyl]benzyl}ether dichloride. This hypothetical structure was considered as an alternative reaction product in the synthesis of compound **1**.

This pointed to a symmetric ammonium molecule with two different *m*-xylylene methylene groups, which were derived from 1,3-bis(chloromethyl)benzene (*m*-xylylene dichloride). Obviously, the ammonium resonance originated from a (1-adamantyl-NH_2_-*R*)^+^ system. Therefore, only two reasonable possibilities for the structure of compound **1** (**PT162**) remained. The first would be di{3-[(tricyclo[3.3.1.1^3,7^]decan-1-ammonio)methyl]benzyl}ether dichloride (Figure 2), the second being expressed by the already given formula (Figure 1). The former structure could be ruled out for the following reasons: (i) the oxygen elemental analysis of salt-containing compound **1** (**PENTA**) showed only trace amounts of oxygen (*w* ≤ 0.82%) resulting from the ethanol and acetone traces contained in salt-containing compound **1** (**PENTA**), (ii) the absence of a commonly very strong dibenzyl ether band in the FT–IR spectrum (Figure S3) expected at wavenumber 1090 cm^−1^ for dibenzyl ether [42], (iii) the downfield methylene *δ* 4.78 (2 H, s; 7-CH_2_) did not match the typical dibenzyl ether chemical shift found at *δ* 4.54 ppm (in CDCl_3_) [43], and (iv) the analysis of the carbon-13 NMR spectrum of salt-containing compound **1** (**PENTA**) (Figure S2). The ^13^C-NMR of the aliphatic part of salt-containing compound **1** (**PENTA**) was analyzed for (Figure S2): *δ* 28.50 (γ-CH), 35.25 (δ-CH_2_), 37.35 (β-CH_2_), 42.31 (8-CH_2_), 45.84 (7-CH_2_), 57.06 (α-C). The ^13^C-NMR of the aromatic part of salt-containing compound **1** (**PENTA**) was analyzed for (Figure S2): *δ* 128.87 (C-4)*, 129.14 (C-6)*, 130.36 (C-2)*, 130.62 (C-5)*, 133.22 (C-3), 137.84 (C-1). The starred assignments are tentative and interchangeable (they could not be assigned unequivocally to the individual carbons). Clearly, the structure proof could be gained by looking at the downfield methylene *δ* 45.84 (7-CH_2_), which did not fit the typical dibenzyl ether α-CH_2_ chemical shift found at *δ* 72.10 ppm (in CDCl_3_) [43]. The 100.62 MHz ^13^C-Distortionless Enhancement by Polarization Transfer Including Detection of Quaternary Nuclei (DEPTQ) [44,45] (DEPTQ ^13^C-NMR) subspectrum (in DMSO-*d*_6_) of salt-containing compound **1** (**PENTA**) (Figure S4) secured the given CH_2_ assignments and proved that the resonance at *δ* 57.06 ppm originated from a quaternary carbon. The assignments were further verified using analysis of the gradient-selected Correlation Spectroscopy (gs-COSY) two-dimensional ^1^H–^1^H-correlation spectrum [45] (Figure S5), the gradient-selected Heteronuclear Multiple Quantum Coherence (gs-HMQC) [45] (Figure S6), and the gradient-selected Heteronuclear Multiple Bond Correlation (gs-HMBC) [45] (Figure S7) two-dimensional ^1^H–^13^C-correlation spectra of salt-containing compound **1** (**PENTA**).

Conclusively, the second structure possibility, the true formula, must be depicted by compound **1** (Figure 1) because all other possibilities would not agree with the unequivocal NMR data. Finally, the FT–IR spectrum of salt-containing compound **1** (**PENTA**) was compared to the corresponding spectrum of amantadine hydrochloride (Figure S3). Certain characteristics shared by both spectra could be recognized. The strong absorption band in salt-containing compound **1** (**PENTA**) at wavenumber 2925/2850 cm^−1^ was created by aliphatic ν(C–H) stretching vibrations. In amantadine hydrochloride this absorption could be found at wavenumber 2914/2853 cm^−1^. Aliphatic progression bands associated with these peaks could be found in both spectra. These absorptions could mainly be ascribed to the adamantane cages present in both compounds. Typical bands were found at wavenumbers 1610/1585/1459/1074 cm^−1^ [salt-containing compound **1** (**PENTA**)], in amantadine hydrochloride at wavenumbers 1626/1594/1500/1086 cm^−1^. They probably originate from the 1-adamantanammonium structure shared by both compounds. In addition, salt-containing compound **1** (**PENTA**) clearly showed the *m*-xylylene aromatic linker resonances at wavenumbers 794/762/731/693 cm^−1^, which are not seen with amantadine hydrochloride (Figure S3).

The elemental analysis of salt-containing compound **1** (**PENTA**) revealed its sodium chloride content by calculating within ±0.3% for carbon and hydrogen. The NaCl obviously was co-precipitated in the final isolation step, which is not surprising when considering the polyammonium chloride character of compound **1**. The nitrogen value was found to be 1.13% lower than calculated for C_72_H_100_N_5_Cl_5_ × 1.5 NaCl. The reason for this deficit could be the [N(CH_2_*R*)_4_]^+^ tetrasubstituted ammonium structure of the central ammonium nitrogen. Compounds of this type are known to cause combustion problems [46]. It could be speculated that the sodium content (NaCl) in salt-containing compound **1** (**PENTA**) led to the formation of only partially combustible sodium nitrate (NaNO_3_). Mutually, both effects could be responsible for the wrong nitrogen analysis of salt-containing compound **1** (**PENTA**). Since the other analytical data of salt-containing compound **1** (**PENTA**) are all very conclusive, the unsatisfactory nitrogen analysis should not be overrated. Taken together, the structure of salt-containing compound **1** (**PENTA**) could be demonstrated without doubt, especially when evaluating the unequivocal NMR data.

**Figure 3:**
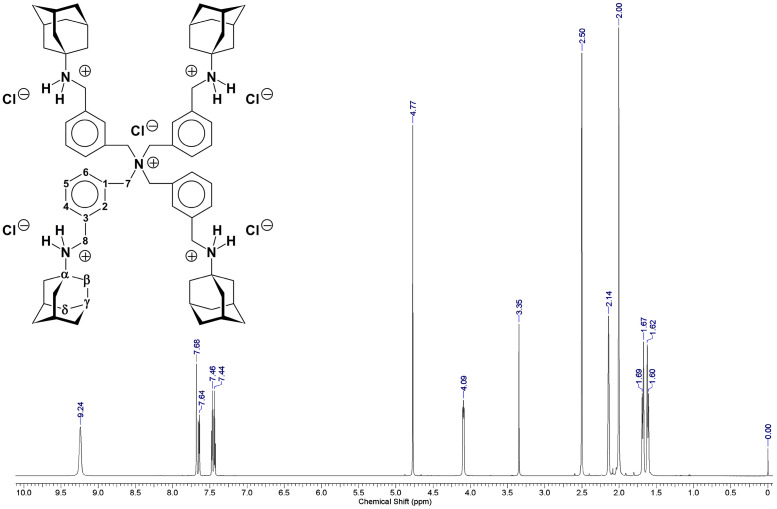
The 700.43 MHz ^1^H-NMR spectrum (in DMSO-*d*_6_) of the pure polyammonium polycation tetrakis{3-[(tricyclo[3.3.1.1^3,7^]decan-1-ammonio)methyl]benzyl}ammonium pentachloride = pure (salt-free) compound **1** [**PT162** (**NSC 796018**)] [DMSO-*d*_5_*h*_1_ quintet at *δ* 2.50 ppm, ^2^*J*_gem_ (^2^H–^1^H) = +1.9 Hz; water at *δ* 3.35 ppm].

The structure of pure (salt-free) compound **1** [**PT162** (**NSC 796018**)] was secured by ^1^H-NMR (Figure 3) and ^13^C-DEPTQ (data not shown) spectroscopy experiments, as well as elemental analysis and FT–IR spectroscopy. By ^1^H-NMR, through the integration of the proton resonance peaks, and by ^13^C-NMR, the highly symmetric structure of compound **1** was proved. Compound **1** represents a pentaammonium cation with a quaternary center and four secondary amine functions. The four functional bridges are *m*-xylylene linkers that connect the central quaternary ammonium cation chloride with four *N*-substituted 1-adamantanammonium chloride moieties. The resulting chemical structure of compound **1** is given (Figure 1). Compound **1** was registered by the National Cancer Institute (NCI) as **NSC 796018**.

### 2.2. Compound ***2***

#### 2.2.1. The Synthesis of Colchic(in)oid Compound **2** [**PT166** (**NSC 750423**)]

**Figure 4:**
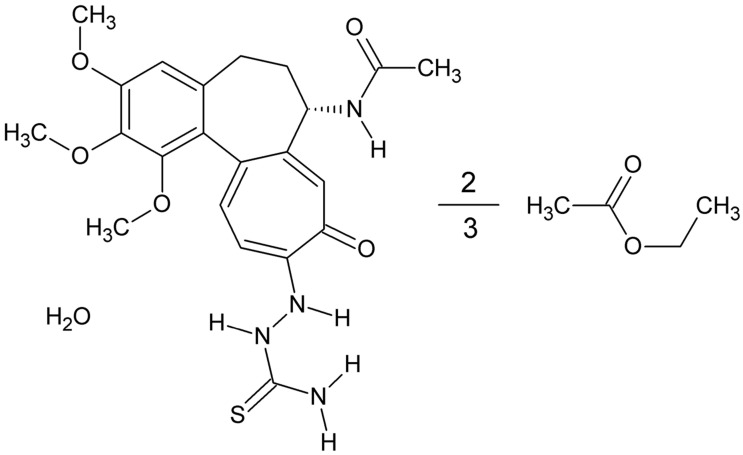
The organic chemical structure formula of compound **2** [**PT166** (**NSC 750423**)]. It contains a water of crystallization and ⅔ (ethyl acetate).

**Figure 5:**
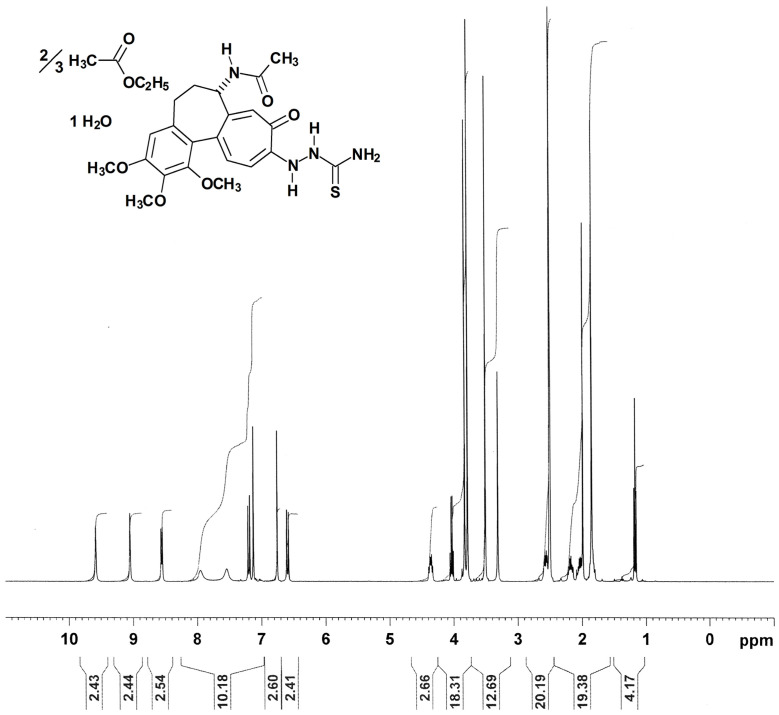
The 400.13 MHz ^1^H-NMR spectrum (in DMSO-*d*_6_) [DMSO-*d*_5_*h*_1_ quintet at *δ* 2.50 ppm, ^2^*J*_gem_ (^2^H–^1^H) = +1.9 Hz; water at *δ* 3.32 ppm] of the colchic(in)oid 10-(2-carbamothioylhydrazinyl)-10-demethoxycolchicine monohydrate × ⅔ (ethyl acetate) = compound **2** [**PT166** (**NSC 750423**)]. Downfield of *δ* 11 ppm, no resonances were detected.

Compound **2** [**PT166** (**NSC 750423**)] was synthesized from (–)-colchicine sesquihydrate (×1½ H_2_O) and thiosemicarbazide under catalysis of sodium hydroxide (NaOH) in refluxing 90% (*v*/*v*) aqueous ethanol. The structure (Figure 4) of compound **2** was secured using X-ray crystallography (see Section 2.2.3.), ^1^H-NMR (Figure 5) and ^13^C-NMR spectroscopy (data not shown) experiments, as well as elemental analysis and FT–IR spectroscopy (Figure S8). The thiosemicarbazide moiety is attached at the former position of the 10-methoxy group in colchicine. The point of connection is the terminal nitrogen of the hydrazinyl moiety of thiosemicarbazide. Compound **2** was registered by the National Cancer Institute (NCI) as **NSC 750423**.

**Scheme 2:**
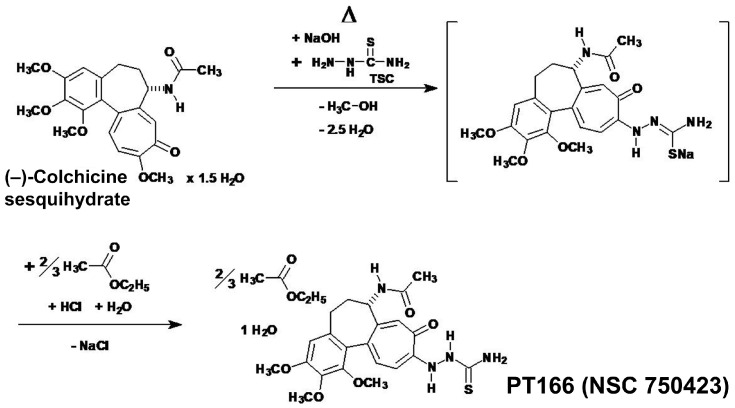
The synthesis of the modified colchic(in)oid 10-(2-carbamothioylhydrazinyl)-10-demethoxycolchicine monohydrate × ⅔ (ethyl acetate) (compound **2**, **PT166**, **NSC 750423**) by NaOH-catalyzed amidation (amino-de-alkoxylation) of the vinylogous (tropolonic) carboxylic acid methyl ester (−)-colchicine. Methanol was split off by thiosemicarbazide (TSC) from (−)-colchicine during short refluxing. Subsequently, the product compound **2** was liberated from a monosodium salt intermediate by acidification with hydrochloric acid and extraction with ethyl acetate. The term colchic(in)oid is now well accepted and documented in literature [47].

Equimolar quantities of (–)-colchicine and thiosemicarbazide were dissolved in 90% (*v*/*v*) aqueous ethanol by refluxing for 5 min. After adding a slight excess of sodium hydroxide dissolved in water, the deep orange-red solution was refluxed for 5 min. The cold, deep orange-red solution, after pre-cooling, was nearly neutralized by the dropwise addition of hydrochloric acid. Afterwards, the volume of the solution was reduced in vacuo. The reddish-brown solution was then mixed with water and acidified with aqueous hydrochloric acid. The oily emulsion was extracted with ethyl acetate (EtOAc). The separated aqueous layer (pH 2) was additionally extracted with a second volume of EtOAc. After neutralization of this aqueous phase with sodium hydrogen carbonate, the aqueous phase (pH 7–8) was extracted twice with EtOAc each. The EtOAc phases were combined and washed twice with water. The washed EtOAc phase, which already precipitated, was mixed with acetone and frozen at –25 °C. If precipitation did not start spontaneously, the volume of the solution was reduced in vacuo until coagulation started. The evolved yellow crystalline precipitate of compound **2** was filtered and dried. From the combined aqueous phases by cooling, a second crop of compound **2** could be obtained. The underlying molecular reactions for this synthesis are pictured (Scheme 2). Compound **2** was quite pure, as judged by using ^1^H-NMR spectroscopy. The representative ^1^H-NMR spectrum of compound **2** in DMSO-*d*_6_ is pictured (Figure 5).

#### 2.2.2. The Nuclear Magnetic Resonance Spectra of Compound **2**

It is known that colchic(in)oids [47] have the tendency to retain solvents, like water [48] and/or ethyl acetate [49], very firmly. Natural (–)-colchicine itself retained chloroform [50,51,52], dibromomethane/diiodomethane [53], or water as dihydrate [53,54] or sesquihydrate [52,55]. Therefore, it could be understood that compound **2** was obtained as monohydrate × ⅔ (ethyl acetate) binary solvate, as judged by using ^1^H-NMR and elemental analysis.

**Scheme 3:**
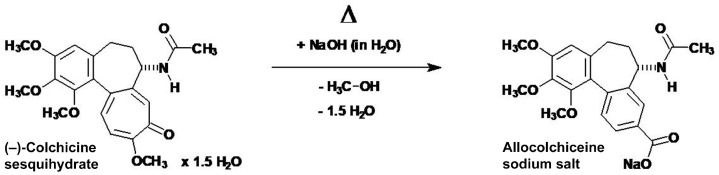
The possible side product in the synthesis of compound **2**, the allocolchiceine (syn. colchinoic acid, colchicinoic acid, colchicic acid) monosodium salt, originating from hydrolytic ring C contraction by a benzilic-type rearrangement.

The ^1^H-NMR resonances of the colchic(in)oid compound **2** were assigned with the help of literature [56,57,58,59], especially [57], which gave a complete assignment of the protons in the ^1^H-NMR spectrum of (–)-colchicine (in CDCl_3_). Compound **2** represents a completely new compound, never synthesized before [according to Chemical Abstracts Service (CAS) SciFinder^®^ (Columbus, OH, USA)]. Therefore, the proton NMR spectrum (Figure 5) of compound **2** was interpreted to the point it was possible without doubt. Aliphatic proton resonances of compound **2** dissolved in DMSO-*d*_6_ could be differentiated as: *δ* 1.18 (1.5 H, t; ^3^*J* = 7.1 Hz; O–CH_2_–C*H*_3_ ethyl acetate), 1.85 (1 H, m; H_A_-6), 1.86 (3 H, s; 17-CH_3_), 1.99 (1.5 H, s; ROOC–C*H*_3_ ethyl acetate), 2.05 (1 H, m; H_B_-6), 2.19 (1 H, m; H_A_-5), 2.57 (1 H, m; H_B_-5), 3.51 (3 H, s; 13-OCH_3_)*, 3.79 (3 H, s; 15-OCH_3_)*, 3.83 (3 H, s; 14-OCH_3_)*, 4.03 (1 H, q; ^3^*J* = 7.1 Hz; O–C*H*_2_–CH_3_ ethyl acetate), 4.37 (1 H, m; H-7). The three starred assignments (*) are tentative and interchangeable [they could not be assigned unequivocally to the individual methoxyl protons because their chemical shifts nearly coincided (Figure 5)]. The gradient-selected Correlation Spectroscopy (gs-COSY) two-dimensional ^1^H–^1^H-correlation spectrum [45] proton–proton couplings (data not shown) in connection with the gradient-selected Heteronuclear Multiple Quantum Coherence (gs-HMQC) [45] ^13^C–^1^H couplings (data not shown) gave the required information to assign the proton resonances of compound **2**. Aromatic or troponic protons in compound **2** were identified as: *δ* 6.60 (1 H, d; ^3^*J* = 11.1 Hz; H-11), 6.76 (1 H, s; H-4), 7.14 (1 H, s; H-8), 7.20 (1 H, d; ^3^*J* = 10.9 Hz; H-12). The acetamide N–H, which coupled to H-7, could be recognized at *δ* 8.56 (1 H, d; ^3^*J* = 7.6 Hz; N–H acetamide). Exchangeable protons of the thiosemicarbazide moiety, detectable in DMSO-*d*_6_, could be unequivocally assigned as: *δ* 7.56 (1 H, br s; H_2_N–C=S amino, 4′-H_A_), 7.96 (1 H, br s; H_2_N–C=S amino, 4′-H_B_), 9.06 (1 H, s; 1′-N–H), 9.59 (1 H, s; 2′-N–H). In the gradient-selected Correlation Spectroscopy (gs-COSY) two-dimensional ^1^H–^1^H-correlation spectrum [45] (data not shown) of compound **2** (in DMSO-*d*_6_), no W-shaped long-range ^4^*J* (^1^H–^1^H) coupling, known as zig-zag (W) coupling, was found. This differentiates compound **2** from the thiosemicarbazones (*E*)-4-(dimethylamino)benzaldehyde thiosemicarbazone [35] and (*E*)-4-bromo-2-fluorobenzaldehyde thiosemicarbazone [35], where such a “W” coupling was observed [35]. This pointed to sterical fixation as a prerequisite for observable W couplings in (*E*)-4-(dimethylamino)benzaldehyde thiosemicarbazone and (*E*)-4-bromo-2-fluorobenzaldehyde thiosemicarbazone, which obviously is not realized in compound **2**. This proved that compound **2** is not a thiosemicarbazone. Furthermore, the protons of the tropone (ring C) could be unequivocally assigned, and their coupling constants secured that no benzilic-type rearrangement happened to the tropolone, a reaction seen with colchic(in)oids under certain (alkaline) conditions, occasionally leading to the rearrangement products allocolchicine (colchicic acid methyl ester) [60,61] or colchicic acid (allocolchiceine) [62], both being aromatic in ring C. Under the reaction conditions employed for the synthesis of compound **2**, the allocolchiceine sodium salt (Scheme 3) could be expected as a side product, but was not observed. This benzilic-type rearrangement (Scheme 3) was elucidated by *Šantavý* [61] and *Fernholz* [62].

The ^13^C-NMR spectrum of compound **2** in DMSO-*d*_6_ was interpreted with the help of literature data on (*–*)-colchicine [57] and (*–*)-colchiceine [63]. In addition, own experimental observations were applied in the following aliphatic carbon assignments: *δ* 14.05 (O–CH_2_–C*H*_3_ ethyl acetate), 20.72 (ROOC–C*H*_3_ ethyl acetate), 22.49 (C-17, CH_3_ acetamide), 29.33 (C-5), 36.34 (C-6), 51.38 (C-7), 55.84 (14-OCH_3_)**, 59.72 (O–C*H*_2_–CH_3_ ethyl acetate), 60.62 (13-OCH_3_, 15-OCH_3_)**. The two double-starred assignments (**) are tentative and interchangeable (they could not be assigned unequivocally to the individual carbons). The aromatic and troponic carbon resonances, the carbonyl and the thiocarbonyl resonances, were detected as: *δ* 107.61 (C-4), 108.27 (C-11), 126.23 (C-8), 131.57 (C-1a), 134.26 (C-4a), 137.21 (C-12), 140.71 (C-3)***, 150.34 (C-1)***, 150.40 (C-10), 150.46 (C-12a), 152.61 (C-2)***, 152.73 (C-7a), 168.39 (C-16, HN–C=O acetamide), 170.30 (C=O ester carbonyl, ethyl acetate), 174.81 (C-9, C=O carbonyl), 181.86 (C-3′, C=S thiocarbonyl). The three triple-starred resonances (***) could not be assigned unequivocally to their individual carbon.

By these analyses, it was found that the amidation product of (*–*)-colchicine, the substituted thiosemicarbazide compound **2**, was not cyclic with regard to the thiosemicarbazide unit at ring C of compound **2**. This was quite surprising since the reaction product of (*–*)-colchicine with thiourea was cyclic with respect to the thiourea substitution in ring C [64,65], which seemed surprising, in turn, because the tropolonic C-9 carbonyl group in (*–*)-colchicine did not react with common carbonyl reagents like hydroxylamine or semicarbazide [66,67]. The reason for the latter irregularity could be the special tropylium oxide resonance type of tropones and tropolones [68,69,70,71,72]. Therefore, the synthesis of compound **2** clearly obeyed the common rules for the chemical reactivity of tropolones, whereas the synthesis of the cyclic thiourea congener [64,65] of compound **2** did not follow the common chemical reactivity experience for tropolones.

The *Fourier*–transform infrared (FT–IR) absorption spectra of the colchic(in)oid 10-(2-carbamothioylhydrazinyl)-10-demethoxycolchicine monohydrate × ⅔ (ethyl acetate) = compound **2**, and of the reference substance (−)-colchicine sesquihydrate [(−)-colchicine × 1½ H_2_O] are given in the Supplementary Information (Figure S8) for comparison.

**Figure 6:**
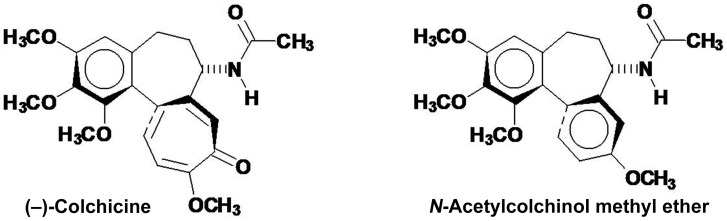
The atropisomerism of natural (–)-colchicine and its degradation product *N*-acetylcolchinol methyl ether. Both exhibit the (a*S*,7*S*)-absolute configuration, as was proved by *Brossi* et al. [73,74].

Interestingly, the natural colchic(in)oids (*–*)-colchicine and the partialsynthetic (*–*)-colchicine derivative *N*-acetylcolchinol methyl ether (Figure 6) occur in pure atropisomeric forms (Figure 6), as was elucidated by *Brossi* et al. [73,74]. The natural forms have the (a*S*,7*S*)-absolute configuration (Figure 6). The correct assignment of the absolute configuration of (*–*)-colchicine was given as (a*S*,7*S*) by *Brossi* et al. [74] according to the *Cahn–Ingold–Prelog* (CIP) rules [75,76]. The wrong (a*R*,7*S*)-absolute configuration was first postulated in 1981 during studies on tubulin binding by (*–*)-colchicine [77] and later in 1999 by *Berg* and *Bladh* [78]. The absolute configuration at C-7 was established earlier as (7*S*) [79] by the chemical degradation of natural (*–*)-colchicine to *N*-acetyl-L-glutamic acid.

Taken together, the structure of the modified colchic(in)oid compound **2** could be proved with considerable evidence, and biological effects, especially antineoplastic properties, are expected from biological testing of compound **2**. Renewed interest in colchic(in)oid research is indicated by reports on conjugating (*–*)-colchicine to vitamin B_12_ (cobalamin) [80] or paclitaxel (taxol) [81]. These colchic(in)oid conjugates were suggested for the chemotherapy of various neoplastic conditions.

#### 2.2.3. The X-ray Crystallographic Crystal and Molecular Structure Determination of Compound **2**

**Figure 7:**
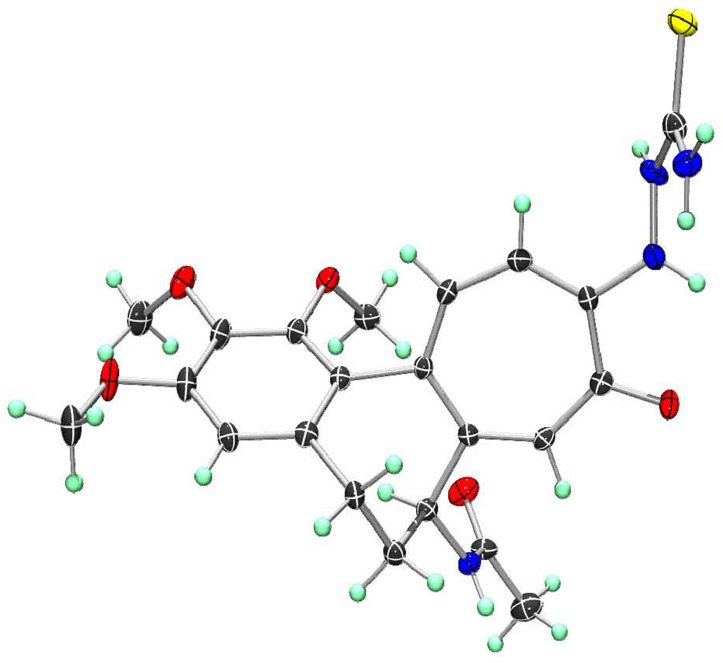
The molecular view of one independent, isolated molecule of compound **2** as found in the single crystal. Compound **2** crystallized in the monoclinic space group *P*2_1_ as sesquihydrate hemi(ethyl acetate) solvate (C_24_H_33_N_4_O_7_._50_S) [C_22_H_26_N_4_O_5_S × 1.5 H_2_O × 0.5 (C_4_H_8_O_2_)] (*Z* = 4). Compound **2** is helical stereogenic and shows the (*M*)-helicity. The plane of the thiourea moiety in the 10-(thiosemicarbazide) substituent shows a +119.1° torsion angle towards the 2-aminotropone plane.

**Figure 8:**
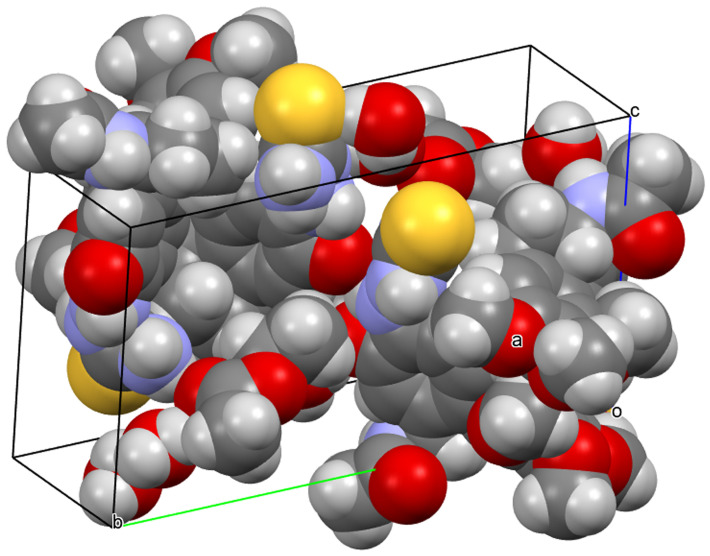
Crystal packing in the monoclinic (space group *P*2_1_) unit cell (*Z* = 2) of compound **2** crystallized as hydrate (ethyl acetate) solvate (2 C_22_H_26_N_4_O_5_S × 3 H_2_O × C_4_H_8_O_2_) C_48_H_66_N_8_O_15_S_2_ (*M* = 1059.21 g/mol). Unit cell dimensions (*a*, *b*, *c* are indicated from the origin *o*): *a* = 9.1886(5) Å, *b* = 20.9047(10) Å, *c* = 13.9841(7) Å, *α* = 90.00°, *β* = 106.153(2)°, *γ* = 90.00°, *V* = 2580.1(2) Å^3^.

**Figure 9:**
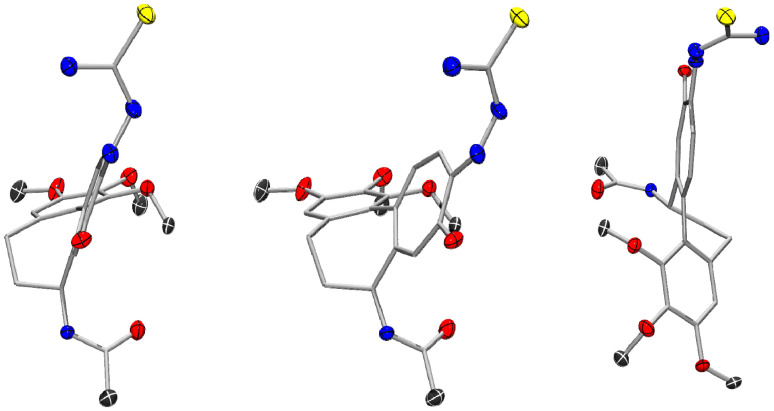
The helical axis atropisomerism view of one independent, isolated molecule of compound **2** as found in the single crystal. Compound **2**, the colchic(in)oid *N*-[(a*S*,7*S*)-10-(2-carbamothioylhydrazinyl)-1,2,3-trimethoxy-9-oxo-5,6,7,9-tetrahydrobenzo[*a*]heptalen-7-yl]acetamide, is helical stereogenic and shows the [*M(inus)*]-helicity. Priority rules [75,76] show the helical axis turn in (*S*).

Compound **2** was crystallized from ethyl acetate, and a single crystal was selected for X-ray crystallographic determination (at *T* = 100 K) of the crystal and molecular structure of compound **2** (Figure 7, Figure 8 and Figure 9, Figures S9 and S10). Compound **2** crystallized in the monoclinic space group *P*2_1_ with ethyl acetate and water of crystallization [C_22_H_26_N_4_O_5_S × 1.5 H_2_O × 0.5 (C_4_H_8_O_2_)] (*Z* = 4) (Figure S9). The crystal packing (Figure 8) with indicated hydrogen bonds (Figure S10) in the unit cell (*Z* = 2) of compound **2** is depicted. It should be noted that the molecule is helical stereogenic and shows the [*M(inus)*]-helicity as *N*-[(a*S*,7*S*)-10-(2-carbamothioylhydrazinyl)-1,2,3-trimethoxy-9-oxo-5,6,7,9-tetrahydrobenzo[*a*]heptalen-7-yl]acetamide (Figure 9).

The helical axis atropisomerism view of one independent, isolated molecule of compound **2** as found in the single crystal is depicted (Figure 9). This stands in contradiction to a report that claimed the [*P(lus)*]-helicity (a*R*) for (–)-colchicine [78]. The classification of (*M*)-helicity for compound **2** followed the *Cahn*–*Ingold*–*Prelog* (CIP) rules for the assignment of molecular helicity [75,76]. The (*M*)-helicity of (–)-colchicine was previously assigned correctly by *Brossi* et al. [74]. The X-ray crystallographic structure was deposited at The Cambridge Crystallographic Data Centre (CCDC) and assigned the deposition № **CCDC 1,839,505** (ID: **RIVGOW**). The crystal data of the X-ray crystallographic determination of the crystal and molecular structure of compound **2** are tabulated (Table 1).

Summary of the crystal data for compound **2**: C_24_H_33_N_4_O_7_._50_S [C_22_H_26_N_4_O_5_S × 1½ H_2_O × ½ (C_4_H_8_O_2_)], *M*_r_ = 529.61 g/mol, colorless plate, 0.48 × 0.31 × 0.04 mm^3^, monoclinic space group *P*2_1_, *a* = 9.1886(5) Å, *b* = 20.9047(10) Å, *c* = 13.9841(7) Å, *β* = 106.153(2)°, *V* = 2580.1(2) Å^3^, *Z* = 4, *ρ*_calcd_ = 1.363 g·cm^−3^, *μ* = 0.178 mm^−1^, *F*(000) = 1124, *T* = 100(2) K, *x_Flack_* = 0.09(6), *R*_1_ = 0.0594, *wR^2^* = 0.1325, 9686 independent reflections [2 *ϑ* ≤ 52.1°] and 693 parameters. Computer programs utilized: *APEX2* ver. 2008.3 (Bruker AXS, 2008), *Saint+* ver. 7.53A (Bruker AXS, 2008), *SHELXS97*, *SHELXL97*, *XP* ver. 5.1 (Bruker AXS, 1998).

### 2.3. Compound ***3***

#### 2.3.1. The Synthesis of Compound **3** [**PT167** (**NSC 799315**)]

**Figure 10:**
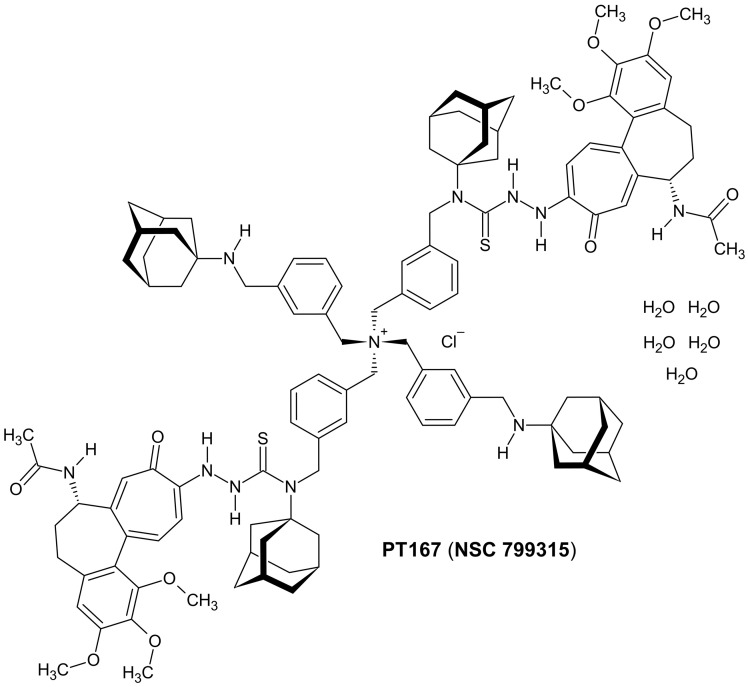
The organic chemical structure formula of compound **3** [**PT167** (**NSC 799315**)] = C_116_H_142_N_11_O_10_S_2_^+^ × Cl^−^ × 5 H_2_O = C_116_H_152_ClN_11_O_15_S_2_ (*M* = 2040.10 g/mol). The compound **3** contains one chloride anion (Cl^−^) and five water of crystallization (× 5 H_2_O).

**Scheme 4:**
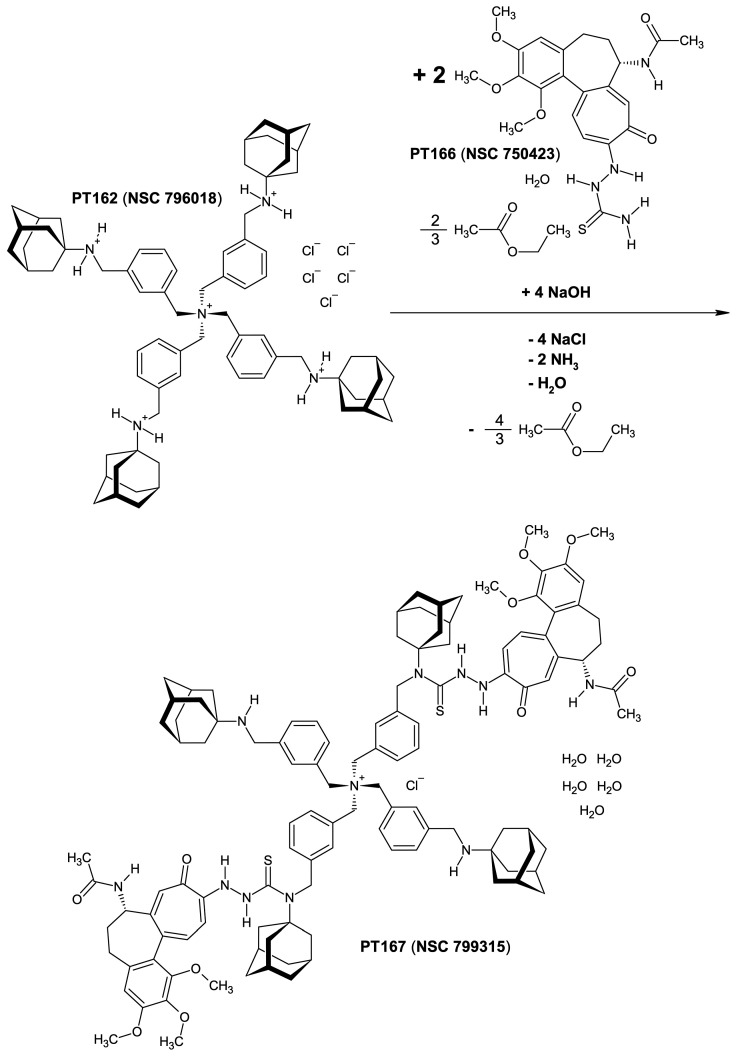
The synthesis of compound **3** = C_116_H_142_N_11_O_10_S_2_^+^ × Cl^−^ × 5 H_2_O = C_116_H_152_ClN_11_O_15_S_2_ (*M* = 2040.10 g/mol) by *trans*-(thio)amidation [35,36] catalyzed by sodium hydroxide (NaOH) at room temperature from compound **1** and compound **2**.

**Figure 11:**
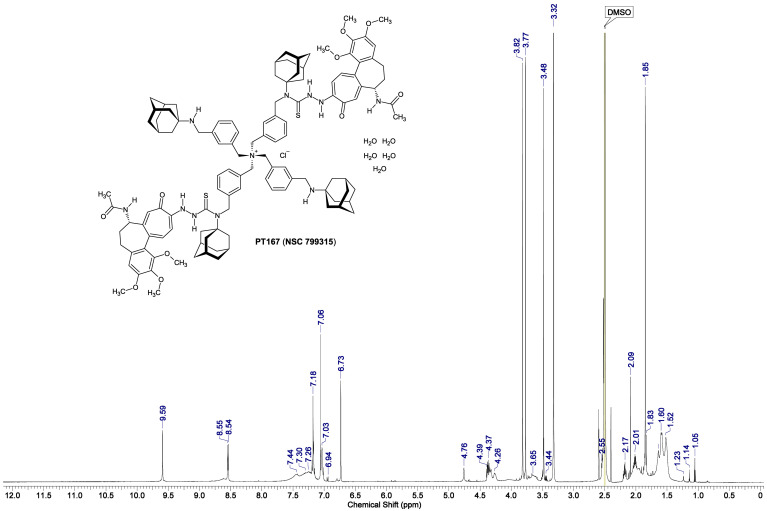
The 700.43 MHz ^1^H-NMR spectrum of compound **3** = C_116_H_142_N_11_O_10_S_2_^+^ × Cl^−^ × 5 H_2_O = C_116_H_152_ClN_11_O_15_S_2_ (*M* = 2040.10 g/mol) dissolved in DMSO-*d*_6_. Yellow marked is the solvent DMSO-*d*_6_ residual resonance [DMSO-*d*_5_*h*_1_ quintet at *δ* 2.50 ppm, ^2^*J*_gem_ (^2^H–^1^H) = +1.9 Hz] [DMSO]. The water broad singlet resonance is found at *δ* 3.32 ppm. Traces of the utilized synthesis solvents ethanol (triplet, *δ* 1.05 ppm; quartet, *δ* 3.44 ppm) and acetone (singlet, *δ* 2.09 ppm) can be detected.

**Figure 12:**
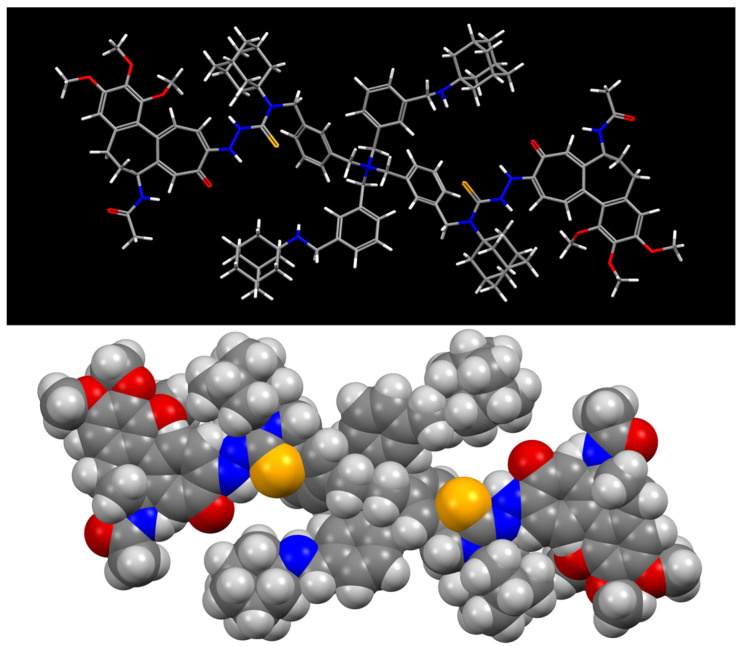
Molecular modeling {ACD/Chem Sketch version 12.01 with integrated ACD/3D Viewer (Advanced Chemistry Development, Inc., Toronto, ON, Canada) and processed with Mercury 3.1 version 3.1.1 [The Cambridge Crystallographic Data Centre (CCDC), Cambridge, UK]} of compound **3** = C_116_H_142_N_11_O_10_S_2_^+^ × Cl^−^ × 5 H_2_O = C_116_H_152_ClN_11_O_15_S_2_ (*M* = 2040.10 g/mol) without the chloride counteranion (Cl^−^) and the water of crystallization (pentahydrate). An intramolecular interaction between the β-methylene and γ-methine structural elements of the adamantanamine cages with H-11 of the 10-(thiosemicarbazide)-substituted tropone ring in compound **3** could be demonstrated.

Compound **3** [**PT167** (**NSC 799315**)] (Figure 10) was synthesized from compound **1** [**PT162** (**NSC 796018**)] and compound **2** [**PT166** (**NSC 750423**)] by *trans*-(thio)amidation catalyzed by sodium hydroxide (NaOH) at room temperature. A similar *trans*-(thio)amidation originating from a thiosemicarbazide or thiosemicarbazone moiety was observed previously in the synthesis of retinazone, a retinoid thiosemicarbazone derivative [35,36]. As a result, compound **2** was connected to compound **1** via a thioamide bonding at an adamantanamine nitrogen (Scheme 4). The molecular stoichiometry of compound **3** was determined using ^1^H-NMR (Figure 11) spectroscopy experiments, as well as elemental analysis. Compound **1** recieved two molecules of compound **2** by *trans*-amidation to yield compound **3**. The ^1^H-NMR spectrum of compound **3** (Figure 11) exhibits a peculiar resonance compression [the adamantane resonances 2.00 (24 H; β-CH_2_), 2.14 (12 H; γ-CH) could not be detected] induced by the large (macro)molecular structure of compound **3**. This points to intramolecular (hydrophobic) interaction between the β-methylene and γ-methine structural elements of the compound **1**-derived adamantanamine cages with the tropone ring (especially H-11) of the colchic(in)oid part of compound **3**. An intramolecular interaction between the β-methylene and γ-methine structural elements of the adamantanamine cages with H-11 of the 10-(thiosemicarbazide)-substituted tropone ring in compound **3** could be demonstrated using molecular modeling (Figure 12). The resulting overall chemical structure of compound **3** is given (Figure 10). Compound **3** was registered by the National Cancer Institute (NCI) as **NSC 799315**.

#### 2.3.2. The Proton Nuclear Magnetic Resonance Spectrum (^1^H-NMR) of Freshly Synthesized Compound **3**

Evidence for the formulation of compound **3** as the macromolecular entity [(bis{3-[(tricyclo[3.3.1.1^3,7^]decan-1-ylamino)methyl]benzyl}ammonio)bis(methanediylbenzene-3,1-diylmethanediyl)]di-2-[(a*S*,7*S*)-7-(acetylamino)-1,2,3-trimethoxy-9-oxo-5,6,7,9-tetrahydrobenzo[*a*]heptalen-10-yl]-*N*-(tricyclo[3.3.1.1^3,7^]decan-1-yl)hydrazinecarbothioamide chloride pentahydrate stems from the proton NMR (^1^H-NMR) spectrum of compound **3** [**PT167** (**NSC 799315**)] dissolved in deuterated dimethyl sulfoxide (DMSO-*d*_6_) (Figure 11).

The proton resonances were measured as chemical shifts *δ* (ppm): *δ* 1.48–1.68 (24 H, br m; δ-CH_2_, adamantane), 1.83 (2 H, m; H_A_-6, colch), 1.85 (6 H, s; 17-CH_3_, colch), 2.01 (2 H, m; H_B_-6, colch), 2.17 (2 H, m; H_A_-5, colch), 2.52 (2 H, m; H_B_-5, colch), 3.48 (6 H, s; 13-OCH_3_, colch)*, 3.65 (2 H, br s; secondary amine N–H), 3.77 (6 H, s; 15-OCH_3_, colch)*, 3.82 (6 H, s; 14-OCH_3_, colch)*, 4.26 (br m; 8-CH_2_, *m*-xylylene), 4.32–4.39 (2 H, br m; H-7, colch), 4.76 (s; 7-CH_2_, *m*-xylylene), 6.73 (2 H, s; H-4, colch), 7.03 (2 H, br m; H-11, colch), 7.06 (2 H, s; H-8, colch), 7.12–7.72 (br m; H-4, H-6, H-5, H-2, *m*-xylylene), 7.17 (2 H, br m; H-12, colch), 8.54 (2 H, d; ^3^*J* = 7.7 Hz; N–H acetamide, colch), 9.59 (2 H, s; 2′-N–H, hydrazinecarbothioamide) [colch = the colchic(in)oid part of compound **3**; * these assignments are tentative and interchangeable (they could not be assigned unequivocally to the individual methoxy groups); the adamantane resonances *δ* 2.00 ppm (β-CH_2_) and 2.14 ppm (γ-CH), and 1′-N–H were not detected due to paramagnetic resonance compression].

#### 2.3.3. The Proton Nuclear Magnetic Resonance Spectrum (^1^H-NMR) of Compound **3** after Six Year Storage at +0–4 °C in the Refrigerator

**Figure 13:**
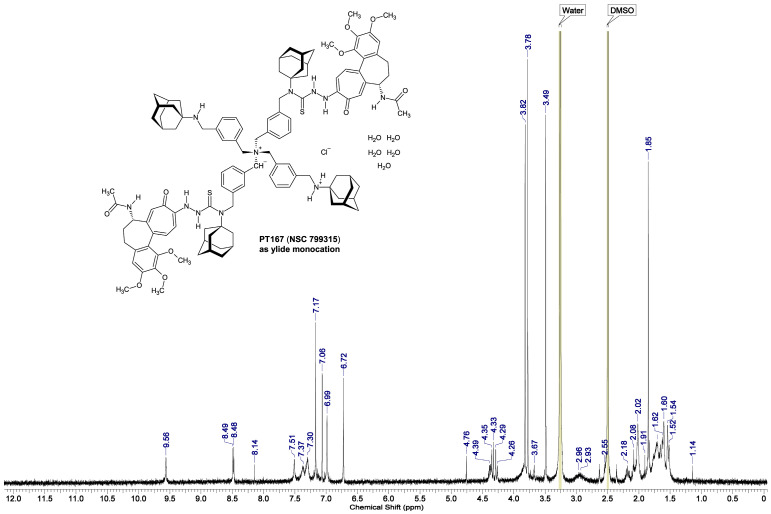
The 499.86 MHz ^1^H-NMR spectrum of newly dried (CaCl_2_, in vacuo, 19 h) compound **3** in DMSO-*d*_6_ after storing the substance over six years at +0–4 °C in the refrigerator. The spectrum was measured at *ϑ* = 35 °C on a Varian Unity INOVA 500 instrument. Yellow marked are the solvent DMSO-*d*_6_ residual resonance [DMSO] and the water broad singlet resonance at *δ* 3.25 ppm [Water]. Only traces of the utilized synthesis solvent acetone (singlet, *δ* 2.08 ppm) are present.

The specimen of compound **3,** which was synthesized on Saturday, 27 May 2017, was investigated using proton NMR (Figure 13) on Wednesday, 23 August 2023, after over six years of storage at +0–4 °C in the refrigerator. The specimen was newly dried over CaCl_2_ in vacuo for 19 h. The proton resonances were measured as chemical shifts *δ* (ppm): *δ* 1.14 (0.5 H, s; hydroxytropyl radical O–H), 1.45–1.81 (24 H, br m; δ-CH_2_, adamantane), 1.83 (2 H, m; H_A_-6, colch), 1.85 (6 H, s; 17-CH_3_, colch), 1.91 (2 H, m; H_B_-6, colch), 2.02–2.14 (12 H, br m; γ-CH), 2.18 (2 H, br m; H_A_-5, colch), 2.55 (2 H, br m; H_B_-5, colch), 2.86–3.08 [7 H, br s; 7-CH_2_, *m*-xylylene CH_2_ including ammonium ylide R-(CH^−^)N^+^(CH_2_R)_3_], 3.49 (6 H, s; 13-OCH_3_, colch)*, 3.67 (1 H, s; secondary amine N–H), 3.78 (6 H, s; 15-OCH_3_, colch)*, 3.82 (6 H, s; 14-OCH_3_, colch)*, 4.26–4.39 (8 H, br m; H-7, colch; 3 × 8-CH_2_ *m*-xylylene), 4.76 (2 H, s; 1 × 8-CH_2_, *m*-xylylene at 8-NH^+•^ radical cation), 6.72 (2 H, s; H-4, colch), 6.99 (2 H, br s; H-11, colch), 7.06 (2 H, s; H-8, colch), 7.17 (2 H, br s; H-12, colch), 7.30–7.37 (12 H, br m; H-4, H-6, H-2, *m*-xylylene), 7.51 (4 H, br s; H-5, *m*-xylylene), 8.14 (0.5 H, s; 8-NH^+•^ radical cation N–H), 8.48 (2 H, d; ^3^*J* = 7.9 Hz; N–H acetamide, colch), 9.56 (2 H, br s; 2′-N–H, hydrazinecarbothioamide) [colch = the colchic(in)oid part of compound **3**; * these assignments are tentative and interchangeable (they could not be assigned unequivocally to the individual methoxy groups); the adamantane β-CH_2_ resonance *δ* 2.00 ppm and 1′-N–H were not detected due to paramagnetic resonance compression].

The ^1^H-NMR spectrum of newly dried (CaCl_2_, in vacuo, 19 h) compound **3** in DMSO-*d*_6_ after storing the substance over six years at +0–4 °C in the refrigerator (Figure 13) convincingly proves that (i) the substance is very pure even after six years storage, (ii) exhibits the chemical structure given by me, and (iii) points to proton transfer during storage yielding the ylide monocation (ylide monohydrochloride) with discernible resonances (7 H) for the ylide R-(CH^−^)N^+^(CH_2_R)_3_.

**Figure 14:**
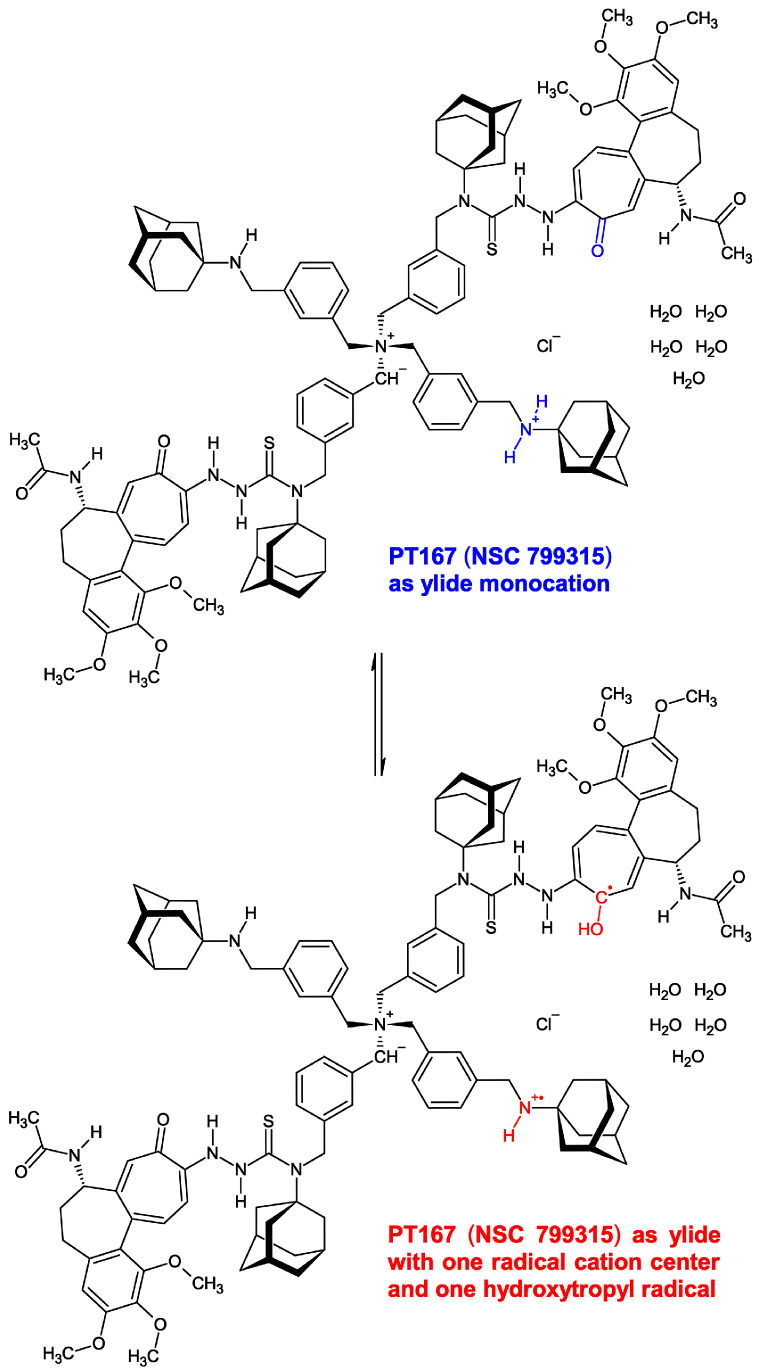
The electron transfer in compound **3** yields a partially paramagnetic molecular species. The colchic(in)oid part of the molecule picks up one electron from the ylide monocation, yielding a resonance-stabilized 1-hydroxycyclohepta-2,4,6-trien-1-yl radical (hydroxytropyl radical) [82,83] producing the 8-NH^+•^ radical cation.

This substance bears a peculiar magnetic property. During NMR spectrometer shim, it was detected that newly dried (CaCl_2_, in vacuo, 19 h) compound **3** is, in part, paramagnetic because there were considerable difficulties in shimming the NMR spectrometer probe magnetic field (personal communication *Robbin Schnieders*). The operator told me that the DMSO-*d*_6_ solution of newly dried compound **3** had to be strongly diluted with DMSO-*d*_6_ and that the spectrum acquisition time had to be elongated considerably. This pointed to the inclusion of a paramagnetic partial structure in newly dried compound **3**. Indeed, the ^1^H-NMR spectrum (Figure 13) gives evidence for that interpretation, which is depicted (Figure 14). The colchic(in)oid part of the molecule picks up one electron from the ylide monocation (ylide monohydrochloride) (Figure 14, in blue), yielding a resonance-stabilized 1-hydroxycyclohepta-2,4,6-trien-1-yl radical (hydroxytropyl radical) [82,83] producing, in consequence, the 8-NH^+•^ radical cation (Figure 14, in red).

#### 2.3.4. The Liquid Chromatographic (HPLC) Investigation of Compound **3**

**Figure 15:**
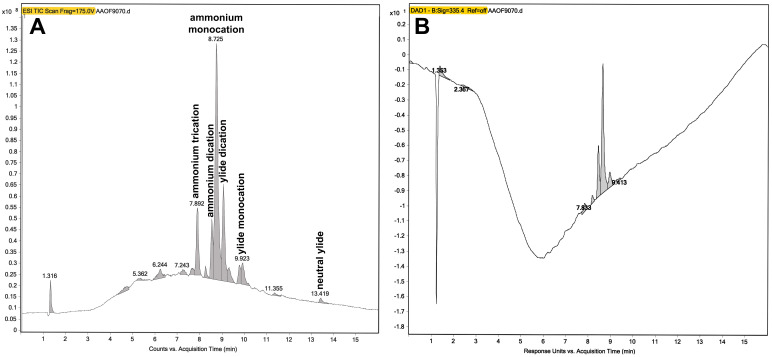
(**A**) The total ion current (TIC) chromatogram of the liquid chromatographic (HPLC)/electrospray ionization (ESI) time-of-flight (ToF) mass spectrometric investigation of compound **3** after storing the substance over six years at +0–4 °C in the refrigerator. The high-performance liquid chromatography (HPLC) of compound **3** was performed on a reversed-phase C_8_ (RP8, *n*-octyl) column with gradient elution. Indicated are the presumed multiple ionic species of compound **3** (see Table 2), which were separated due to the complicated ionization kinetics of the macromolecular substance compound **3** dissolved in acetonitrile with the presence of 0.1% (*v*/*v*) HCOOH in the HPLC eluents. (**B**) The analogous liquid chromatographic (HPLC) investigation of compound **3** with UV detection at *λ* = 335 nm.

The specimen of compound **3**, which was synthesized on Saturday, 27 May 2017, was investigated on Friday, 21 July 2023, after over six years of storage at +0–4 °C in the refrigerator. The high-performance liquid chromatography (HPLC) (Figure 15A,B) was performed with a reversed-phase C_8_ (RP8, *n*-octyl) column and gradient elution with eluent A = water/0.1% (*v*/*v*) formic acid (HCOOH) and eluent B = acetonitrile/0.1% (*v*/*v*) formic acid (HCOOH). The flowrate was 0.5 mL/min, and the linear eluent gradient was *t*_0min_ = 95% eluent A/5% eluent B to *t*_13min_ = 5% eluent A/95% eluent B, *t*_16min_ = stop. A 5 µL volume of 50 µM compound **3** solutions in acetonitrile (N≡C–CH_3_) was injected (0.25 nmol, 510.025 ng).

The total ion current (TIC) chromatogram is shown in Figure 15A, and the chromatogram with UV detection at *λ* = 335 nm is depicted in Figure 15B. Multiple ionic species of compound **3** were separated (Table 2) due to the complicated ionization kinetics of compound **3** dissolved in acetonitrile (N≡C–CH_3_) with the presence of 0.1% (*v*/*v*) HCOOH. The quaternary ammonium compound **3** can be protonated once or twice or not protonated like the in situ substance (Table 2). Moreover, compound **3** can exist in equilibrium as a neutral nitrogen ylide [84,85,86] at the central quaternary ammonium cation. This nitrogen ylide can be protonated once or twice or not protonated (neutral nitrogen ylide) (Table 2). The substance compound **3** was quite pure, as judged from the chromatogram with UV detection at *λ* = 335 nm (Figure 15B).

#### 2.3.5. The Electrospray Ionization (ESI) Mass Spectrometric Investigation of Compound **3** after HPLC Separation

**Figure 16:**
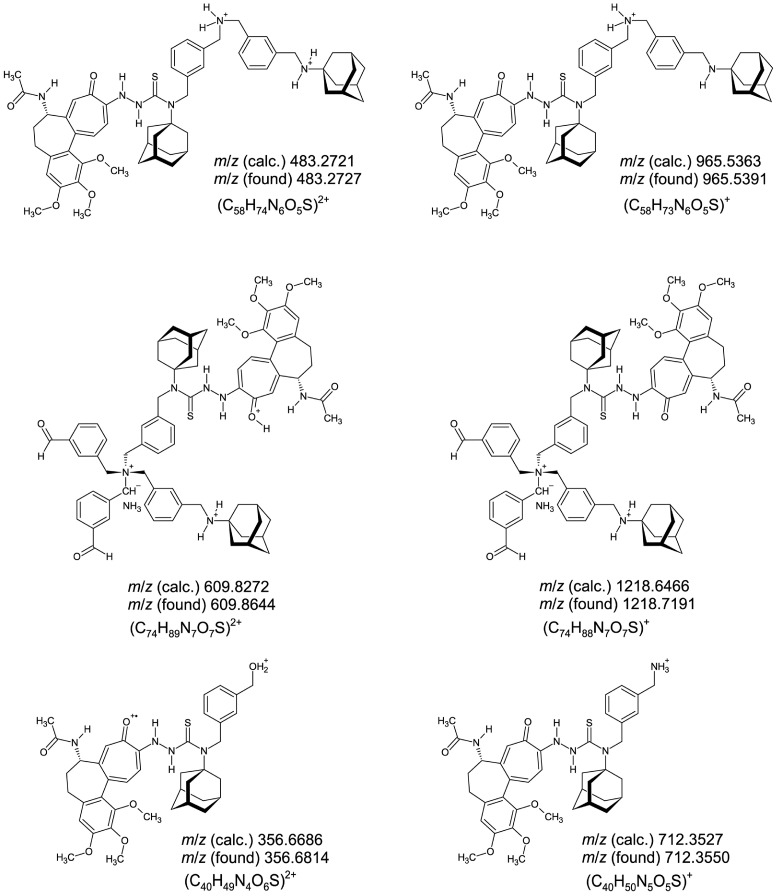
The six structure-proofing fragment cations (left, dications; right, monocations) in the electrospray ionization (ESI) time-of-flight (ToF) mass spectrometric investigation of compound **3** after storing the substance over six years at +0–4 °C in the refrigerator. The *m*/*z* 483.2727 (100%) (generated from ylide dication), *m*/*z* 965.5391 (80.9%) (generated from ylide dication), *m*/*z* 609.8644 (31.0%) (generated from ylide monocation), *m*/*z* 1218.7191 (5.0%) (generated from ylide monocation), *m*/*z* 356.6814 (19.1%) (generated from ammonium monocation), and *m*/*z* 712.3550 (100%) (generated from ammonium monocation) are the major, structure-proofing fragments of compound **3**.

The six structure-proofing fragment cations in the electrospray ionization (ESI) time-of-flight (ToF) mass spectrometry of compound **3** (Figures S11–S20) following HPLC separation on an RP8 column (LC/MS coupling) are shown in Figure 16 (left, dications; right, monocations). The cations (C_58_H_74_N_6_O_5_S)^2+^ *m*/*z* 483.2727 (100%) (generated from ylide dication), (C_58_H_73_N_6_O_5_S)^+^ *m*/*z* 965.5391 (80.9%) (generated from ylide dication), (C_74_H_89_N_7_O_7_S)^2+^ *m*/*z* 609.8644 (31.0%) (generated from ylide monocation), (C_74_H_88_N_7_O_7_S)^+^ *m*/*z* 1218.7191 (5.0%) (generated from ylide monocation), (C_40_H_49_N_4_O_6_S)^2+^ *m*/*z* 356.6814 (19.1%) (generated from ammonium monocation), and (C_40_H_50_N_5_O_5_S)^+^ *m*/*z* 712.3550 (100%) (generated from ammonium monocation) are the major, structure-proofing fragments of compound **3** created in the ESI-ToF mass spectrometer under the fragmentor voltage *V*_f_ = 175 V. The molecule cation (C_116_H_142_N_11_O_10_S_2_)^+^ *m*/*z* 1913.0382 (or a higher protonated form) was not observed due to extensive molecule fragmentation.

**Figure 17:**
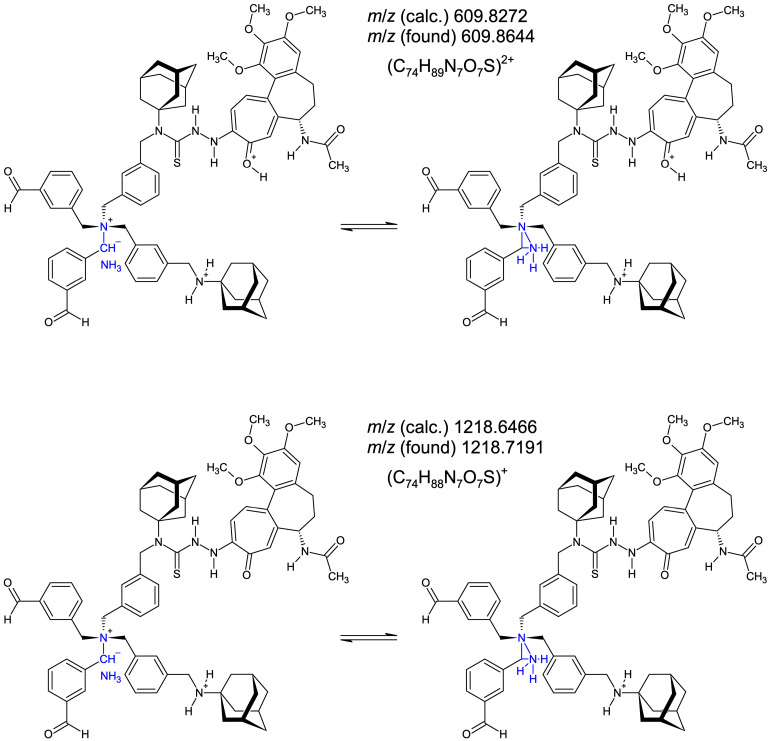
The two cations (C_74_H_89_N_7_O_7_S)^2+^ *m*/*z* 609.8644 (31.0%) [generated from compound **3** ylide monocation] and (C_74_H_88_N_7_O_7_S)^+^ *m*/*z* 1218.7191 (5.0%) [generated from compound **3** ylide monocation] (see Figure 16) are to be formulated as ammonia (NH_3_)-stabilized [84,85] nitrogen ylides [86] (left, in blue) being in equilibrium with a mass spectrometric enabled species characterized by twice pentavalent nitrogen [87,88,89] (right, in blue).

The two peaks (C_74_H_89_N_7_O_7_S)^2+^ *m*/*z* 609.8644 (31.0%) (generated from ylide monocation) and (C_74_H_88_N_7_O_7_S)^+^ *m*/*z* 1218.7191 (5.0%) (generated from ylide monocation) (Figure 16) are to be formulated as ammonia (NH_3_) coordination-stabilized [84,85] nitrogen ylides [86] being in equilibrium with a mass spectrometric generated species exhibiting pentavalent nitrogen [87,88,89] according to Figure 17. The fragment cations (C_18_H_21_NNaO)^+^ *m*/*z* 290.17 [(3-{[(adamantan-1-yl)imino-κ*N*]methyl}benzaldehyde-κ*O*)sodium(1+)], (C_8_H_7_O_2_)^+^ *m*/*z* 135.12 [protonated isophthal(di)aldehyde], (C_8_H_11_O)^+^ *m*/*z* 123.08 [protonated *m*-xylyl alcohol (3-methylbenzyl alcohol)], and (C_16_H_21_N)^+•^ *m*/*z* 227.20 [*N*-phenyladamantan-1-amine radical cation], accompany the greater fragments pointing to extensive fragmentation force under the fragmentor voltage *V*_f_ = 175 V. The *N*-phenyladamantan-1-amine radical cation results from 1-{[(adamantan-1-yl)amino]methyliumyl}cyclopenta-2,4-dien-1-ide (C_16_H_21_N, a zwitterionic fulvene) created from the benzyl species *N*-{[3-(aminomethyl)phenyl]methyl}adamantan-1-amine (C_18_H_26_N_2_) through the loss of C_2_H_5_N and fulvene-to-benzene rearrangement [90,91].

In summary, the chemical structure of compound **3** could be substantiated as a derivative of colchiceine hydrazide (10-hydrazinyl-10-demethoxycolchicine) [64,65] bridged over a thiocarbonyl to the compound **1** core, being quite pure. None of compound **2** and compound **1**, the synthesis starting substances, or colchiceine hydrazide, a possible degradation product, could be detected in the compound **3** preparation using highly sensitive detection methods.

### 2.4. NCI 60 Cell Five-Dose Screen with the Drugs Compound ***1***, Compound ***2***, and Compound ***3***

#### 2.4.1. National Cancer Institute (NCI) Developmental Therapeutics Program (DTP) 60-Cancer Cell 5-Dose Testing

The NCI DTP 60-cancer cell 5-dose testing is a free-of-charge, standardized oncologic test platform offered publicly by NCI as part of the National Institutes of Health (NIH) and financed by the United States of America government (U.S. Department of Health and Human Services). The results of the antineoplastic in vitro screening on 60 standard tumor cell lines of test substances delivered to NCI DTP are obtained as a summary consisting of four pages, which are described here:

**First page:** graphic allover presentation of the second page with **All Cell Lines** in one graphic and molar concentrations expressed in logarithmic log_10_ unit. The earlier the colored curves aspire to the bottom, the more potent the drug. The more the curves strive from ±0% to −100%, the more the tumor is killed by the drug.

**Second page:** inhibition curves for tumor cell lines summarized in terms of tumor type. The **Sample Concentrations** are given at the *x*-axis in log_10_ units (−9 = 1 nM, −8 = 10 nM, −7 = 100 nM, −6 = 1 µM, −5 = 10 µM, −4 = 100 µM, −3 = 1000 µM = 1 mM). The **Percentage Growth** is given at the y-axis in % and spans from +100% to –100%. **0% Growth** means that the tumor is still there but not growing anymore. **−100% Growth** means that the tumor cells are all dead and had died by apoptosis or necrosis; this is the ideal outcome. The smoother the curve, the more reliable the tumor inhibition. Generally, it is not sufficient to reach only **0% Growth** since the tumor is still there. The ideal is **−100% Growth** since the tumor cells were completely killed by the drug, but only a few antineoplastic drugs in clinical use reach this. Cytostatics in clinical use generally reach only **0% Growth**; this is called cytostasis (therefore the name cytostatics).

**Third page:** the actual concentrations used in log_10_ unit are given for five doses (Note: most of these are not smooth values, but technically created values). Then, the mean optical densities of the vital stain sulforhodamine B (SRB) retained in the cells are given. The higher the SRB optical density, the higher the number of living tumor cells. Then, the **Percent Growth** is given again. The **GI50** (**Growth Inhibition 50%**), **TGI** (**Total Growth Inhibition = 0% Growth**), and **LC50** (**Lethal Concentration 50% =** −**50% Growth**) are given in linear concentration (E–6 = 10^−6^ M = µM, E–5 = 10^−5^ M…) units. A > 10^−X^ (for example: > 10^−4^ = > 100 µM) means that the corresponding defined criterion is not reached by the drug = failure to reach the defined tumor inhibition criterion (**GI50**, **TGI**, or **LC50**). The **NSC number** of the test drug is given in the heading. The **NSC Number** is a standardized system of all anticancer compounds tested by NCI. The **NSC number** can be used for unequivocal identification and definition of all anticancer drugs if registered and tested by NCI.

**Fourth page:** this is the summary of the results expressed in log_10_ units. The defined tumor inhibition criterion (**GI50**, **TGI**, or **LC50**) is recalculated in log_10_ expression, and the colored bars, which indicate then the sensitivity of the individual tumor cell line to the agent, are given in log_10_ units. **Bar to the left: less sensitive than mean**; **Bar to the right: more sensitive than mean**. The most important feature is at the bottom of the page: the **MID** (**Mean of Inhibition Data**) indicates the mean concentration for all tested cell lines required for the drug to reach the defined tumor inhibition criterion (**GI50**, **TGI**, or **LC50**). It is given in **Log_10_GI50**, **Log_10_TGI**, and **Log_10_LC50**. From those values, the corresponding **MID** is calculated. The more negative the **MID**, the more potent the drug. The **MID** can be transformed from logarithmic into linear concentrations by the formula: *c* = 10**^MID^**. The **Delta** and **Range** of the **MID** correspond to these definitions, expressed in logarithmic log_10_ unit:

The **Delta** is defined as:

**Delta** = **Mean** − **Growth Percent** of the drug’s most inhibited cell line

The **Range** is defined as:

**Range** = (**Growth Percent** of the drug’s least inhibited cell line − **Mean**) + **Delta**

#### 2.4.2. Overall NCI 60 Cell Five-Dose Screen Results with the Drugs Compound **1**, Compound **2**, and Compound **3**

Compound **1** (**PT162**, **NSC 796018**), compound **2** (**PT166**, **NSC 750423**), and compound **3** (**PT167**, **NSC 799315**) were screened in the NCI DTP 60-cancer cell 5-dose testing program. The results are summarized in Table S1. Compound **1** and compound **3** generally started to inhibit cancer cell growth in the submicromolar range, whereby compound **3** was slightly more potent than compound **1**. The responses of compound **1** and compound **3** regarding inhibition of cancer cell growth were remarkably smooth, regular, and consistent. Nearly all cancer cell lines were inhibited by compound **1** and compound **3** in a very consistent fashion, including leukemia cell lines for compound **3**. In contrast, the inhibiting effect of compound **2** on cancer cell growth was widely variable. Importantly, compound **1** and compound **3**, including leukemia cell lines for compound **3**, induced consistent cancer cell death in almost all cancer cell lines. In contrast, compound **2** failed to induce cancer cell death in nearly all cancer cell lines. The **GI50** for compound **1** was 1.288 µM, for compound **2** 0.933 µM, and for compound **3** 1.349 µM (Table S1). The **TGI** for compound **1** was 4.677 µM, for compound **2** 32.359 µM, and for compound **3** 4.571 µM (Table S1). The **LC50** for compound **1** was 16.596 µM, for compound **2** 95.499 µM, and for compound **3** 15.849 µM (Table S1). As can be clearly seen from these data, compound **2** failed to induce cancer cell death and acts only cytostatic, whereas compound **1** and compound **3** successfully induced cancer cell death to nearly −100% cancer cell growth and can be classified as tumoricidal.

Compound **1** and compound **3** were consistently active versus wild-type p53-containing cancer cell lines and cancer cell lines with mutant or lost p53 protein (Table S1). The p53 status of the individual cancer cell lines in the NCI DTP 60-cancer cell 5-dose testing cell line panel was taken as published [92,93].

#### 2.4.3. National Cancer Institute (NCI) Developmental Therapeutics Program (DTP) 60-Cancer Cell 5-Dose Testing Results with Compound **1**

**Figure 18:**
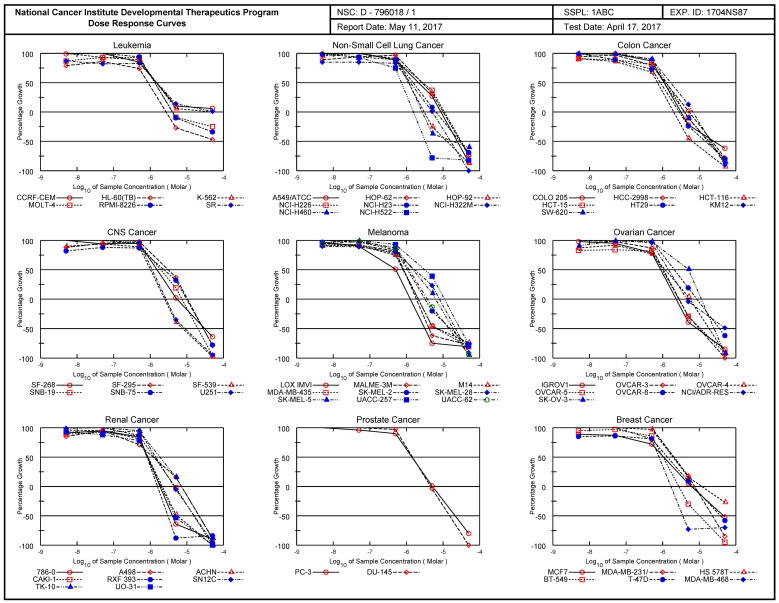
Graphic presentation of the antineoplastic activity exhibited by the pure drug compound **1** (**PT162**, **NSC 796018**) in the National Cancer Institute (NCI) Developmental Therapeutics Program (DTP) 60-cancer cell 5-dose testing with the inhibition curves for sixty human tumor cell lines arranged/ordered in terms of general tumor type.

The four pages of the National Cancer Institute (NCI) Developmental Therapeutics Program (DTP) 60-cancer cell 5-dose testing results for compound **1** (**PT162**, **NSC 796018**) are given in succession. **First page** (Figure S21): graphic allover presentation of second page with **All Cell Lines** in one graphic. **Second page** (Figure 18): inhibition curves for tumor cell lines arranged/ordered for general tumor type. **Third page** (Figure S22): the mean optical densities of the utilized vital stain sulforhodamine B (SRB) retained in the cells, the actual used concentrations in log_10_ units for five doses, and the **Percent Growth** are given. The **GI50** (**Growth Inhibition 50%**), **TGI** (**Total Growth Inhibition = 0% Growth**), and **LC50** (**Lethal Concentration 50% = −50% Growth**) are given in linear concentration units. **Fourth page** (Figure S23): this is the summary of the results expressed in log_10_ units. The defined tumor inhibition criterion (**GI50**, **TGI**, or **LC50**) is recalculated in log_10_ expression, and the colored bars, which indicate then the sensitivity of the individual tumor cell line to the agent, are given in log_10_ units.

#### 2.4.4. National Cancer Institute (NCI) Developmental Therapeutics Program (DTP) 60-Cancer Cell 5-Dose Testing Results with Compound **2**

**Figure 19:**
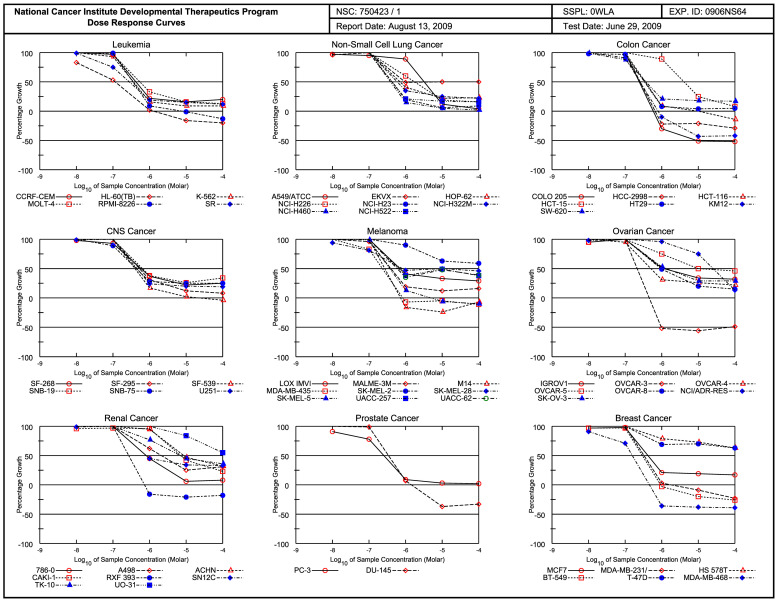
Graphic presentation of the antineoplastic activity exhibited by the drug compound **2** (**PT166**, **NSC 750423**) in the National Cancer Institute (NCI) Developmental Therapeutics Program (DTP) 60-cancer cell 5-dose testing with the inhibition curves for sixty human tumor cell lines arranged/ordered in terms of general tumor type.

The four pages of the National Cancer Institute (NCI) Developmental Therapeutics Program (DTP) 60-cancer cell 5-dose testing results for compound **2** (**PT166**, **NSC 750423**) are given in succession. **First page** (Figure S24): graphic allover presentation of second page with **All Cell Lines** in one graphic. **Second page** (Figure 19): inhibition curves for tumor cell lines arranged/ordered for general tumor type. **Third page** (Figure S25): the mean optical densities of the utilized vital stain sulforhodamine B (SRB) retained in the cells, the actual used concentrations in log_10_ units for five doses, and the **Percent Growth** are given. The **GI50** (**Growth Inhibition 50%**), **TGI** (**Total Growth Inhibition = 0% Growth**), and **LC50** (**Lethal Concentration 50% = −50% Growth**) are given in linear concentration units. **Fourth page** (Figure S26): this is the summary of the results expressed in log_10_ units. The defined tumor inhibition criterion (**GI50**, **TGI**, or **LC50**) is recalculated in log_10_ expression, and the colored bars, which indicate then the sensitivity of the individual tumor cell line to the agent, are given in log_10_ units.

#### 2.4.5. National Cancer Institute (NCI) Developmental Therapeutics Program (DTP) 60-Cancer Cell 5-Dose Testing Results with Compound **3**

**Figure 20:**
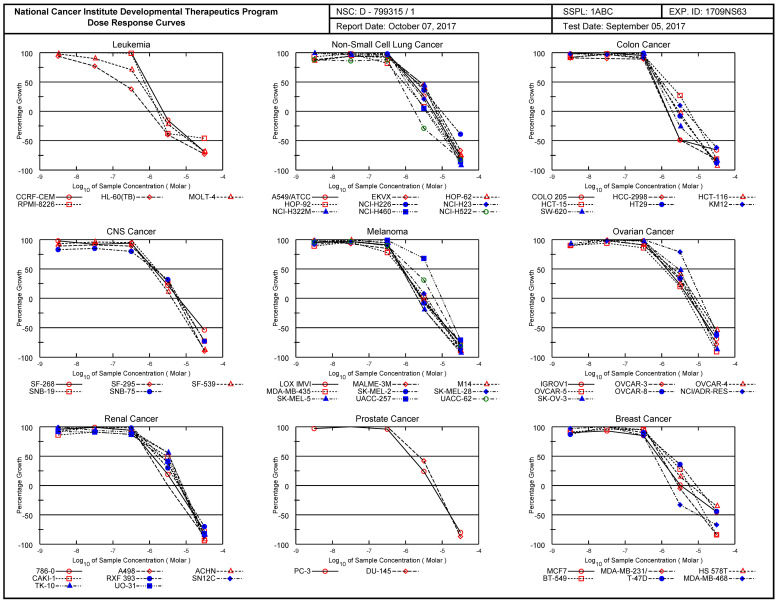
Graphic presentation of the antineoplastic activity exhibited by the drug compound **3** (**PT167**, **NSC 799315**) in the National Cancer Institute (NCI) Developmental Therapeutics Program (DTP) 60-cancer cell 5-dose testing with the inhibition curves for sixty human tumor cell lines arranged/ordered in terms of general tumor type.

The four pages of the National Cancer Institute (NCI) Developmental Therapeutics Program (DTP) 60-cancer cell 5-dose testing results for compound **3** (**PT167**, **NSC 799315**) are given in succession. **First page** (Figure S27): graphic allover presentation of second page with **All Cell Lines** in one graphic. **Second page** (Figure 20): inhibition curves for tumor cell lines arranged/ordered for general tumor type. **Third page** (Figure S28): the mean optical densities of the utilized vital stain sulforhodamine B (SRB) retained in the cells, the actual used concentrations in log_10_ units for five doses, and the **Percent Growth** are given. The **GI50** (**Growth Inhibition 50%**), **TGI** (**Total Growth Inhibition = 0% Growth**), and **LC50** (**Lethal Concentration 50% = −50% Growth**) are given in linear concentration units. **Fourth page** (Figure S29): this is the summary of the results expressed in log_10_ units. The defined tumor inhibition criterion (**GI50**, **TGI**, or **LC50**) is recalculated in log_10_ expression, and the colored bars, which indicate then the sensitivity of the individual tumor cell line to the agent, are given in log_10_ units.

### 2.5. Cytochrome c Assay (Mitochondrial and Cytosolic) with the Drugs Compound ***1***, Compound ***2***, and Compound ***3*** in the Human Prostate Cancer Cell Lines PC-3 and DU-145

**Figure 21:**
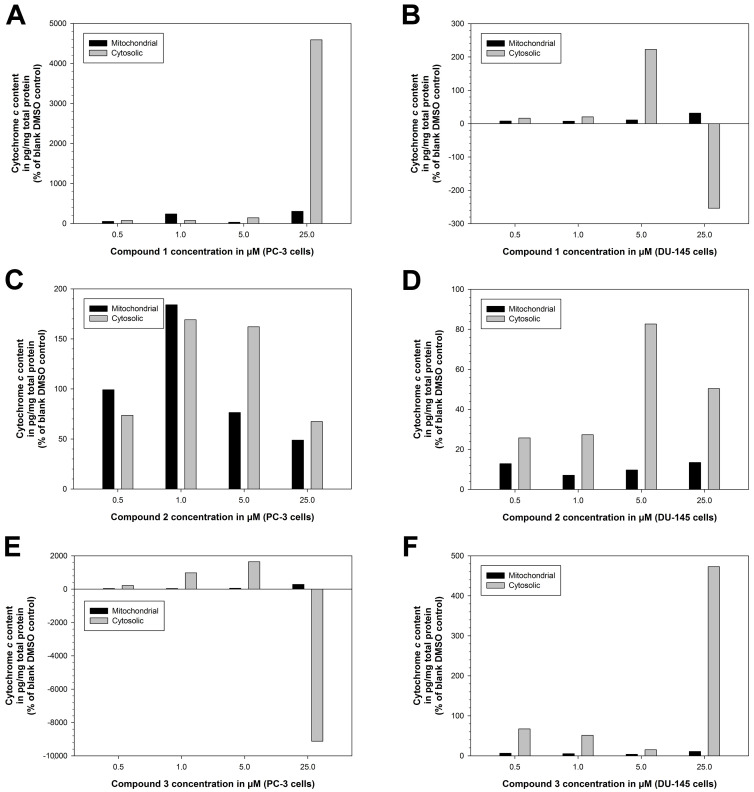
Compound **1** (**PT162**, **NSC 796018**) and compound **3 (PT167, NSC 799315)** induce cytochrome *c* translocation from mitochondria into the cytosol, a hallmark of the intrinsic pathway of apoptosis, whereas compound **2** (**PT166**, **NSC 750423**) fails to induce apoptotic cytochrome *c* translocation. The cytochrome *c* assays (mitochondrial and cytosolic) were performed with compounds **1**, **2,** and **3** in the human prostate cancer cell lines PC-3 and DU-145. The protein content for each cellular fraction was calculated to determine the mass content of cytochrome *c* in each fraction (pg cytochrome *c*/mg total protein). The mass content of cytochrome *c* (pg cytochrome *c*/mg total protein) was normalized towards the corresponding blank DMSO control and is given in percent (%) of blank DMSO control. Each experiment was representative of the effects of compound **1**, compound **2**, and compound **3** in the human prostate cancer cell lines PC-3 and DU-145. (**A**) Experiment compound **1** in PC-3 cells. (**B**) Experiment compound **1** in DU-145 cells. (**C**) Experiment compound **2** in PC-3 cells. (**D**) Experiment compound **2** in DU-145 cells. (**E**) Experiment compound **3** in PC-3 cells. (**F**) Experiment compound **3** in DU-145 cells.

To assess the nature of the cancer cell death-inducing effect of the drugs compound **1** (**PT162**, **NSC 796018**), compound **2** (**PT166**, **NSC 750423**), and compound **3** (**PT167**, **NSC 799315**), and to verify the failure to induce cancer cell death of compound **2**, the cellular compartment of cytochrome *c* in the prostate cancer cell lines PC-3 and DU-145 under the action of compound **1**, compound **2**, and compound **3** was investigated (Figure 21). Compound **1** readily induced cytochrome *c* translocation from mitochondria into the cytosol at 25.0 µM concentration in PC-3 cells (Figure 21A) and 5.0 µM concentration in DU-145 cells (Figure 21B).

Compound **2** failed to induce a significant difference in the cytochrome *c*-residing cellular compartment in PC-3 cells (Figure 21C) and, less significantly, in DU-145 cells (Figure 21D). Compound **3** readily induced cytochrome *c* translocation from mitochondria into the cytosol at 5.0 µM concentration in PC-3 cells (Figure 21E) and 25.0 µM concentration in DU-145 cells (Figure 21F). At 25.0 µM concentration, compound **1** in DU-145 cells (Figure 21B) and compound **3** in PC-3 cells (Figure 21E) both induced complete cell death, which resulted in massive depletion of cytochrome *c* in the cytosol, very probably by apoptotic degradation of cytochrome *c* protein by effector caspases. That means that cytochrome *c* apoprotein is digested by the cytosolic caspases executing apoptosis.

### 2.6. HIV-1_LAI_ Replication Reverse Transcriptase Assay with the Drug Compound ***1*** in Primary PBL Cells

Compound **1** (**PT162**, **NSC 796018**) is inhibitory towards human immunodeficiency virus type 1 (HIV-1) strain LAI (HIV-1_LAI_) (= HIV-1_BRU_ = LAV-1) replication in freshly explanted, primary human peripheral blood lymphocytes (PBL cells) (Table 3). The effective inhibitory concentration 50% (EC_50_) in PBL cells was 0.56 µM, and the effective inhibitory concentration 90% (EC_90_) in PBL cells was 4.3 µM. The cytotoxic concentration of 50% (CC_50_) for the PBL cells was 2.2 µM; this yields a selectivity index of 50% (SI_50_) = CC_50_/EC_50_ of 3.9. The *r*^2^ coefficient of determination (*r*^2^ measure of goodness-of-fit) on EC_50_ and EC_90_ was 0.93. As a positive control, AZT (zidovudine, 3′-azido-3′-deoxythymidine) was served. The given effective inhibitory concentrations (μM ± s.d.) for the positive control AZT were averaged and treated statistically from four (*n* = 4) independent determinations (Table 3). Furthermore, the cytotoxicity of compound **1** (**PT162**, **NSC 796018**) towards CCRF−CEM cells (ATCC^®^ CCL-119™, acute lymphoblastic leukemia cells, tumorigenic CD4^+^ T lymphoblasts) [94] and Vero cells [African green monkey (grivet) *Chlorocebus aethiops* (syn. *Cercopithecus aethiops*) kidney epithelial cells] was determined (Table 3). The cytotoxic concentration of 50% (CC_50_) for the CCRF−CEM cells was < 1.0 µM with 60.0% growth inhibition of CCRF−CEM cells at 1.0 µM. The cytotoxic concentration of 50% (CC_50_) for the Vero cells was 1.8 µM.

The inhibitory action of p53 on HIV-1 replication is well established [95,96,97]. Compound **1** (**PT162**, **NSC 796018**) clearly inhibits HIV-1_LAI_ replication by inducing p53 and its associated cyclin-dependent kinase inhibitor p21^Waf1^/p21^Cip1^ [98]. The latter factor, p21^Waf1^/p21^Cip1^, is induced by p53 activation [98] and inhibits HIV-1 replication [99,100]. In addition, tumor suppressor protein p53 interacts with HIV-1 *trans*-activator protein Tat (*trans*-activator of transcription) [101,102,103], HIV-1 Nef (negative regulatory factor) accessory protein [104], and HIV-1 Vpr (viral protein R, viral protein rapid) accessory protein [100,105]. The marked cytotoxic action of compound **1** (**PT162**, **NSC 796018**) on CCRF−CEM acute lymphoblastic leukemia cells clearly stems from its strong antileukemic/antineoplastic activity.

## 3. Discussion

p53 was identified in 1979 by *Sir David P. Lane* and *Lionel V. Crawford* [106], *Arnold J. Levine* and *Daniel I.H. Linzer* [107], and *Lloyd J. Old*, *Albert B. DeLeo* and Colleagues [108]. p53 had been assumed before to exist as the target of the SV40 virus (*Polyomaviridae*, *Betapolyomavirus*; simian vacuolating virus 40, simian virus 40), which is tumorigenic. SV40 virus’ oncogenic potential involves suppression of the transcriptional properties of p53 [106,107] (and retinoblastoma) tumor suppressor protein(s) by the SV40 large T antigen [106,107]. The name p53 was coined in 1979 [108], describing the apparent molecular mass of the protein on sodium dodecyl sulfate-polyacrylamide gel electrophoresis (SDS−PAGE). p53 plays an important role in the regulation of, or progression through, the cell cycle, in apoptosis and maintenance of genomic stability, by several mechanisms:

p53 can activate DNA repair proteins when DNA has suffered sustained damage. Thus, it may be an important factor in aging.

p53 can arrest growth by holding the cell cycle at the G_1_/S boundary on DNA damage recognition—if it stops the cell cycle here for a long enough time, the DNA repair machinery will have time to fix the damage, and the cells will be allowed to continue the cell cycle.

p53 can initiate apoptosis (genetically programmed cell death) if DNA damage proves to be irreparable.

p53 is essential for the senescence response to shortened telomeres.

p53 acts as an inhibitor of angiogenesis generally mediated by vascular endothelial growth factor A (VEGF-A).

p53 plays an important role in the differentiation and maintenance of human stem cells throughout embryonal development and adult human life.

Compound **1** induced the expression of the p53-induced cyclin-dependent kinase inhibitor Waf1/Cip1/Sdi1/CAP20/MDA-6/CDKN1A (p21^Waf1^/p21^Cip1^) [98], and downregulated the p53-suppressed cyclin D1 [109,110,111] protein factor in metastatic castration-resistant prostate cancer (mCRPC) cells, multiple myeloma U266 B lymphocyte lymphoblast cells and plasmacytoma RPMI-8226 B lymphocyte lymphoblast cells (data not shown). Moreover, compound **1** increased the p53-induced pro-apoptotic factor Bax expression in these cancer cells (data not shown). This is a clear indication of (re)activation of (mutant) p53 function by compound **1** in the tested cancer cells.

**Figure 22:**
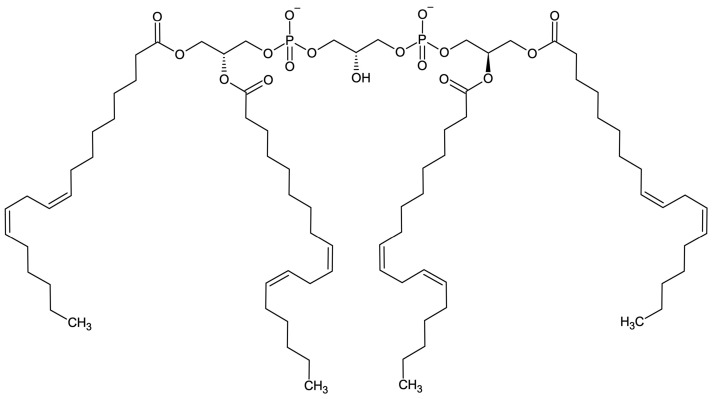
The organic chemical structural formula of cardiolipin [1,3-bis(*sn*-3′-phosphatidyl)-*sn*-glycerol, 1,3-diphosphatidyl-*sn*-glycerol]. The molecule is optically active, representing the L,L-form. Pictured is the predominant anionic species in the inner mitochondrial membrane with C_18:2_ [linoleic acid, (9*Z*,12*Z*)-9,12-octadecadienoic acid] at each of the four acyl positions, as isolated from bovine heart, bovine heart cardiocyte mitochondria, and rat liver [118].

Compound **1** and compound **3** have, therefore, three antineoplastic mechanisms-of-action: (i) their binding to (mutant) p53 [106,107,108,112] core DNA-binding domain (DBD) [112], thereby restoring sequence-specific DNA-binding of p53 mutants, inhibiting mutant p53 prion-like amyloid aggregation, restoring Zn^2+^-binding to mutant p53 DBD, and reactivating wild-type p53 transcriptional activity, (ii) their binding to the tetramerization/oligomerization domain (OD) of mutant p53 [34,112] by ionic and hydrophobic interaction(s), thereby restoring the tetramerization of OD mutant p53, reinstalling the target promoter DNA-binding and transcriptional activation properties of OD mutant p53, and (iii) their binding to mitochondrial anionic cardiolipin (Figure 22), thereby interrupting the anchoring of cytochrome *c* (and cytochrome *c* oxidase, Complex IV) at the inner mitochondrial membrane, inducing cytochrome *c* release into the cytosol with triggering of mitochondrial apoptosis (intrinsic pathway) [30].

In overall conclusion, compound **1** and compound **3** both trigger the mitochondrial pathway of apoptosis [30] after transcriptional (re)activation of the p53 protein. Compound **2** is not able to trigger mitochondrial apoptosis; instead, it represents a colchic(in)oid mitotic poison locking cells in anaphase of mitosis, probably binding to the well-known colchicine-binding site at β-tubulin [113,114,115,116,117]. There is no hint from my data that compound **3** can also occupy the colchic(in)oid-binding site at β-tubulin; probably, the molecule is too large for that binding site. Therefore, compound **2** only stops tumor cell growth to ±0% growth index, but the cancer cells are all present still. In contrast, compound **1** and compound **3** both completely destroy the tumor cells to nearly −100% of their growth index by ‘genetically programmed suicide’.

Importantly, compound **1** and compound **3** are tumoricidal versus both wild-type p53 protein-expressing tumor cell lines and mutant p53 protein-expressing or none p53-expressing cancer cell lines (Table S1). This pointed to an additional p53-independent induction of mitochondrial apoptosis by compound **1** and compound **3** through their binding to mitochondrial cardiolipin based on ionic plus-minus charge attraction and hydrophobic interaction(s), thereby disrupting the membrane-anchoring of cytochrome *c* and cytochrome *c* oxidase enzymes in the inner mitochondrial membrane leading to cytochrome *c* release into the cytosol. Compound **1** and compound **3** can be classified as essentially new antineoplastic drugs exploiting the cancer’s *Warburg* effect [6,7,8,9,10,11,12]. Cancer cell mitochondria exhibit abnormalities in their cardiolipin content, composition, and metabolism [9,10,21,22,23,24]. Therefore, cancer cell mitochondria bear a weakened interaction of cardiolipin (Figure 22) with cytochrome *c* and cytochrome *c* oxidase enzymes at the inner mitochondrial membrane. This fact is exploited by the polyammonium compounds **1** and **3** through binding to cancer cell’s anionic cardiolipin, terminally interrupting its weakened interactions with respiratory chain enzymes preceding mitochondrial apoptosis [25,26,27,28,29,30,31].

## 4. Experimental Section

### 4.1. Materials and Methods

#### 4.1.1. Chemicals

1-Adamantanammonium chloride (INN: amantadine hydrochloride) (1-aminoadamantane hydrochloride) [Lot: S4247215; *w* (*m*/*m*) = 100.0% (argentometric titration), mp > 360 °C (dec.)] and absolute ethanol *pro analysi* EMPLURA^®^ [Lot: K48011060; *w* (*n*/*n*) = 99.9% (gas chromatography, area%), *w* (H_2_O) (*m*/*m*) = 0.06%, non-volatile matter < 0.0001%] were purchased from Merck KGaA–EMD Millipore Corp. (Darmstadt, Germany). Sodium hydroxide (NaOH) pearls pure (Ph.Eur., BP, Food Grade) [Lot: 2J002792; *w* (*m*/*m*) = 99.31% (titration), sodium carbonate < 0.5%, SiO_2_ < 0.001%, NaCl < 0.008%, Na_2_SO_4_ < 0.0025%, As < 0.00001%, heavy metals (Cu, Fe, Mn, Ni, Pb, Hg) < 0.001%], 10.27 M [32% (*m*/*m*)] aqueous hydrochloric acid *pro analysi* [Lot: 3A001639; *w* (*m*/*m*) = 33.09% (titration), bromide < 0.005%, phosphate < 0.00005%, sulfate < 0.0001%, As < 0.000001%, Fe < 0.00002%, heavy metals (Ni, Pb, Zn) < 0.000005%], sodium hydrogen carbonate (sodium bicarbonate) *pro analysi* NaHCO_3_ [Lot: 4W000829; *w* (*m*/*m*) = 100.42% (titration), pH (5% in H_2_O) 8.04 (20 °C), chloride ≤ 0.001%, sulfate ≤ 0.005%, phosphate ≤ 0.005%, cations (K, Mg, Ca) ≤ 0.005%, As ≤ 0.0001%, heavy metals (Cu, Fe, Pb) ≤ 0.0005%], and (–)-colchicine sesquihydrate (×1½ H_2_O) = colchicine *BioChemica* [*w* (*m*/*m*) ≥ 98% (HPLC), specific rotation (20 °C, 589 nm) –240° to –250° (*c* = 1 in EtOH), *w* (H_2_O) (*m*/*m*) ≤ 3% (Karl Fischer titration)] were purchased from AppliChem (Darmstadt, Germany). Acetone (USP, BP, Ph.Eur.) Pharma Quality [Lot: 0000869897; *w* (*n*/*n*) = 99.9% (gas chromatography, area%), *w* (H_2_O) (*m*/*m*) = 0.3%, non-volatile matter 0.0002%, methanol < 0.05%, propan-2-ol < 0.05%, benzene < 0.0002%, ethanol < 500 ppm, heavy metals (Fe, Zn) < 1300 ppm, heavy metals (Cu, Mn) < 250 ppm], ethyl acetate *pro analysi* [Lot: 0000518022; *w* (*n*/*n*) = 99.9% (gas chromatography, area%), *w* (H_2_O) (*m*/*m*) = 0.01% (Karl Fischer titration), ethanol < 0.1%, methanol < 0.02%, methyl acetate < 0.02%, trace elements (Cr, Fe, Ni, Pb, Zn, P, S, K, Mg) < 0.00001%, Si < 0.00002%, Na < 0.0002%, non-volatile matter < 0.001%, acidity/alkalinity < 0.0005 meq/g] were purchased from PanReac AppliChem GmbH (Darmstadt, Germany). 1,3-Bis(chloromethyl)benzene (α,α′-dichloro-*m*-xylene) *purum* ≥ 98% (GC) [Lot: 385191/1; *w* (*m*/*m*) = 100.6% (argentometric titration after oxygen combustion), *w* (*n*/*n*) = 99.9% (gas chromatography, area %), mp 33.2–34.0 °C] was purchased from Fluka Chemie AG (Buchs, Switzerland). Thiosemicarbazide *puriss. p.a.* [Lot: 1167177V (Fluka); *w* (*m*/*m*) = 100.1% (iodometric titration), mp 181 °C (dec.), residue on ignition < 0.05%, metal trace analysis (inductively coupled plasma mass spectrometry) ≤ 50–5 mg/kg] was purchased from Sigma-Aldrich Corp. (St. Louis, MO, USA).

#### 4.1.2. Cell Lines

The National Cancer Institute (NCI) Developmental Therapeutics Program (DTP) 60 cancer cell lines (see Figure 18, Figure 19 and Figure 20, Table S1) were kindly provided and used by NCI [part of the National Institutes of Health (NIH), Bethesda, MD, USA]. The metastatic prostate cancer cell lines PC-3 (CRL-1435™) (bone metastasis, adenocarcinoma) and DU-145 (HTB-81™) (brain metastasis, adenocarcinoma) were retrieved from American Type Culture Collection (ATCC^®^, Manassas, VA, USA).

#### 4.1.3. Analytical Methods

The *Fourier*-transform infrared (FT–IR) spectra were recorded with neat substance on JASCO FT/IR–4100 type A and FT/IR–6100 type A spectrometers (JASCO International Co. Ltd., Tokyo, Japan). Given FT–IR absorbance bands, expressed in wavenumbers (cm^−1^), are characterized in intensity as strong (str), middle (m), weak (w), and broad (br). The proton nuclear magnetic resonance (^1^H-NMR), carbon-13 nuclear magnetic resonance (^13^C-NMR), and ^13^C-Distortionless Enhancement by Polarization Transfer Including Detection of Quaternary Nuclei (DEPTQ ^13^C-NMR) [44,45] NMR spectroscopy experiments were recorded at a temperature of 25 °C using a Bruker Avance 700 III HD nuclear magnetic resonance spectrometer (Bruker BioSpin GmbH, Rheinstetten, Germany) [^1^H-NMR (700.43 MHz), ^13^C-NMR (176.12 MHz)]. The spectra were referenced to the center of the NMR solvent signal [^1^H-NMR: *δ* 2.51 (DMSO-*d*_6_); ^13^C-NMR: *δ* 39.41 (DMSO-*d*_6_)]. Additional ^1^H-NMR, ^13^C-NMR, DEPTQ ^13^C-NMR, gradient-selected Correlation Spectroscopy (gs-COSY) [45], gradient-selected Heteronuclear Multiple Quantum Coherence (gs-HMQC) [45] and gradient-selected Heteronuclear Multiple Bond Correlation (gs-HMBC) [45] spectra were recorded using a Bruker Avance III HD 400 nuclear magnetic resonance spectrometer (Bruker BioSpin GmbH, Rheinstetten, Germany) [^1^H-NMR (400.13 MHz), ^13^C-NMR (100.62 MHz)] at a temperature of 300.0 K. The spectra were referenced to the center of the NMR solvent signal [^1^H-NMR: *δ* 2.50 (DMSO-*d*_6_); ^13^C-NMR: *δ* 39.52 (DMSO-*d*_6_)]. Given chemical shifts *δ* [from tetramethylsilane (TMS): *δ* = 0] are specified as singlet (s), broad singlet (br s), doublet (d), triplet (t), quartet (q), multiplet (m), and broad multiplet (br m). Electrospray ionization time-of-flight mass spectrometry (ESI-ToF-MS) of compound **3** was conducted in positive ion mode on an Agilent LC-ESI-ToF instrument using an Agilent 1260 Infinity II liquid chromatography (LC) stack with an Agilent ZORBAX 300SB-C8 (4.6 mm ID × 50 mm) column (pore size 300 Å, particle size 5 µm) coupled to an Agilent 6230B time-of-flight LC/MS (LC/ToF) system (Agilent Technologies, Inc., Santa Clara, CA, USA). Source settings were *ϑ* (dry gas) = 350 °C, gas flow 10 L/min, nebulizer pressure *p*_n_ = 35 psi (2.4132 bar). The capillary voltage was set to *V*_c_ = 3500 V, and fragmentor voltage was set to *V*_f_ = 175 V. The scan range was *m*/*z* 105–3000. Elemental analyses (C, H, N, S, O) were conducted on the EURO EA3000 CHNS–O elemental analyzer (EuroVector SpA, Milan, Italy) by HEKAtech GmbH (Wegberg, Germany).

#### 4.1.4. Software

Crystal data visualization and molecular modeling were performed with ACD/Chem Sketch version 2022.1.0 with integrated ACD/3D Viewer (Advanced Chemistry Development, Inc., Toronto, ON, Canada) and processed with Mercury 2022.3.0 [The Cambridge Crystallographic Data Centre (CCDC), Cambridge, UK]. Additional molecular modeling was performed with ACD/Chem Sketch version 12.01 with integrated ACD/3D Viewer (Advanced Chemistry Development, Inc., Toronto, ON, Canada) and processed with Mercury 3.1 version 3.1.1 [The Cambridge Crystallographic Data Centre (CCDC), Cambridge, UK]. The mass spectrometric *m*/*z* values were calculated with the Scripps Research Core Service Molar Mass Calculator (The Scripps Research Institute, Center for Metabolomics and Mass Spectrometry, San Diego, CA, USA).

### 4.2. Biological Testing

#### 4.2.1. National Cancer Institute (NCI) Developmental Therapeutics Program (DTP) 60-Cancer Cell 5-Dose Testing

Compounds that exhibit significant growth inhibition in the One-Dose Screen are evaluated against the 60-cell panel at five concentration levels [119]. The human tumor cell lines of the cancer screening panel are grown in RPMI-1640 medium containing 5% fetal bovine serum (FBS) and 2 mM L-glutamine. For a typical screening experiment, cells are inoculated into 96-well microtiter plates in 100 μL at plating densities ranging from 5000 to 40,000 cells/well, depending on the doubling time of individual cell lines. After cell inoculation, the microtiter plates are incubated at 37 °C, 5% CO_2_, 95% air, and 100% relative humidity for 24 h prior to the addition of experimental drugs. After 24 h, two plates of each cell line are fixed in situ with trichloroacetic acid (TCA) to represent a measurement of the cell population for each cell line at the time of drug addition (*T*_z_). Experimental drugs are solubilized in dimethyl sulfoxide (DMSO) at 400-fold the desired final maximum test concentration and stored frozen prior to use. At the time of drug addition, an aliquot of frozen concentrate is thawed and diluted to twice the desired final maximum test concentration with a complete medium containing 50 μg/mL gentamicin. Additional four, 10-fold or ½ log serial dilutions are made to provide a total of five drug concentrations plus control. Aliquots of 100 μL of these different drug dilutions are added to the appropriate microtiter wells already containing 100 μL of the medium, resulting in the required final drug concentrations.

Following drug addition, the plates are incubated for an additional 48 h at 37 °C, 5% CO_2_, 95% air, and 100% relative humidity. For adherent cells, the assay is terminated by the addition of cold TCA. Cells are fixed in situ by the gentle addition of 50 μL of cold 50% (*m*/*v*) TCA (final concentration, 10% TCA) and incubated for 60 min at 4 °C. The supernatant is discarded, and the plates are washed five times with tap water and air dried. Sulforhodamine B (SRB) solution [100 μL at 0.4% (*m*/*v*) in 1% acetic acid] is added to each well, and plates are incubated for 10 min at room temperature. After staining, unbound dye is removed by washing five times with 1% acetic acid, and the plates are air dried. Bound stain is subsequently solubilized with 10 mM trizma^®^ base [2-amino-2-(hydroxymethyl)-1,3-propandiol, tris base, trometamol], and the absorbance is read on an automated plate reader at a wavelength of *λ* = 515 nm. For suspension cells, the methodology is the same, except that the assay is terminated by fixing settled cells at the bottom of the wells by gently adding 50 μL of 80% TCA (final concentration, 16% TCA). Using the seven absorbance measurements [time zero, (*T*_z_), control growth, (*C*), and test growth in the presence of the drug at the five concentration levels (*T*_i_)], the percentage growth is calculated at each of the drug concentration levels. Percentage growth inhibition is calculated as:

[(*T*_i_ − *T*_z_)/(*C* − *T*_z_)] × 100 for concentrations for which *T*_i_ ≥ *T*_z_

[(*T*_i_ − *T*_z_)/*T*_z_] × 100 for concentrations for which *T*_i_ < *T*_z_

Three dose-response parameters are calculated for each experimental agent. Growth inhibition of 50% (**GI50**) is calculated from [(*T*_i_ − *T*_z_)/(*C* − *T*_z_)] × 100 = 50, which is the drug concentration resulting in a 50% reduction in the net protein increase (as measured using SRB staining) in control cells during the drug incubation. The drug concentration resulting in total growth inhibition (**TGI**) is calculated from *T*_i_ = *T*_z_. The **LC50** (concentration of drug resulting in a 50% reduction in the measured protein at the end of the drug treatment as compared to that at the beginning) indicating a net loss of cells following treatment is calculated from [(*T*_i_ − *T*_z_)/*T*_z_] × 100 = −50. Values are calculated for each of these three parameters if the level of activity is reached; however, if the effect is not reached or is exceeded, the value for that parameter is expressed as greater or less than the maximum or minimum concentration tested.

An outline for reading the NCI 60 Cell Five-Dose Screen data was given in Section 2.4.1. The numerical screening data are given in Supplementary Information (Table S1).

#### 4.2.2. Cytochrome *c* Assay (Mitochondrial and Cytosolic)

Cell numbers of 2 × 10^6^ PC-3 and DU-145 cells were seeded into a 100-well plate. The next day, cells were treated with either dimethyl sulfoxide (DMSO) or six concentrations of compounds **1**, **2**, and **3** (500 nM, 1 µM, 5 µM, 25 µM, 50 µM, 75 µM) dissolved in DMSO for a time of 48 h. The concentrations of 25 µM, 50 µM, and 75 µM of compounds **1**, **2**, and **3** were found to be highly toxic for both cell lines 48 h post-treatment. The enzyme-linked immunosorbent assay (ELISA) for cytochrome *c* was performed according to the manufacturer’s instructions [Cytochrome c (human), ELISA kit (Enzo Life Sciences, Inc., Farmingdale, NY, USA)] with two fractions (mitochondrial and cytosolic) of each cell line at the concentrations of compound **1**, **2**, and **3** of 500 nM, 1 µM, 5 µM, and 25 µM. The protein content for each fraction was calculated to determine the mass content of cytochrome *c* in each fraction (pg cytochrome *c*/mg total protein). The mass content of cytochrome *c* (pg cytochrome *c*/mg total protein) was normalized towards the corresponding blank DMSO control and is given in percent (%) of blank DMSO control.

#### 4.2.3. Cytotoxicity and HIV-1_LAI_ Replication Reverse Transcriptase Assays with Compound **1**

The cytotoxicity and human immunodeficiency virus type 1 (HIV-1) strain LAI replication assays were performed in freshly explanted primary human peripheral blood mononuclear cells (PBM cells) according to published procedures [36]. The assays were conducted at least in triplicate and treated statistically (if possible). HIV-1_LAI_ (= HIV-1_BRU_ = LAV-1) was assayed in primary human peripheral blood lymphocyte (PBL) cells in the presence of a drug being evaluated. The parameter for antiviral activity was a reduction in reverse transcriptase (RT) activity in the cell supernatant after Triton X–100-mediated lysis of released virions, as measured using [5α-^3^H]dTTP (5α-tritiated thymidine 5′-triphosphate) incorporation into poly(rA)•poly(dT) directed by the primed RNA template poly(rA)•oligo(dT). It should be noted that the assay did not detect RT inhibition by potential RT inhibitors *per se* but indirectly quantified the amount of released HIV-1 in the supernatant. The detailed assay methodology was reported by *Schinazi* et al. [120] as based on an older assay system of *Spira* et al. [121]. The experiments were conducted in triplicate and treated statistically using regression curve analysis (*r*^2^ coefficient of determination). The RT inhibitor AZT (zidovudine, 3′-azido-3′-deoxythymidine; RETROVIR™) served as a positive control. Cytotoxicity on PBL and the other cells (CCRF−CEM, Vero) exerted by the test compounds was determined as described by *Stuyver* et al. [122] by application of the CellTiter 96^®^ AQ_ueous_ One Solution Cell Proliferation Assay (Promega Corp., Madison, WI, USA). Briefly, the phenazine ethosulfate (PES)-coupled reduction of the tetrazolium salt 3-(4,5-dimethylthiazol-2-yl)-5-(3-carboxymethoxyphenyl)-2-(4-sulfophenyl)-2*H*-tetrazolium (MTS) to a purple, water-soluble formazan by living, undamaged cells was measured.

### 4.3. X-ray Crystallographic Determination of the Crystal and Molecular Structure of (M)-10-(2-Carbamothioylhydrazinyl)-10-demethoxycolchicine Sesquihydrate × ½ (Ethyl Acetate) = N-[(aS,7S)-10-(2-Carbamothioylhydrazinyl)-1,2,3-trimethoxy-9-oxo-5,6,7,9-tetrahydrobenzo[a]heptalen-7-yl]acetamide Sesquihydrate × ½ (Ethyl Acetate) (Crystalline Compound ***2***)

#### 4.3.1. Crystallization of Compound **2** Single Crystals

Compound **2** was crystallized by atmospheric evaporation from ethyl acetate (EtOAc) overnight (time ~10 h) in an open *Petri* dish at room temperature (RT, *ϑ* = 14.0 °C). A suitable single crystal was selected and isolated under polarized light microscopic examination.

#### 4.3.2. Crystal Data of Compound **2** Single Crystals

The crystal data of compound **2** were collected on a Bruker X8 *APEX*-II diffractometer with a CCD area detector and multi-layer mirror monochromated Mo_Kα_ radiation. The structure was solved using direct methods, refined with the *SHELX* software package (*SHELXS97*, *SHELXL97*) [123], and expanded using *Fourier* techniques. All non-hydrogen atoms were refined anisotropically. Hydrogen atoms were assigned to idealized positions and were included in structure factor calculations. The *Flack* parameter *x_Flack_* [124] was near zero, indicating that the right absolute configuration was solved.

#### 4.3.3. Deposition of the X-ray Crystallographic Structure Determination of Compound **2**

Crystallographic data have been deposited with The Cambridge Crystallographic Data Centre (CCDC), 12 Union Road, Cambridge, CB2 1EZ, United Kingdom, as supplementary publication № **CCDC 1,839,505** (ID: **RIVGOW**). These data can be obtained free of charge from CCDC via https://www.ccdc.cam.ac.uk/structures/ (accessed on 16 February 2024).

### 4.4. Chemical Synthesis

#### 4.4.1. Salt-Containing Tetrakis{3-[(tricyclo[3.3.1.1^3,7^]decan-1-ammonio)methyl]benzyl}ammonium Pentachloride (Compound **1** × 1.5 NaCl, **PT162** × 1.5 NaCl)

Masses of 2.50 g 1-adamantanammonium chloride (1-aminoadamantane hydrochloride) (*M* = 187.71 g/mol, 13.32 mmol) and 3.50 g 1,3-bis(chloromethyl)benzene (*M* = 175.06 g/mol, *m*-xylylene dichloride, α,α′-dichloro-*m*-xylene) (19.99 mmol) were dissolved in 30 mL of 90% (*v*/*v*) aqueous ethanol. A solution of 1.60 g sodium hydroxide (NaOH) (40.00 mmol) in 40 mL of water was added, and the suspension was refluxed for 3 h. After 20 min and 40 min of reflux, 20 mL of acetone was added at each time using the reflux condenser. After 60 min, 80 min, and 100 min reflux, 40 mL of acetone was added at each time in the same way. After 120 min reflux, an additional 20 mL of acetone was added in the same way. Then, after 10 min cooling at +0–2 °C, the colorless suspension was warm filtrated through filter paper. The filtrate (pH 9) was mixed with 100 mL of water and evaporated in vacuo from the acetone to a volume of ca. 170 mL. Afterwards, 2 mL of 10.27 M [32% (*m*/*v*)] hydrochloric acid (20.54 mmol) was added, and the colorless solution (pH 2–3) was evaporated in vacuo to a volume of ca. 120 mL.

The now turbid suspension was mixed with 20 mL of water and was extracted with 100 mL of ethyl acetate (EtOAc) to remove residual 1,3-bis(chloromethyl)benzene. The aqueous phase was isolated and evaporated in vacuo to a volume of ca. 50 mL. This aqueous phase was then frozen at −25 °C for 1½ h. The crystalline white precipitate of crude compound **1** × 1.5 NaCl (**PT162** × 1.5 NaCl) was filtered and dried over CaCl_2_ in vacuo (from the filtrate additional substance could be obtained, which was combined with the main yield). From the separated EtOAc phase by cooling at +0–2 °C for 3 h, additional crude compound **1** × 1.5 NaCl (**PT162** × 1.5 NaCl) could be isolated, which was combined with the main fraction.

The combined crude product (730 mg) was dissolved in 50 mL of 80% (*v*/*v*) aqueous acetone by short (5 min) reflux and was hot filtrated through filter paper to remove a few cloudy impurities. The filtrate was transferred and mixed with 40 mL of 50% (*v*/*v*) aqueous acetone. Afterward, the filtrate was evaporated in vacuo from the acetone. The resulting solution (pH 5) was acidified to pH 0–1 by the addition of 0.6 mL of 10.27 M [32% (*m*/*v*)] hydrochloric acid (6.16 mmol). Immediately, a fine white crystalline precipitate formed. The suspension was mixed with 10 mL of 50% (*v*/*v*) aqueous acetone and was frozen at −25 °C for 2½ h. The fine white crystals (small needles) of compound **1** × 1.5 NaCl (**PT162** × 1.5 NaCl) were filtered and dried over CaCl_2_ in vacuo.
Compound:Compound **1** × 1.5 NaCl (**PT162** × 1.5 NaCl)Molecular formula: C_72_H_100_N_5_Cl_5_ × 1.5 NaClMolecular weight: 1300.54 g/molYield: 563 mg (13%)Elemental analysis:calculated:C 66.49% H 7.75% N 5.38% O 0.00%found:C 66.47% H 7.54% N 4.25% O 0.82%C 66.51% H 7.53% N 4.24% O 0.80%FT–IR (cm^−1^): 2925, 2850, 2760, 2710, 2436, 1610, 1585, 1494, 1459, 1269, 1108, 1074, 1011, 973, 794, 777, 762, 731, 693^1^H-NMR: (DMSO-*d*_6_, ppm)1.62 (3 H, d; ^2^*J*_gem_ = –12.1 Hz; δ-CH*_axial_*), 1.69 (3 H, d; ^2^*J*_gem_ = –12.4 Hz; δ-CH*_equatorial_*), 2.02 (6 H, s; β-CH_2_), 2.15 (3 H, s; γ-CH), 4.10 (2 H, t; ^3^*J*_vicinal_ = 6.3 Hz; 8-CH_2_), 4.78 (2 H, s; 7-CH_2_), 7.42–7.49 (2 H, m; H-4, H-6), 7.65 (1 H, d; ^3^*J*_ortho_ = 7.1 Hz; H-5), 7.69 (1 H, s; H-2), 9.24 (2 H, br s; 8-NH_2_^+^)^13^C-NMR: (DMSO-*d*_6_, ppm)28.50 (γ-CH), 35.25 (δ-CH_2_), 37.35 (β-CH_2_), 42.31 (8-CH_2_), 45.84 (7-CH_2_), 57.06 (α-C), 128.87 (C-4) *, 129.14 (C-6) *, 130.36 (C-2) *, 130.62 (C-5) *, 133.22 (C-3), 137.84 (C-1)* These assignments are tentative and interchangeable (they could not be assigned unequivocally to the individual carbons).

#### 4.4.2. Pure (Salt-Free) Tetrakis{3-[(tricyclo[3.3.1.1^3,7^]decan-1-ammonio)methyl]benzyl}ammonium Pentachloride (Compound **1**, **PT162**, **NSC 796018**)

1-Adamantanammonium chloride (1-aminoadamantane hydrochloride) (*M* = 187.71 g/mol, 10.075 g, 53.6732 mmol) was dissolved in water (100 mL). A solution of sodium hydroxide (NaOH) (2.160 g, 54.0000 mmol) in water (20 mL) was added. Residues were transferred with water (20 mL). A white, amorphous precipitate of the free base 1-aminoadamantane formed immediately. The suspension was frozen at −25 °C for 1 h. The heavy white precipitate of 1-aminoadamantane (free base) was filtered and sucked dry on the filter (ca. 1 h).

The still wet 1-aminoadamantane (free base) and 1,3-bis(chloromethyl)benzene (*m*-xylylene dichloride, α,α′-dichloro-*m*-xylene) (*M* = 175.06 g/mol, 7.498 g, 42.8310 mmol) were suspended in absolute ethanol (200 mL). The suspension was refluxed for 3 h. After 40 min reflux, a clear colorless solution formed in the heat. After short (5 min) cooling at +0–2 °C, the colorless suspension was hot filtrated through one layer of filter paper. Residues were transferred and rinsed with absolute ethanol (10 mL) and acetone (30 mL). The filtrate was mixed with acetone (300 mL), 10.27 M [32% (*m*/*v*)] hydrochloric acid (3200 µL 32.8640 mmol), and ethyl acetate (EtOAc) (200 mL). The mixture was frozen at –25 °C for 2.5 h. Then water (200 mL) and EtOAc (1000 mL) were added, and the mixture was shaken vigorously for 1 min and was frozen at –25 °C for 2.5 h. Afterward, 10.27 M [32% (*m*/*v*)] hydrochloric acid (3000 µL 30.8100 mmol) was added, and the mixture was shaken vigorously for 1 min and was additionally frozen at –25 °C for 30 min. The upper phase was then decanted, and the lower aqueous phase was isolated. The isolated upper EtOAc phase was extracted with water (90 mL), and the aqueous phase was isolated after phase separation and combined with the first aqueous phase. Finally, the isolated upper EtOAc phase was re-extracted with water (100 mL), which was acidified with 3000 µL 10.27 M [32% (*m*/*v*)] hydrochloric acid (30.8100 mmol). The aqueous phase was isolated after phase separation and was combined with the two prior aqueous phases. The combined aqueous phases (*V* = 500 mL) were evaporated in vacuo at the lowest possible temperature to a volume of ca. 200 mL until heavy crystallization started. The crystallizing suspension was then cooled at +0–2 °C for 6 h and frozen at −25 °C for 20 min to complete crystallization. The evolved first yield (1.543 g) of the white, fine needles was filtered and dried over CaCl_2_ in vacuo. The filtrate was additionally cooled at +0–2 °C for 50 h. The evolved second yield (62 mg) of the white, fine needles was filtered and dried over CaCl_2_ in vacuo.
Compound:Compound **1** (**PT162**, **NSC 796018**)Molecular formula: C_72_H_100_Cl_5_N_5_Molecular weight: 1212.86 g/molYield: 1.605 g (12.4%)Elemental analysis:calculated:C 71.30% H 8.31% N 5.77% O 0.000%found:C 65.67% H 7.76% N 4.29% O 0.669%C 65.66% H 7.74% N 4.27% O 0.691%FT–IR (cm^−1^): 2925, 2850, 2760, 2710, 2436, 1610, 1585, 1494, 1459, 1269, 1108, 1074, 1011, 973, 794, 777, 762, 731, 693^1^H-NMR: (DMSO-*d*_6_, ppm)1.61 (3 H, d; ^2^*J*_gem_ = –11.7 Hz; δ-CH*_axial_*), 1.68 (3 H, d; ^2^*J*_gem_ = –11.7 Hz; δ-CH*_equatorial_*), 2.00 (6 H, s; β-CH_2_), 2.14 (3 H, s; γ-CH), 4.09 (2 H, t; ^3^*J*_vicinal_ = 6.4 Hz; 8-CH_2_), 4.77 (2 H, s; 7-CH_2_), 7.43–7.48 (2 H, m; H-4, H-6), 7.64 (1 H, d; ^3^*J*_ortho_ = 7.1 Hz; H-5), 7.68 (1 H, s; H-2), 9.24 (2 H, br s; 8-NH_2_^+^ ammonium)^13^C-NMR: (DMSO-*d*_6_, ppm)28.50 (γ-CH), 35.25 (δ-CH_2_), 37.35 (β-CH_2_), 42.31 (8-CH_2_), 45.84 (7-CH_2_), 57.06 (α-C), 128.87 (C-4) *, 129.14 (C-6) *, 130.36 (C-2) *, 130.62 (C-5) *, 133.22 (C-3), 137.84 (C-1)* These assignments are tentative and interchangeable (they could not be assigned unequivocally to the individual carbons).

#### 4.4.3. (*M*)-10-(2-Carbamothioylhydrazinyl)-10-demethoxycolchicine Monohydrate × ⅔ (Ethyl Acetate) = *N*-[(a*S*,7*S*)-10-(2-Carbamothioylhydrazinyl)-1,2,3-trimethoxy-9-oxo-5,6,7,9-tetrahydrobenzo[*a*]heptalen-7-yl]acetamide Monohydrate × ⅔ (Ethyl Acetate) (Compound **2**, **PT166**, **NSC 750423**)

Masses of 5.00 g (–)-colchicine sesquihydrate (× 1½ H_2_O) (*M* = 426.46 g/mol, 11.72 mmol) and 1.08 g thiosemicarbazide (*M* = 91.13 g/mol, 11.85 mmol) were dissolved in 25 mL of 90% (*v*/*v*) aqueous ethanol by refluxing (5 min). Then, a solution of 0.48 g sodium hydroxide (12.00 mmol) in 2 mL of water was added, and the deep orange-red solution was refluxed for 5 min. The cold, deep orange-red solution, after cooling at −25 °C for 20 min, was titrated by dropwise addition of 1.1 mL of 10.27 M [32% (*m*/*v*)] hydrochloric acid (11.30 mmol), which was diluted with 2 mL of water. Successively, the volume of the solution was reduced in vacuo by one-half to a volume of 15 mL. The reddish-brown solution was mixed with 100 mL of water and was titrated with 1.1 mL of 10.27 M [32% (*m*/*v*)] hydrochloric acid (11.30 mmol), which was diluted with 2 mL of water. The oily emulsion was extracted with 50 mL of ethyl acetate (EtOAc) in a separation funnel. The separated aqueous phase (pH 2) was additionally extracted with 40 mL of EtOAc. After neutralization of this aqueous phase with sodium hydrogen carbonate NaHCO_3_, the aqueous phase (pH 7–8) was extracted twice with 40 mL of EtOAc each. The EtOAc phases were combined and washed twice with 100 mL of water each. The washed EtOAc phase, which already precipitated, was mixed with 50 mL of acetone and was frozen at −25 °C for 10 h. If precipitation did not start spontaneously, the volume of the solution was reduced in vacuo until coagulation started. The evolved bright yellow, fine crystalline precipitate of compound **2** was filtered (1.01 g) and dried over CaCl_2_ in vacuo. From the combined aqueous phases by cooling in the refrigerator (+0–2 °C, 2 days), a second yield of compound **2** could be obtained (1.75 g). It was combined with the main yield.
Compound:Compound **2** (**PT166**, **NSC 750423**)Molecular formula: C_22_H_26_N_4_O_5_S × H_2_O × ⅔ (C_4_H_8_O_2_)Molecular weight: 535.28 g/molYield: 2.76 g (44%)Elemental analysis:calculated:C 55.35% H 6.28% N 10.47% S 5.99%found:C 55.34% H 6.29% N 10.35% S 6.00%C 55.38% H 6.14% N 10.34% S 6.00%FT–IR (cm^−1^): 3421, 3249, 2934, 1727, 1703, 1660, 1601, 1543, 1488, 1449, 1432, 1402, 1375, 1350, 1322, 1282, 1241, 1193, 1142, 1091, 1042, 917, 899, 863, 781^1^H-NMR: (DMSO-*d*_6_, ppm)1.18 (1.5 H, t; ^3^*J* = 7.1 Hz; O–CH_2_–C*H*_3_ ethyl acetate), 1.85 (1 H, m; H_A_-6), 1.86 (3 H, s; 17-CH_3_), 1.99 (1.5 H, s; ROOC–C*H*_3_ ethyl acetate), 2.05 (1 H, m; H_B_-6), 2.19 (1 H, m; H_A_-5), 2.57 (1 H, m; H_B_-5), 3.51 (3 H, s; 13-OCH_3_) *, 3.79 (3 H, s; 15-OCH_3_) *, 3.83 (3 H, s; 14-OCH_3_) *, 4.03 (1 H, q; ^3^*J* = 7.1 Hz; O–C*H*_2_–CH_3_ ethyl acetate), 4.37 (1 H, m; H-7), 6.60 (1 H, d; ^3^*J* = 11.1 Hz; H-11), 6.76 (1 H, s; H-4), 7.14 (1 H, s; H-8), 7.20 (1 H, d; ^3^*J* = 10.9 Hz; H-12), 7.56 (1 H, br s; H_2_N–C=S amino, 4′-H_A_), 7.96 (1 H, br s; H_2_N–C=S amino, 4′-H_B_), 8.56 (1 H, d; ^3^*J* = 7.6 Hz; N–H acetamide), 9.06 (1 H, s; 1′-N–H), 9.59 (1 H, s; 2′-N–H)^13^C-NMR: (DMSO-*d*_6_, ppm)14.05 (O–CH_2_–C*H*_3_ ethyl acetate), 20.72 (ROOC–C*H*_3_ ethyl acetate), 22.49 (C-17, CH_3_ acetamide), 29.33 (C-5), 36.34 (C-6), 51.38 (C-7), 55.84 (14-OCH_3_) **, 59.72 (O–C*H*_2_–CH_3_ ethyl acetate), 60.62 (13-OCH_3_, 15-OCH_3_) **, 107.61 (C-4), 108.27 (C-11), 126.23 (C-8), 131.57 (C-1a), 134.26 (C-4a), 137.21 (C-12), 140.71 (C-3) ***, 150.34 (C-1) ***, 150.40 (C-10), 150.46 (C-12a), 152.61 (C-2) ***, 152.73 (C-7a), 168.39 (C-16, HN–C=O acetamide), 170.30 (C=O ester carbonyl, ethyl acetate), 174.81 (C-9, C=O carbonyl), 181.86 (C-3′, C=S thiocarbonyl)*, **, *** These assignments are tentative and interchangeable (they could not be assigned unequivocally to the individual protons or carbons, respectively).

#### 4.4.4. [(Bis{3-[(tricyclo[3.3.1.1^3,7^]decan-1-ylamino)methyl]benzyl}ammonio)bis(methanediylbenzene-3,1-diylmethanediyl)]di-2-[(a*S*,7*S*)-7-(acetylamino)-1,2,3-trimethoxy-9-oxo-5,6,7,9-tetrahydrobenzo[*a*]heptalen-10-yl]-*N*-(tricyclo[3.3.1.1^3,7^]decan-1-yl)hydrazinecarbothioamide Chloride Pentahydrate (Compound **3**, **PT167**, **NSC 799315**) (synthesized at Saturday, 27 May 2017)

Materials:

Compound **1** (**PT162**, **NSC 796018**) = tetrakis{3-[(tricyclo[3.3.1.1^3,7^]decan-1-ammonio)methyl]benzyl}ammonium pentachloride (C_72_H_100_Cl_5_N_5_) (*M* = 1212.86 g/mol) [*w* (*n*/*n*) ≥ 99% (^1^H-NMR and elemental analysis)], synthesized at Friday, 30 December 2016.

Compound **2** (**PT166**, **NSC 750423**) = *N*-[(a*S*,7*S*)-10-(2-carbamothioylhydrazinyl)-1,2,3-trimethoxy-9-oxo-5,6,7,9-tetrahydrobenzo[*a*]heptalen-7-yl]acetamide monohydrate × ⅔ (ethyl acetate) [C_22_H_26_N_4_O_5_S × H_2_O × ⅔ (C_4_H_8_O_2_)] (*M* = 535.28 g/mol) [*w* (*n*/*n*) ≥ 98% (^1^H-NMR and elemental analysis)], synthesized at Thursday, 29 January 2009.

Compound **1** [**PT162** (**NSC 796018**)] (*M* = 1212.86 g/mol, 300 mg, 247.3492 µmol) and compound **2** [**PT166** (**NSC 750423**)] (*M* = 535.28 g/mol, 300 mg, 560.4543 µmol) were suspended in absolute ethanol (20 mL) and the yellow suspension was gently (do not overheat!) heated to 40–50 °C for 4 min by use of a heat gun. Then water (1000 µL) was added under stirring, and all solids dissolved to give a bright yellow solution. A solution of sodium hydroxide (37 mg, 925.0000 µmoL) in water (2000 µL) was added under stirring. Instantly, the color of the solution turned orange-yellow. After pre-cooling at +0–2 °C for 12 min, the mixture was frozen at −25 °C for 10 min. After adding water (10 mL), the precipitating suspension was frozen at −25 °C for 2 h. The evolved first yield (212 mg) of the lemon yellow, amorphous substance compound **3** was filtered, dried by vacuum suction for 30 min on the sintered glass filter, and dried over CaCl_2_ in vacuo. Successively, the filtrate was transferred with water (20 mL) and was frozen at −25 °C for 30 min. After cooling at +0–2 °C for 1 h, the evolved second yield (17 mg) of the lemon yellow, amorphous substance compound **3** was filtered, carefully dried by vacuum suction for 30 min on the sintered glass filter, and dried over CaCl_2_ in vacuo. Both yields were combined.
Compound:Compound **3** (**PT167**, **NSC 799315**)Molecular formula: C_116_H_142_ClN_11_O_10_S_2_ × 5 H_2_OMolecular weight: 2040.10 g/molYield: 229 mg (45%)Elemental analysis:calculated:C 68.29% H 7.51% N 7.55% S 3.14% O 11.76%found:C 60.31% H 6.68% N 8.01% S 3.34% O 11.84%C 60.24% H 6.45% N 7.98% S 3.38% O 11.64%^1^H-NMR: (DMSO-*d*_6_, ppm)1.48–1.68 (24 H, br m; δ-CH_2_, adamantane), 1.83 (2 H, m; H_A_-6, colch), 1.85 (6 H, s; 17-CH_3_, colch), 2.01 (2 H, m; H_B_-6, colch), 2.17 (2 H, m; H_A_-5, colch), 2.52 (2 H, m; H_B_-5, colch), 3.48 (6 H, s; 13-OCH_3_, colch) *, 3.65 (2 H, br s; secondary amine N–H), 3.77 (6 H, s; 15-OCH_3_, colch) *, 3.82 (6 H, s; 14-OCH_3_, colch) *, 4.26 (br m; 8-CH_2_, *m*-xylylene), 4.32–4.39 (2 H, br m; H-7, colch), 4.76 (s; 7-CH_2_, *m*-xylylene), 6.73 (2 H, s; H-4, colch), 7.03 (2 H, br m; H-11, colch), 7.06 (2 H, s; H-8, colch), 7.12–7.72 (br m; H-4, H-6, H-5, H-2, *m*-xylylene), 7.17 (2 H, br m; H-12, colch), 8.54 (2 H, d; ^3^*J* = 7.7 Hz; N–H acetamide, colch), 9.59 (2 H, s; 2′-N–H, hydrazinecarbothioamide)colch = the colchic(in)oid part of compound **3**; * these assignments are tentative and interchangeable (they could not be assigned unequivocally to the individual methoxy groups); the adamantane resonances at *δ* 2.00 ppm (24 H; β-CH_2_) and *δ* 2.14 ppm (12 H; γ-CH), and 1′-N–H could not being detected due to paramagnetic resonance compression.

## 5. Patents

The three compounds **1**, **2,** and **3** are proprietary to PopTest Oncology LLC/Palisades Therapeutics (Cliffside Park, NJ, USA) and are filed in: Altschul, R.L.; Theise, N.D.; Kesel, A.J.; Rapkin, M.; O’Brien, R.; Arment, A.R. *Therapeutic Agents and Methods*; **PCT/WIPO Pat. Appl.** WO/2018/067520 A2, **2018** (filed 3 October 2017); **Eur. Pat. Appl.** EP 3 534 910 A2, **2019** (filed 3 October 2017); **U.S. Pat. Appl.** 2019/0381038 A1, **2019** (filed 3 October 2017); **U.S. Pat. Appl.** US 11,040,037 B2, **2021** (filed 3 October 2017); **U.S. Pat. Appl.** 2021/0353623 A1, **2021** (filed 28 April 2021); **U.S. Pat. Appl.** US 11,224,599 B2, **2022** (filed 28 April 2021); **U.S. Pat. Appl.** 2022/0298203 A1, **2022** (filed 22 November 2021); **U.S. Pat. Appl.** 11,702,443 B2, **2023** (filed 22 November 2021); **U.S. Pat. Appl.** 2023/0382945 A1, **2023** (filed 4 May 2023).

## Figures and Tables

**Table 1:** X-ray crystallographic data for compound **2**, deposited and published as *CSD Communication* № **CCDC 1,839,505** (ID: **RIVGOW**).

Empirical Formula	C_48_H_66_N_8_O_15_S_2_
Formula weight, *M*_r_ (g·mol^−1^)	1059.21
Temperature, *T* (K)	100(2)
Radiation, *λ* (Å)	Mo_Kα_ 0.71073
Crystal system	Monoclinic
Space group	*P*2_1_
Unit cell dimensions	
*a* (Å)	9.1886(5)
*b* (Å)	20.9047(10)
*c* (Å)	13.9841(7)
*α* (°)	90.00
*β* (°)	106.153(2)
*γ* (°)	90.00
Unit cell volume, *V* (Å^3^)	2580.1(2)
Formula units *per* unit cell, *Z*	2
Calculated density, *ρ*_calc_ (Mg·m^−3^)	1.363
Absorption coefficient, *μ* (mm^−1^)	0.178
*F*(000)	1124
Theta (*ϑ*) range for collection	1.80 to 26.05°
Reflections collected	49,598
Independent reflections	9686
Minimum/maximum transmission, *T*_min_/*T*_max_	0.9193/0.9929
Absorption correction	Multi-scan, *SADABS* 2008/1
Refinement method	Full-matrix least-squares on *F*^2^
Data/parameters/restraints	9686/693/5
Goodness-of-fit on *F*^2^, *S*	1.021
*Flack* parameter, *x*	0.09(6)
Final *R* indices [*I* > 2σ(*I*)]	*R*_1_ = 0.0506, *wR*^2^ = 0.1269
*R* indices (all data)	*R*_1_ = 0.0594, *wR*^2^ = 0.1325
Maximum/minimum residual electron density, Δ*ρ*_max_/Δ*ρ*_min_ (e·Å^−3^)	0.747/−0.496

**Table 2:** The liquid chromatographic (HPLC)/electrospray ionization (ESI) time-of-flight (ToF) mass spectrometric investigation of compound **3** after storing the substance over six years at +0–4 °C in the refrigerator. Given are the HPLC peak regions in the total ion current (TIC) chromatogram (Figure 15A) which were scanned in the ESI mass spectrometer with the observed fragmentation peaks (in parentheses: relative peak intensity of the 100% base peak), and the presumed ionization identities of the ionized species of compound **3** dissolved in acetonitrile with presence of 0.1% (*v*/*v*) HCOOH in the HPLC eluents.

HPLC Peak (min)	Peak Identity	ESI–MS Fragment Peaks (*m*/*z*) (Relative Peak Intensity of the 100% Base Peak)
7.842–7.925	ammonium trication	290.1670 (100%), 678.3662 (12.5%), 135.1157 (9.6%)
8.508–8.558	ammonium dication	712.3545 (100%), 123.0807 (16.5%), 356.6799 (2.1%), 483.2713 (1.3%)
8.608–8.658	ammonium monocation	712.3550 (100%), 543.3511 (27.9%), 356.6804 (18.1%), 123.0806 (14.2%), 653.3701 (5.4%), 483.2714 (2.5%), 409.2404 (1.7%)
8.691–8.741	ammonium monocation	712.3558 (100%), 356.6814 (19.1%), 578.2448 (6.3%), 135.1165 (5.8%)
9.024–9.074	ylide dication	483.2727 (100%), 965.5391 (80.9%), 712.3534 (19.5%), 123.0798 (14.1%), 356.6793 (5.0%), 831.4260 (4.3%), 577.2110 (3.4%), 213.1784 (2.3%)
9.740–9.973	ylide monocation	227.2000 (100%), 123.0804 (47.2%), 609.8644 (31.0%), 712.3522 (6.6%), 398.7700 (5.8%), 1218.7191 (5.0%), 796.5327 (4.0%), 483.2712 (2.5%)
13.386–13.453	neutral ylide	123.0805 (100%), 637.3055 (45.6%), 227.1988 (7.1%), 439.2038 (5.8%), 712.3521 (3.2%)

**Table 3:** Cytotoxicity and antiviral activity of tetrakis{3-[(tricyclo[3.3.1.1^3,7^]decan-1-ammonio)methyl]benzyl}ammonium pentachloride (compound **1**, **PT162**, **NSC 796018**) versus human immunodeficiency virus type 1 strain LAI (HIV-1_LAI_) in mammalian cells.

Drug	Cytotoxicity CC_50_ (µM) and CCRF–CEM Growth Inhibition at Fixed 1.0 µM Concentration (%, in Parentheses)			Anti-HIV-1_LAI_ Activity EC_50_ (µM)/EC_90_ (µM) in PBM Cells			
	PBM Cells	CCRF −CEM	Vero	EC_50_	EC_90_	SI_50_	*r* ^2^
Compound **1** (**PT162**, **NSC 796018**)	2.2	<1.0 (60.0)	1.8	0.56	4.3	3.9	0.93
AZT *	>100	14.3	56.0	0.0044 ± 0.0018	0.031 ± 0.015	>22,727	0.99

CC_50_, cytotoxic concentration 50%. EC_50_, effective inhibitory concentration 50%. EC_90_, effective inhibitory concentration 90%. SI_50_, selectivity index CC_50_/EC_50_. *r*^2^, coefficient of determination (*r*^2^ measure of goodness-of-fit) on EC_50_ and EC_90_. AZT, zidovudine (3′-azido-3′-deoxythymidine). * The given effective inhibitory concentrations (µM ± s.d.) for the positive control AZT were averaged and treated statistically from four (*n* = 4) independent determinations.

## Data Availability

Data are contained within the article and Supplementary Materials. Small (~10–20 mg) specimens of the compounds **1**, **2,** and **3** can be obtained from the author upon request.

## Supplementary Materials

The following supporting information can be downloaded at: https://www.mdpi.com/article/10.3390/molecules29040914/s1, ^1^H-NMR, ^13^C-NMR, FT–IR, DEPTQ ^13^C-NMR, gs-COSY, gs-HMQC and gs-HMBC for **1**, Figures S1–S7; FT-IR for **2**, Figure S8; one formula unit and crystal packing showing the hydrogen bonds in the monoclinic unit cell of **2** (X-ray crystallography of **2**), Figures S9 and S10; ESI mass spectra of the separated HPLC peak regions and enlargements of the ESI mass spectrum dication peaks of **3**, Figures S11–S20; National Cancer Institute (NCI) Developmental Therapeutics Program (DTP) 60-cancer cell 5-dose testing of **1**, **2**, and **3** (with cellular p53 status), Table S1; Antineoplastic activity in the NCI DTP 60-cancer cell 5-dose testing of **1**, Figures S21–S23; Antineoplastic activity in the NCI DTP 60-cancer cell 5-dose testing of **2**, Figures S24–S26; Antineoplastic activity in the NCI DTP 60-cancer cell 5-dose testing of **3**, Figures S27–S29.
