# Supplementary material for: Novel Antineoplastic Inducers of Mitochondrial Apoptosis in Human Cancer Cells
Source: Molecules. 2024 Feb 19;29(4):914. doi: 10.3390/molecules29040914 (PMC10892984; doi:10.3390/molecules29040914)
Supplement: Supplementary file 1 [file molecules-29-00914-s001.zip › molecules-2829501-supplementary.pdf]

Supplementary Information

Novel Antineoplastic Inducers of Mitochondrial Apoptosis in Human Cancer Cells

Andreas J. Kesel[1,*]

[1]Chammünsterstr. 47, D-81827 München, Bavaria/Bayern, Germany

***Corresponding author:** Andreas J. Kesel, Chammünsterstr. 47, D-81827 München, Bavaria/Bayern, Germany; Phone: +49 (0)176-87974849; E-mail: andreasj.kesel@gmx.de

Table of Contents

Figure S1. The 400.13 MHz ^{1}H-NMR spectrum (in DMSO-d_6) of the salt-containing compound **1** (**PENTA**) 6

Figure S2. The 100.62 MHz ^{13}C-NMR spectrum (in DMSO-d_6) of the salt-containing compound **1** (**PENTA**) 7

Figure S3. The *Fourier*–transform infrared (FT–IR) absorption spectra of the salt-containing compound **1** (**PENTA**) and of the reference substance amantadine hydrochloride (1-adamantanammonium chloride) 8

Figure S4. The 100.62 MHz ^{13}C-Distortionless Enhancement by Polarization Transfer Including Detection of Quaternary Nuclei (DEPTQ) (DEPTQ ^{13}C-NMR) spectrum (in DMSO-d_6) of the salt-containing compound **1** (**PENTA**) 9

Figure S5. The 400.13 MHz gradient-selected Correlation Spectroscopy (gs-COSY) two-dimensional ^{1}H–^{1}H-correlation spectrum (in DMSO-d_6) of the salt-containing compound **1** (**PENTA**) 10

Figure S6. The 400.13/100.62 MHz gradient-selected Heteronuclear Multiple Quantum Coherence (gs-HMQC) two-dimensional ^{1}H–^{13}C-correlation spectrum (in DMSO-d_6) of the salt-containing compound **1** (**PENTA**) 11

Figure S7. The 400.13/100.62 MHz gradient-selected Heteronuclear Multiple Bond Correlation (gs-HMBC) two-dimensional ^{1}H–^{13}C-correlation spectrum (in DMSO-d_6) of the salt-containing compound **1** (**PENTA**) 12

Figure S8. The *Fourier*–transform infrared (FT–IR) absorption spectra of the colchic(in)oid 10-(2-carbamothioylhydrazinyl)-10-demethoxycolchicine monohydrate × ⅔ (ethyl acetate) = compound **2**, and of the reference substance (−)-colchicine sesquihydrate______________ 13

Figure S9. One formula unit (Z = 2) in the monoclinic (space group $P2_1$) unit cell of compound **2** crystallized as hydrate (ethyl acetate) solvate___________________________ 14

Figure S10. Crystal packing showing the hydrogen bonds (H bonds, shown as colored dashed lines) in the monoclinic (space group $P2_1$) unit cell (Z = 2) of compound **2** crystallized as hydrate (ethyl acetate) solvate_____________________________________ 15

Figure S11. The ESI mass spectrum of the separated HPLC peak region 7.842−7.925 min with the ammonium trication ionization species of compound **3**________________________ 16

Figure S12. The ESI mass spectrum of the separated HPLC peak region 8.508−8.558 min with the ammonium dication ionization species of compound **3**________________________ 17

Figure S13. The ESI mass spectrum of the separated HPLC peak region 8.608−8.658 min with the ammonium monocation ionization species of compound **3**_____________________ 18

Figure S14. The ESI mass spectrum of the separated HPLC peak region 8.691−8.741 min with the ammonium monocation ionization species of compound **3**_____________________ 19

Figure S15. The ESI mass spectrum of the separated HPLC peak region 9.024−9.074 min with the ylide dication ionization species of compound **3**_____________________________ 20

Figure S16. The ESI mass spectrum of the separated HPLC peak region 9.740−9.973 min with the ylide monocation ionization species of compound **3**__________________________ 21

Figure S17. The ESI mass spectrum of the separated HPLC peak region 13.386−13.453 min with the neutral ylide ionization species of compound **3**_____ 22

Figure S18. The enlargement of the ESI mass spectrum peak *m*/*z* 483.2727 of the separated HPLC peak region 9.024−9.074 min with the ylide dication ionization species of compound **3**_____ 23

Figure S19. The enlargement of the ESI mass spectrum peak *m*/*z* 609.8643 of the separated HPLC peak region 9.757−9.973 min with the ylide monocation ionization species of compound **3**_____ 24

Figure S20. The enlargement of the ESI mass spectrum peak *m*/*z* 356.6814 of the separated HPLC peak region 8.706−8.758 min with the ammonium monocation ionization species of compound **3**_____ 25

Table S1. National Cancer Institute (NCI) Developmental Therapeutics Program (DTP) 60-cancer cell 5-dose testing of compound **1** (**PT162**), compound **2** (**PT166**), and compound **3** (**PT167**) (with cellular p53 status)_____ 26−30

Figure S21. Graphic allover presentation of the antineoplastic activity in the National Cancer Institute (NCI) Developmental Therapeutics Program (DTP) 60-cancer cell 5-dose testing exhibited by the pure drug compound **1** (**PT162**, **NSC 796018**)_____ 31

Figure S22. Tabular list of the antineoplastic activity exhibited by the pure drug compound **1** (**PT162**, **NSC 796018**) in the National Cancer Institute (NCI) Developmental Therapeutics Program (DTP) 60-cancer cell 5-dose testing_____ 32

Figure S23. Graphic table presentation of the antineoplastic activity exhibited by the pure drug compound **1** (**PT162**, **NSC 796018**) in the National Cancer Institute (NCI) Developmental Therapeutics Program (DTP) 60-cancer cell 5-dose testing_____ 33

Figure S24. Graphic allover presentation of the antineoplastic activity in the National Cancer Institute (NCI) Developmental Therapeutics Program (DTP) 60-cancer cell 5-dose testing exhibited by the drug compound **2** (**PT166**, **NSC 750423**)____________________________ 35

Figure S25. Tabular list of the antineoplastic activity exhibited by the drug compound **2** (**PT166**, **NSC 750423**) in the National Cancer Institute (NCI) Developmental Therapeutics Program (DTP) 60-cancer cell 5-dose testing_______________________________________ 36

Figure S26. Graphic table presentation of the antineoplastic activity exhibited by the drug compound **2** (**PT166**, **NSC 750423**) in the National Cancer Institute (NCI) Developmental Therapeutics Program (DTP) 60-cancer cell 5-dose testing___________________________ 37

Figure S27. Graphic allover presentation of the antineoplastic activity in the National Cancer Institute (NCI) Developmental Therapeutics Program (DTP) 60-cancer cell 5-dose testing exhibited by the drug compound **3** (**PT167**, **NSC 799315**)____________________________ 39

Figure S28. Tabular list of the antineoplastic activity exhibited by the drug compound **3** (**PT167**, **NSC 799315**) in the National Cancer Institute (NCI) Developmental Therapeutics Program (DTP) 60-cancer cell 5-dose testing_______________________________________ 40

Figure S29. Graphic table presentation of the antineoplastic activity exhibited by the drug compound **3** (**PT167**, **NSC 799315**) in the National Cancer Institute (NCI) Developmental Therapeutics Program (DTP) 60-cancer cell 5-dose testing___________________________ 41

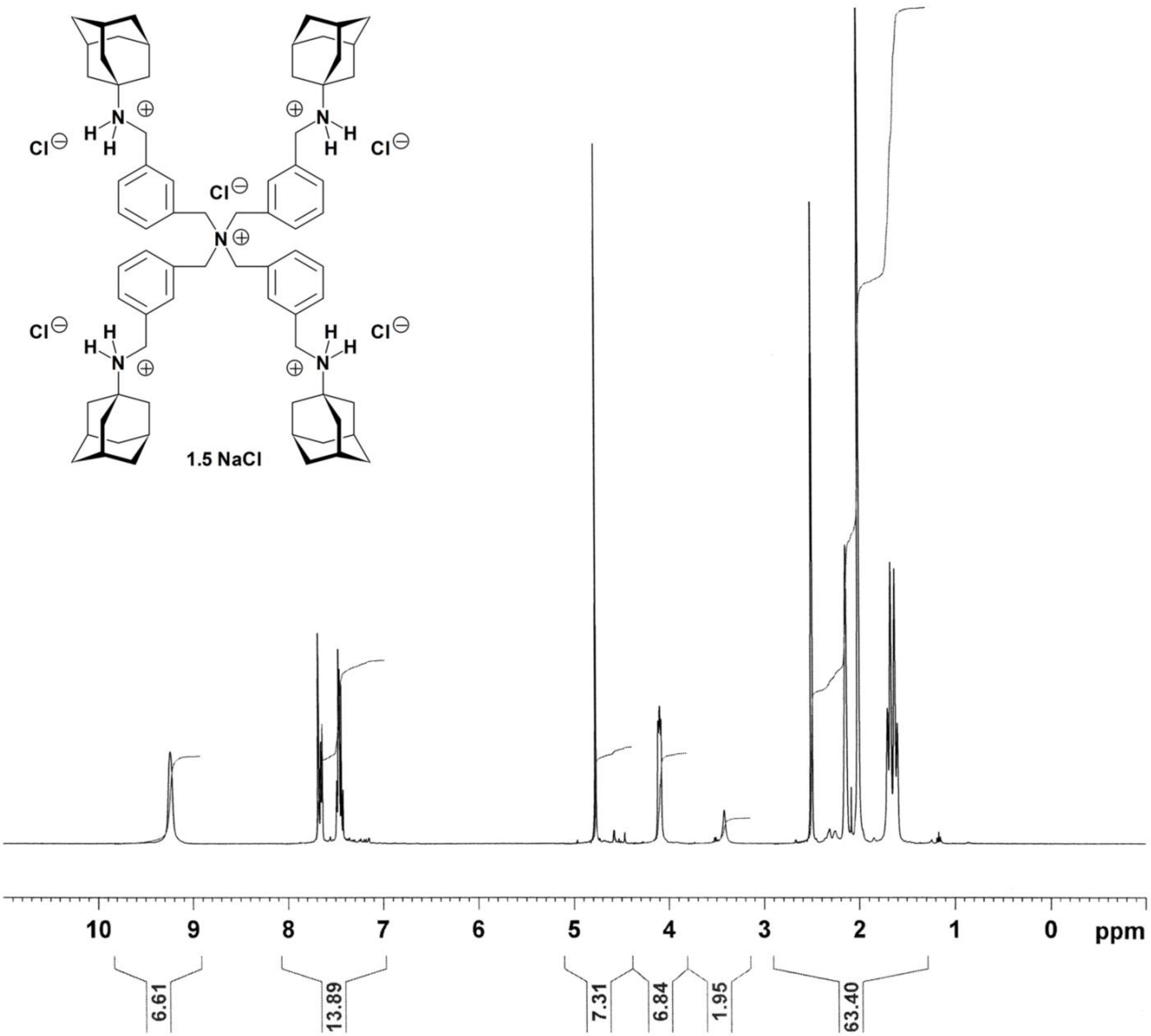

Figure S1. The 400.13 MHz ^{1}H-NMR spectrum (in DMSO-d_6) of the salt-containing polyammonium polycation tetrakis{3-[(tricyclo[3.3.1.1^{3,7}] decan-1-ammonio)methyl]benzyl}ammonium pentachloride × 1.5 (sodium chloride) = salt-containing compound **1** (**PENTA**).

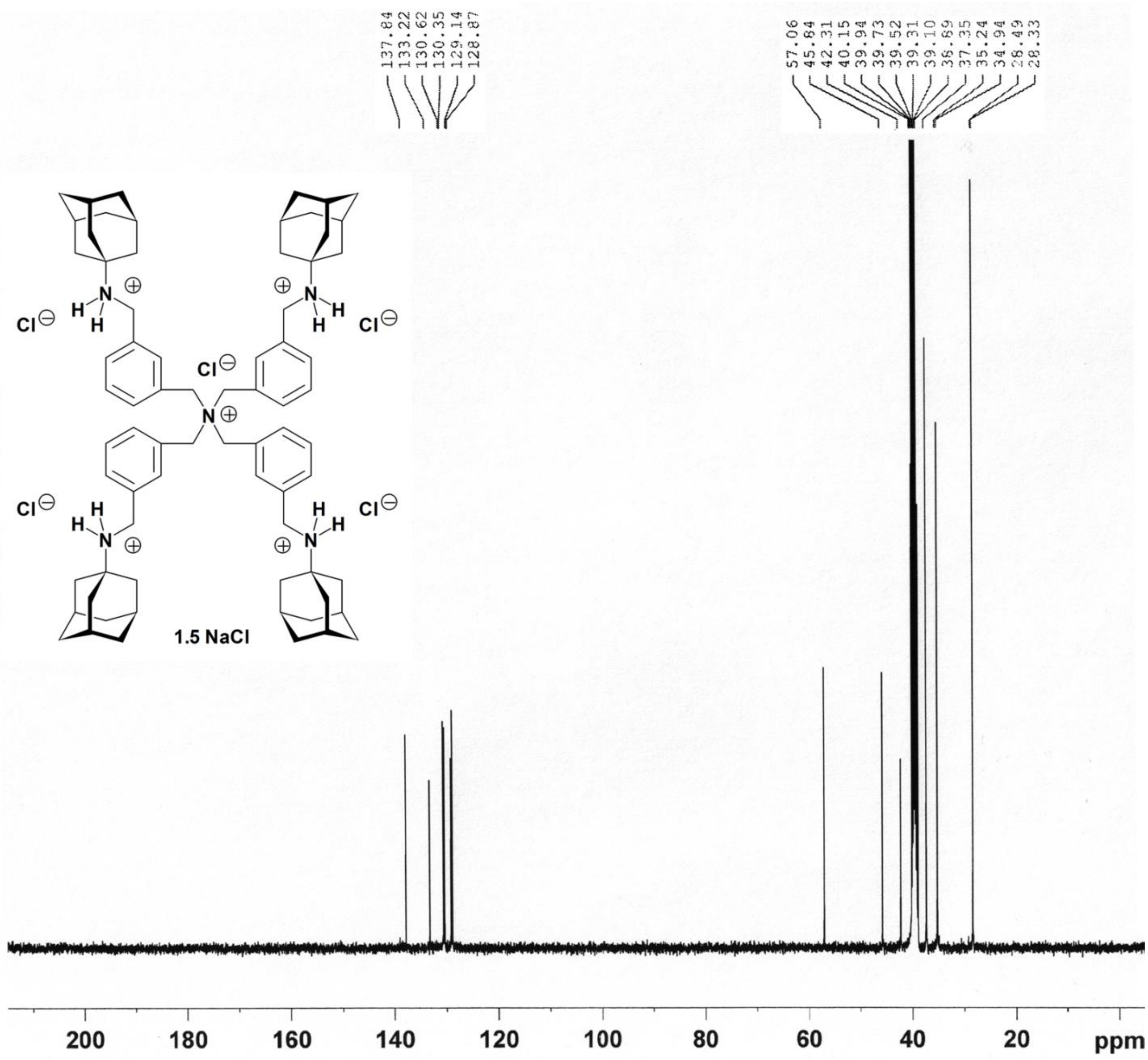

Figure S2. The 100.62 MHz ^{13}C-NMR spectrum (in DMSO-d_6) of the salt-containing polyammonium polycation tetrakis{3-[(tricyclo[3.3.1.1^{3,7}]decan-1-ammonio)methyl]benzyl} ammonium pentachloride × 1.5 (sodium chloride) = salt-containing compound **1** (**PENTA**).

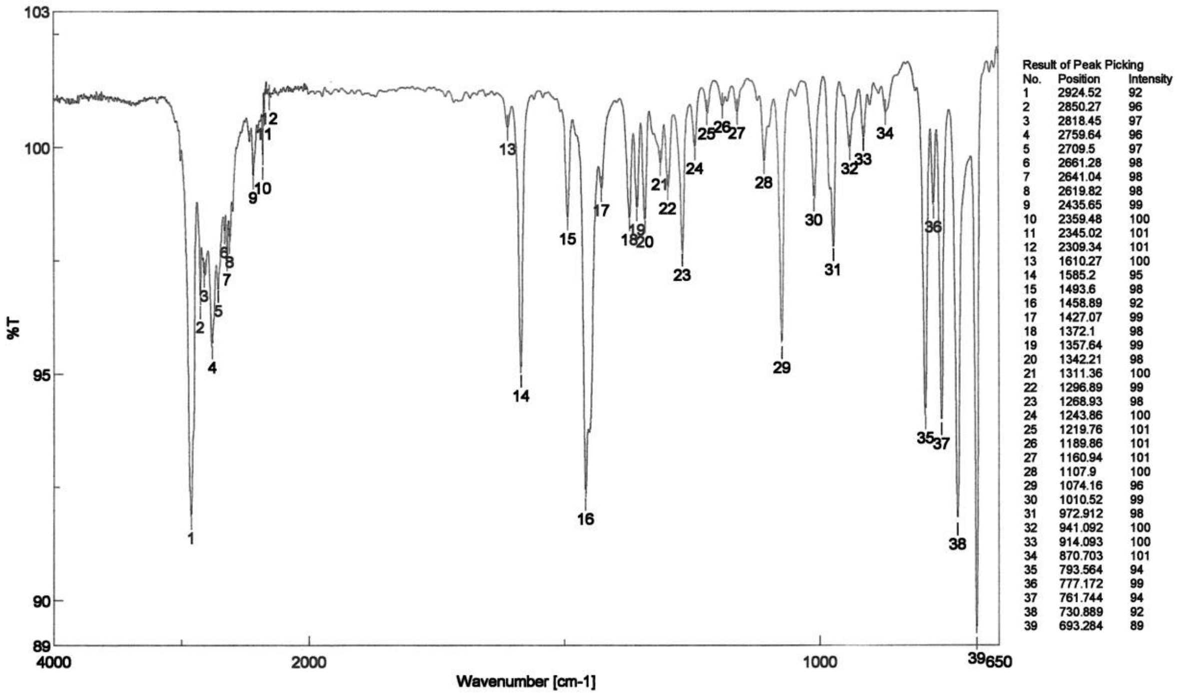

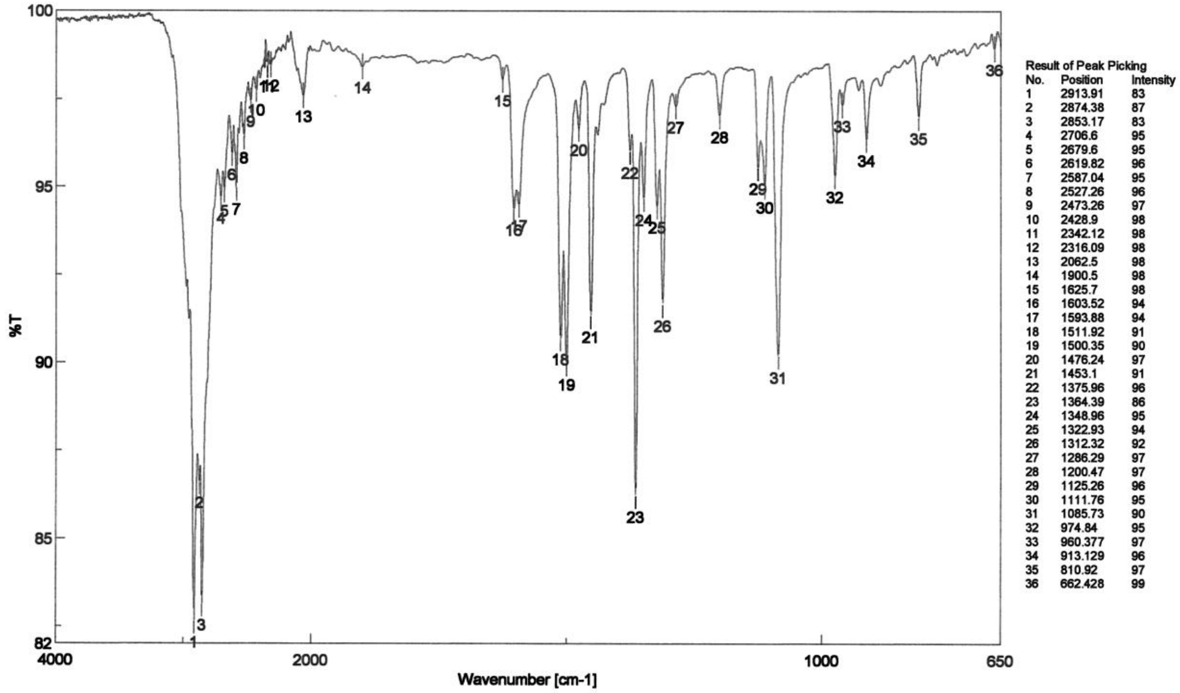

Figure S3. The *Fourier*–transform infrared (FT–IR) absorption spectra of the salt-containing tetrakis{3-[(tricyclo[3.3.1.1^{3,7}]decan-1-ammonio)methyl]benzyl}ammonium pentachloride × 1.5 (sodium chloride) = salt-containing compound **1** (**PENTA**) (top), and of the reference substance amantadine hydrochloride (1-adamantanammonium chloride) (bottom), both recorded with neat substance.

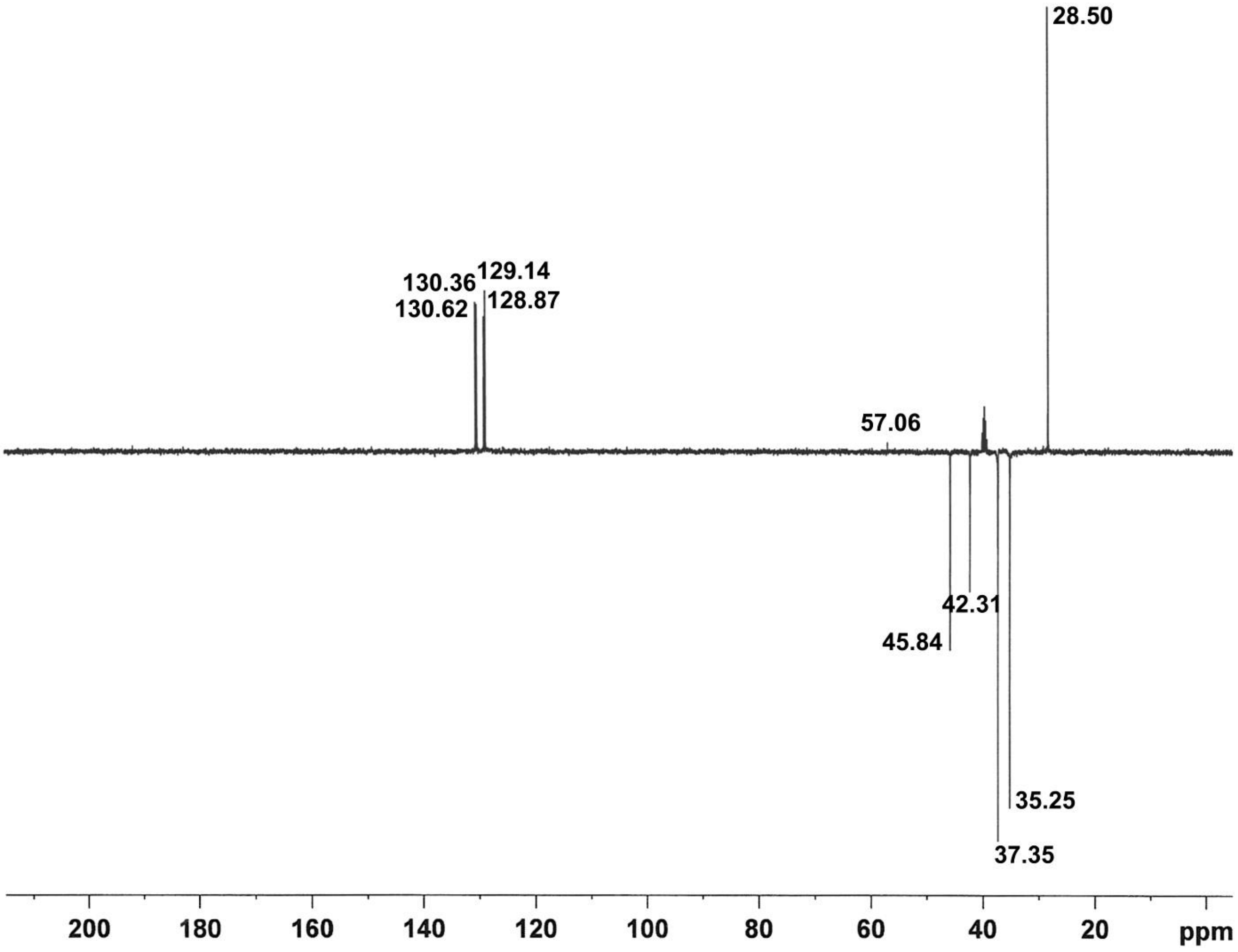

Figure S4. The 100.62 MHz ^{13}C-Distortionless Enhancement by Polarization Transfer Including Detection of Quaternary Nuclei (DEPTQ) (DEPTQ ^{13}C-NMR) spectrum (in DMSO-d_6) of the salt-containing polyammonium polycation tetrakis{3-[(tricyclo[3.3.1.1^{3,7}]decan-1-ammonio)methyl]benzyl}ammonium pentachloride × 1.5 (sodium chloride) = salt-containing compound **1** (**PENTA**). Quaternary carbons (C), methine (CH) and methyl (CH_3) group moieties are of positive phase, methylene (CH_2) group moieties are of negative phase. The quaternary carbon δ 57.06 ppm (α-C) is weak in intensity. The aromatic quaternary carbons δ 133.22 ppm (C-3) and 137.84 ppm (C-1) could not being detected.

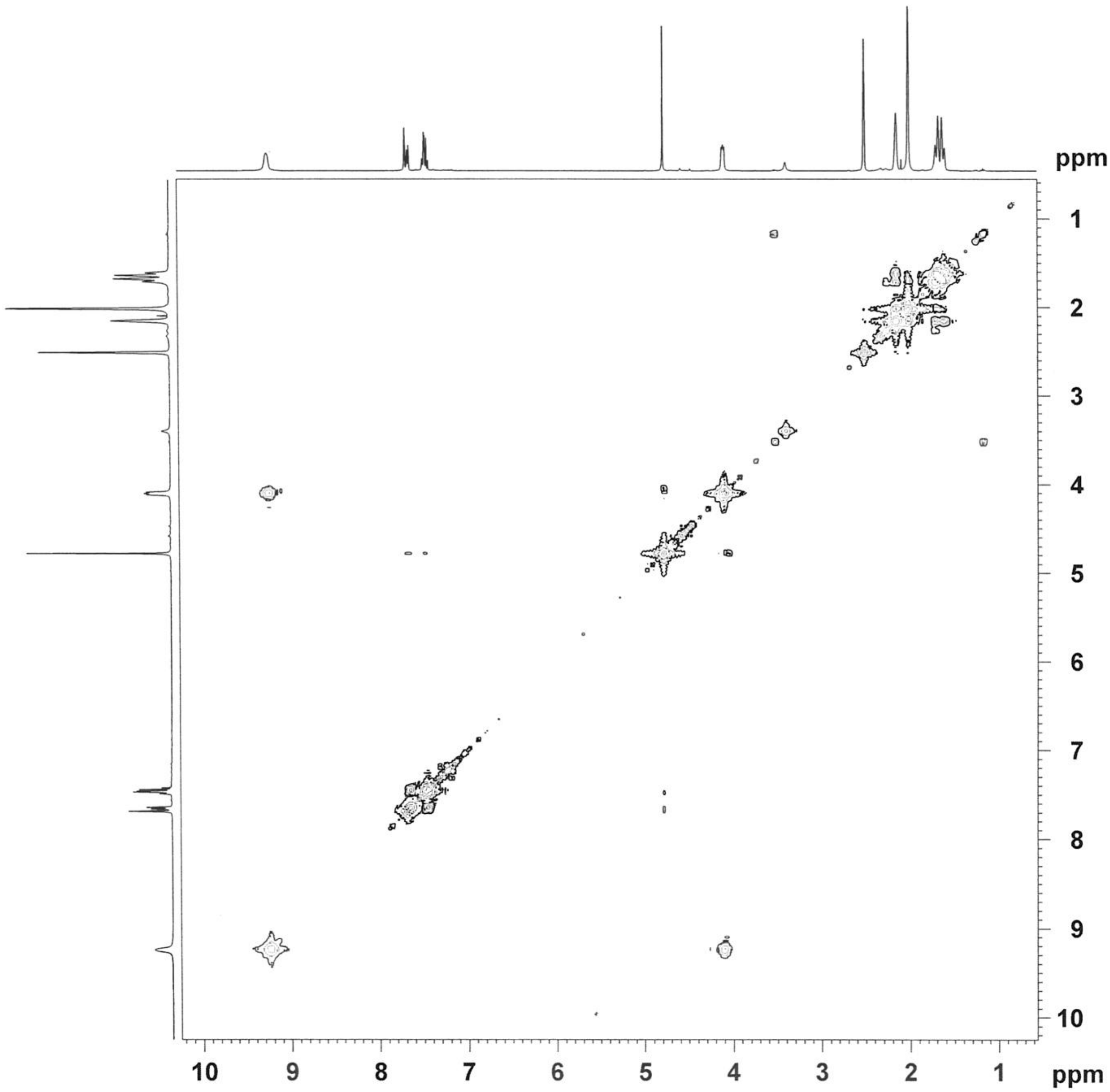

Figure S5. The 400.13 MHz gradient-selected Correlation Spectroscopy (gs-COSY) two-dimensional ^{1}H–^{1}H-correlation spectrum (in DMSO-d_6) of the salt-containing polyammonium polycation tetrakis{3-[(tricyclo[3.3.1.1^{3,7}]decan-1-ammonio)methyl]benzyl}ammonium pentachloride × 1.5 (sodium chloride) = salt-containing compound **1** (**PENTA**).

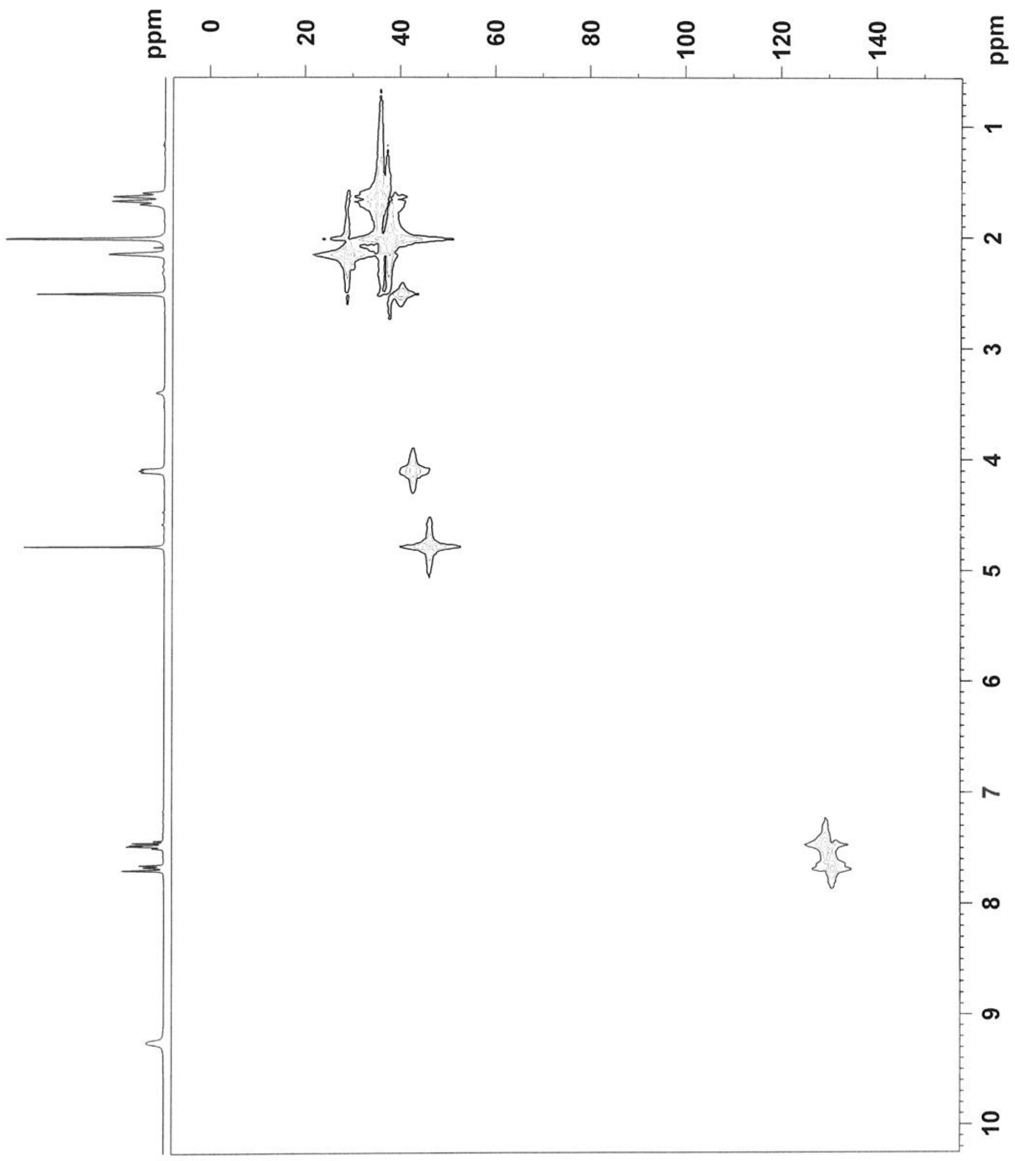

Figure S6. The 400.13/100.62 MHz gradient-selected Heteronuclear Multiple Quantum Coherence (gs-HMQC) two-dimensional ^{1}H–^{13}C-correlation spectrum (in DMSO-d_6) of the salt-containing polyammonium polycation tetrakis{3-[(tricyclo[3.3.1.1^{3,7}]decan-1-ammonio)methyl] benzyl}ammonium pentachloride × 1.5 (sodium chloride) = salt-containing compound **1** (**PENTA**).

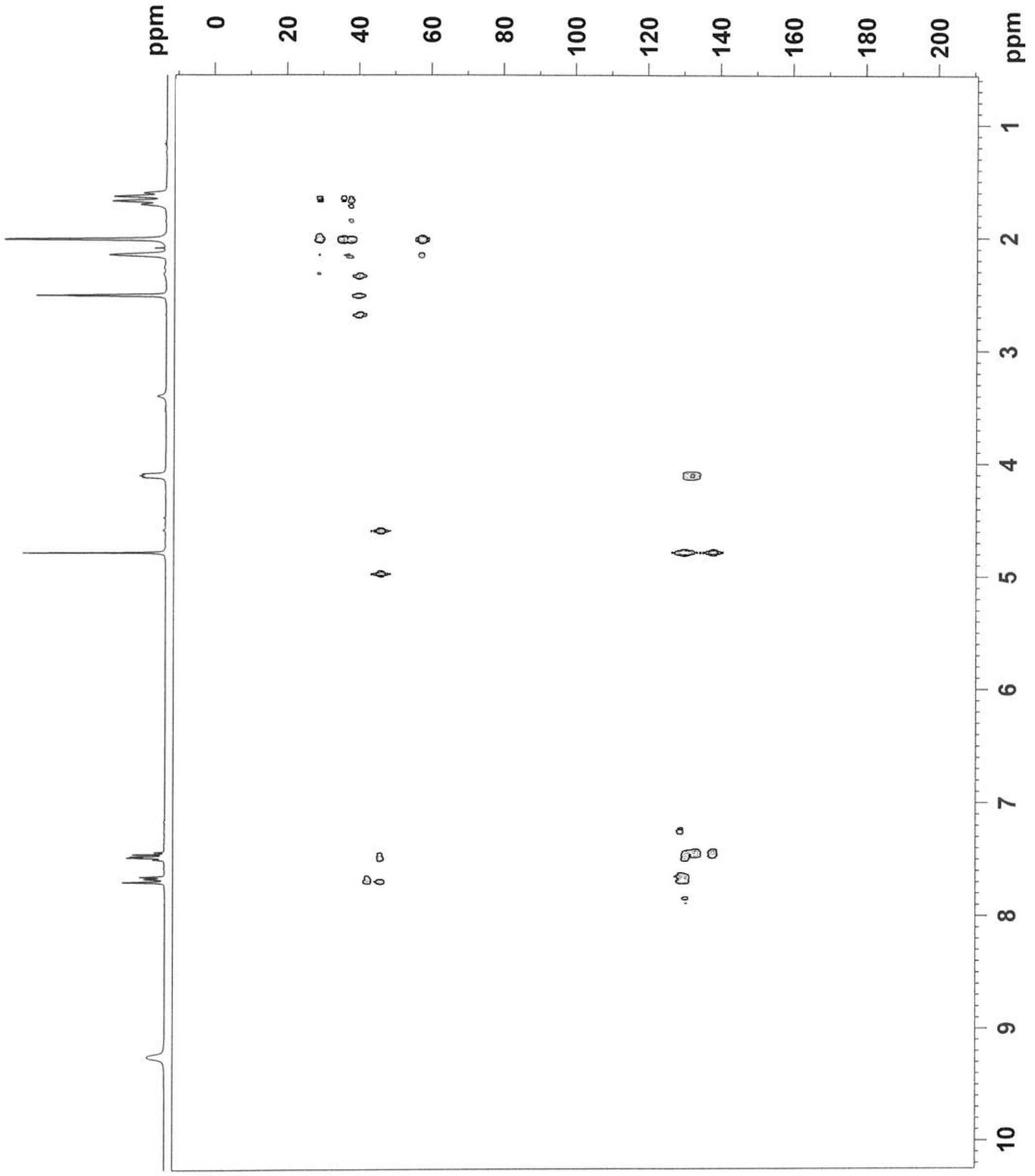

Figure S7. The 400.13/100.62 MHz gradient-selected Heteronuclear Multiple Bond Correlation (gs-HMBC) two-dimensional $^{1}H-^{13}C$-correlation spectrum (in DMSO-d_6) of the salt-containing polyammonium polycation tetrakis{3-[(tricyclo[3.3.1.1^{3,7}]decan-1-ammonio)methyl] benzyl}ammonium pentachloride × 1.5 (sodium chloride) = salt-containing compound **1** (**PENTA**).

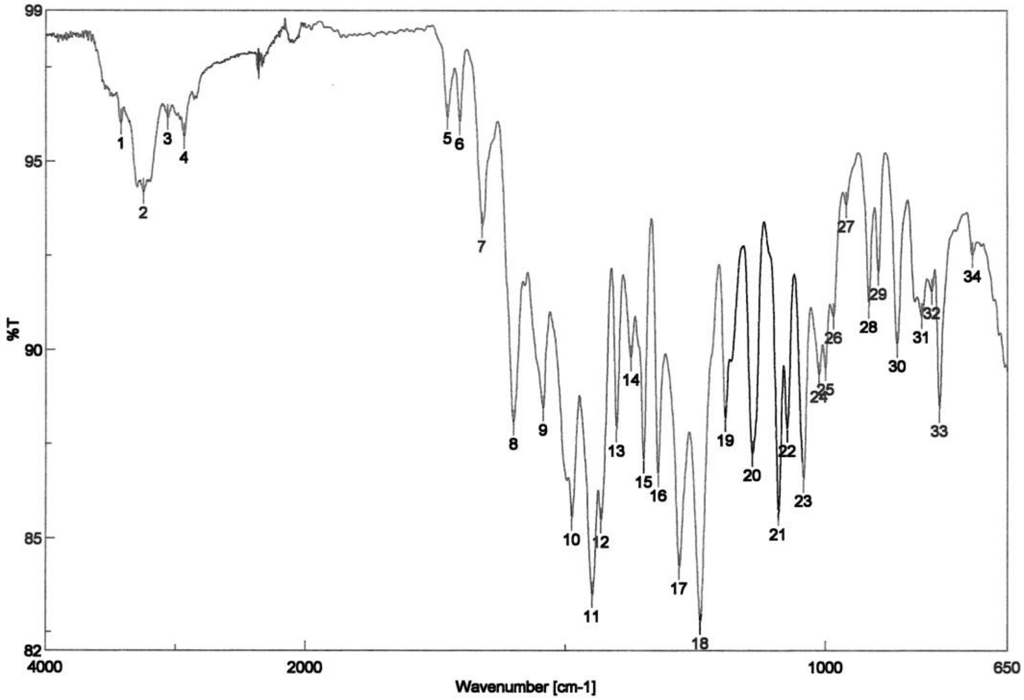

Result of Peak Picking

No.	Position	Intensity
1	3419.17	96
2	3246.57	94
3	3061.44	96
4	2934.16	96
5	1726.94	96
6	1702.84	96
7	1660.41	93
8	1599.66	88
9	1542.77	88
10	1487.81	86
11	1448.28	83
12	1431.89	85
13	1402	88
14	1374.03	90
15	1349.93	87
16	1321.96	87
17	1281.47	84
18	1240.97	83
19	1192.76	88
20	1140.69	87
21	1090.55	86
22	1073.19	88
23	1042.34	87
24	1012.45	89
25	999.91	89
26	984.482	91
27	960.377	94
28	916.986	91
29	898.666	92
30	862.025	90
31	814.777	91
32	795.493	92
33	781.029	88
34	717.39	93

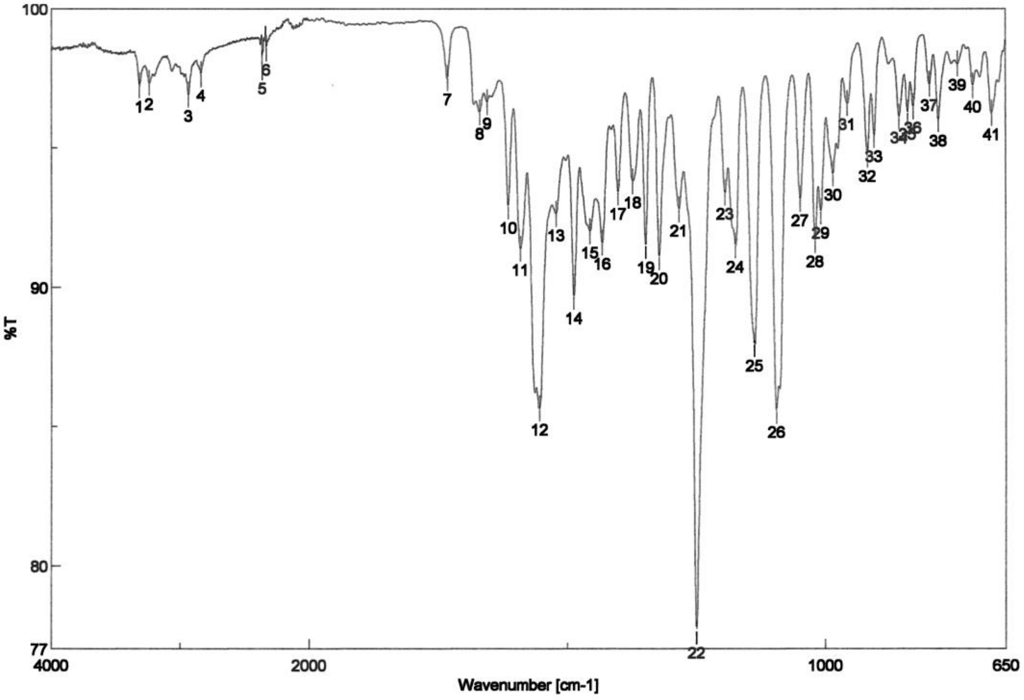

Result of Peak Picking

No.	Position	Intensity
1	3315.03	97
2	3238.86	97
3	2935.13	97
4	2837.74	98
5	2361.41	98
6	2329.59	99
7	1731.76	97
8	1669.09	96
9	1654.62	97
10	1614.13	93
11	1590.99	91
12	1554.34	86
13	1521.56	93
14	1486.85	90
15	1455.99	92
16	1431.89	92
17	1401.03	93
18	1373.07	94
19	1348	91
20	1321.96	91
21	1283.39	93
22	1249.65	78
23	1193.72	93
24	1173.47	91
25	1136.83	88
26	1094.4	86
27	1048.12	93
28	1019.19	92
29	1008.59	93
30	984.482	94
31	956.52	97
32	917.95	95
33	905.415	95
34	856.239	96
35	839.847	96
36	829.241	96
37	797.421	97
38	780.065	96
39	743.424	98
40	713.533	97
41	676.892	96

Figure S8. The *Fourier*–transform infrared (FT–IR) absorption spectra of the colchic(in)oid 10-(2-carbamothioylhydrazinyl)-10-demethoxycolchicine monohydrate × ⅔ (ethyl acetate) = compound **2** (top), and of the reference substance (−)-colchicine sesquihydrate [(−)-colchicine × 1½ H_2O] (bottom), both recorded with neat substance.

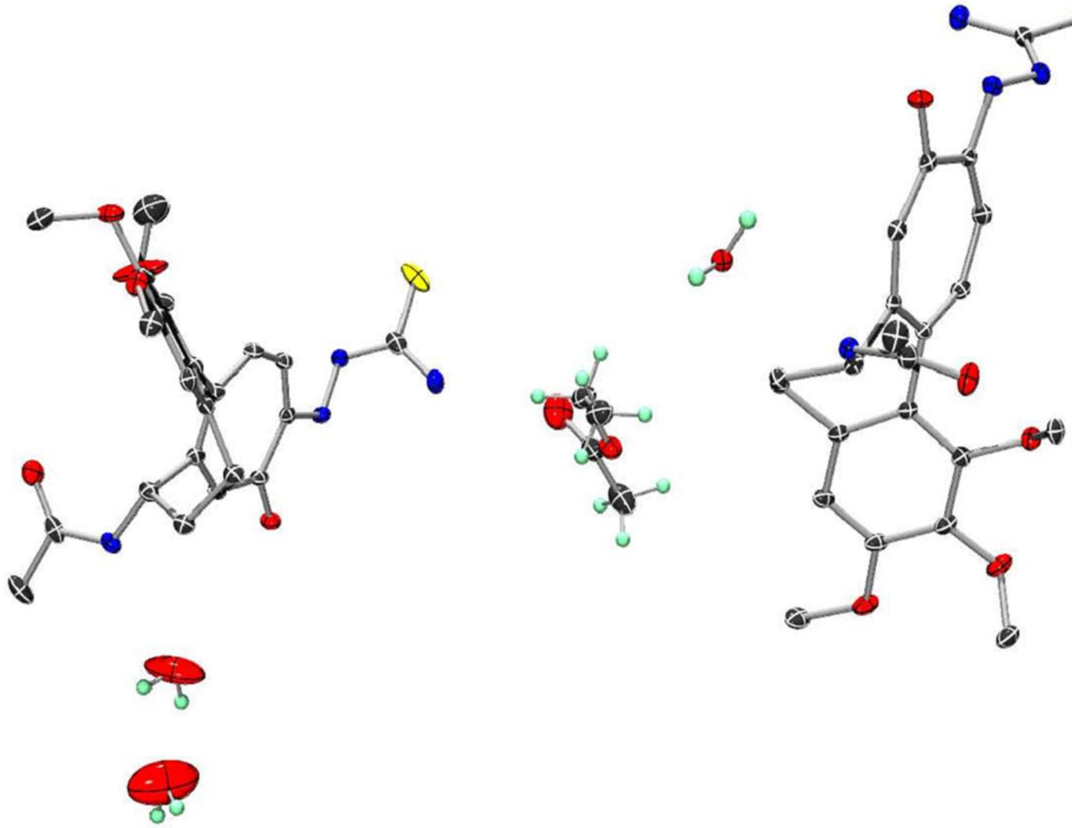

Figure S9. One formula unit (Z = 2) in the monoclinic (space group $P2_1$) unit cell of compound **2** (**PT166**) ($C_{24}H_{33}N_4O_{7\cdot50}S$) crystallized as hydrate (ethyl acetate) solvate (2 $C_{22}H_{26}N_4O_5S$ × 3 H_2O × $C_4H_8O_2$) $C_{48}H_{66}N_8O_{15}S_2$ (M = 1059.21 g/mol) as found in the single crystal.

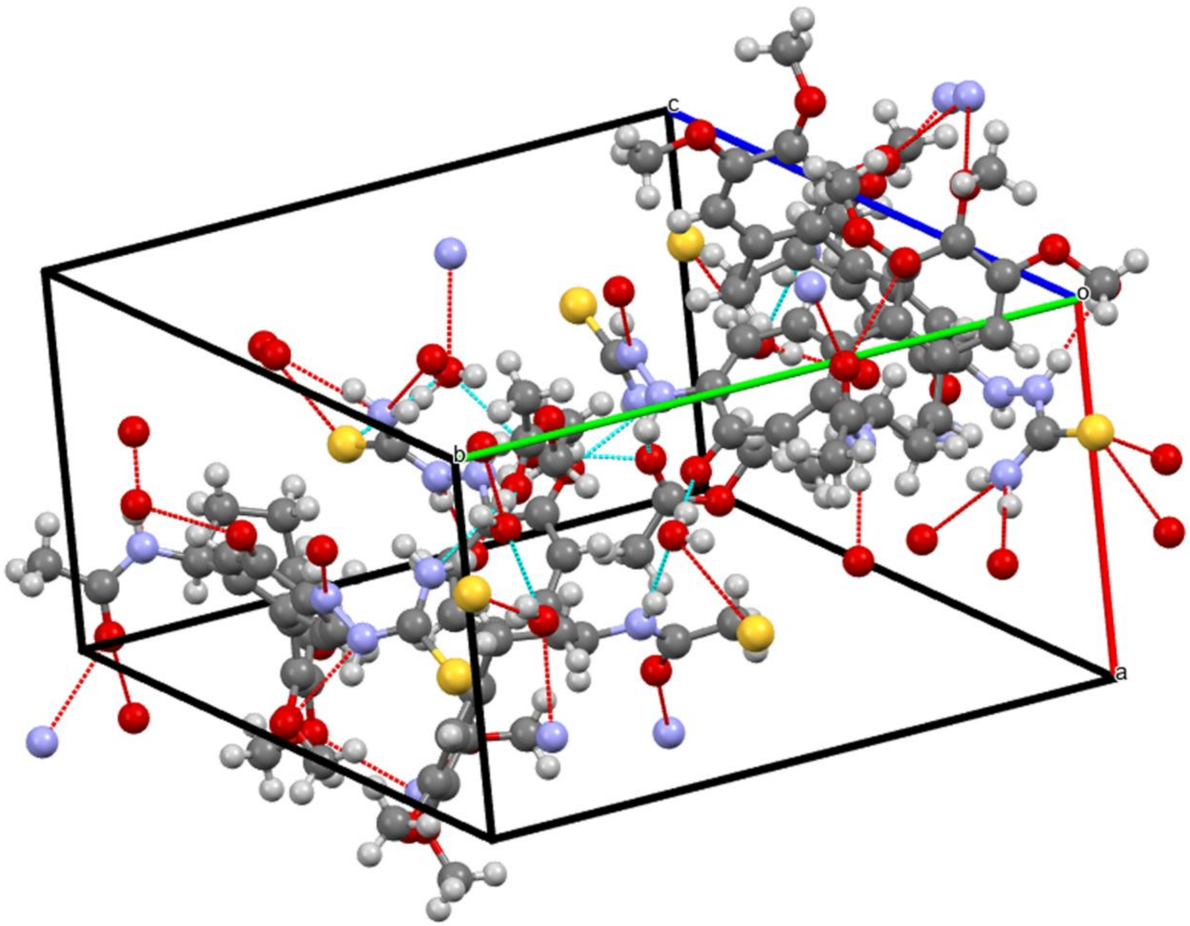

Figure S10. Crystal packing showing the hydrogen bonds (H bonds, shown as colored dashed lines) in the monoclinic (space group $P2_1$) unit cell (Z = 2) of compound **2** (**PT166**) crystallized as hydrate (ethyl acetate) solvate (2 $C_{22}H_{26}N_4O_5S$ × 3 H_2O × $C_4H_8O_2$) $C_{48}H_{66}N_8O_{15}S_2$ (M = 1059.21 g/mol). Unit cell dimensions (a, b, c are indicated from the origin o): a = 9.1886(5) Å, b = 20.9047(10) Å, c = 13.9841(7) Å, α = 90.00°, β = 106.153(2)°, γ = 90.00°, V = 2580.1(2) Å^3.

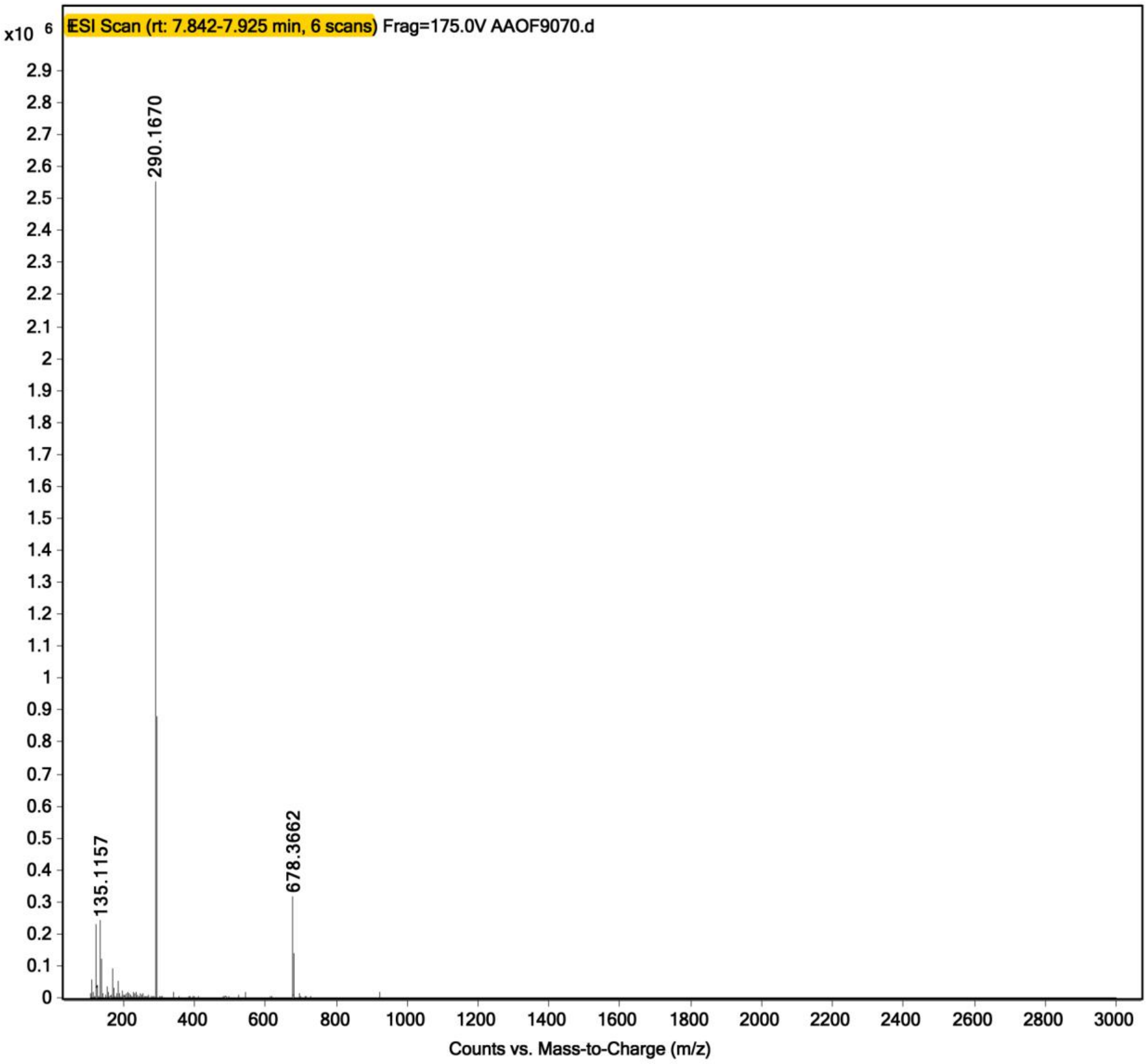

Figure S11. The ESI mass spectrum of the separated HPLC peak region 7.842−7.925 min (see Figure 15A) with the ammonium trication ionization species of compound **3**. The ESI−MS fragment peaks (relative intensity of the 100% base peak) were *m*/*z* 290.1670 (100%), 678.3662 (12.5%), and 135.1157 (9.6%).

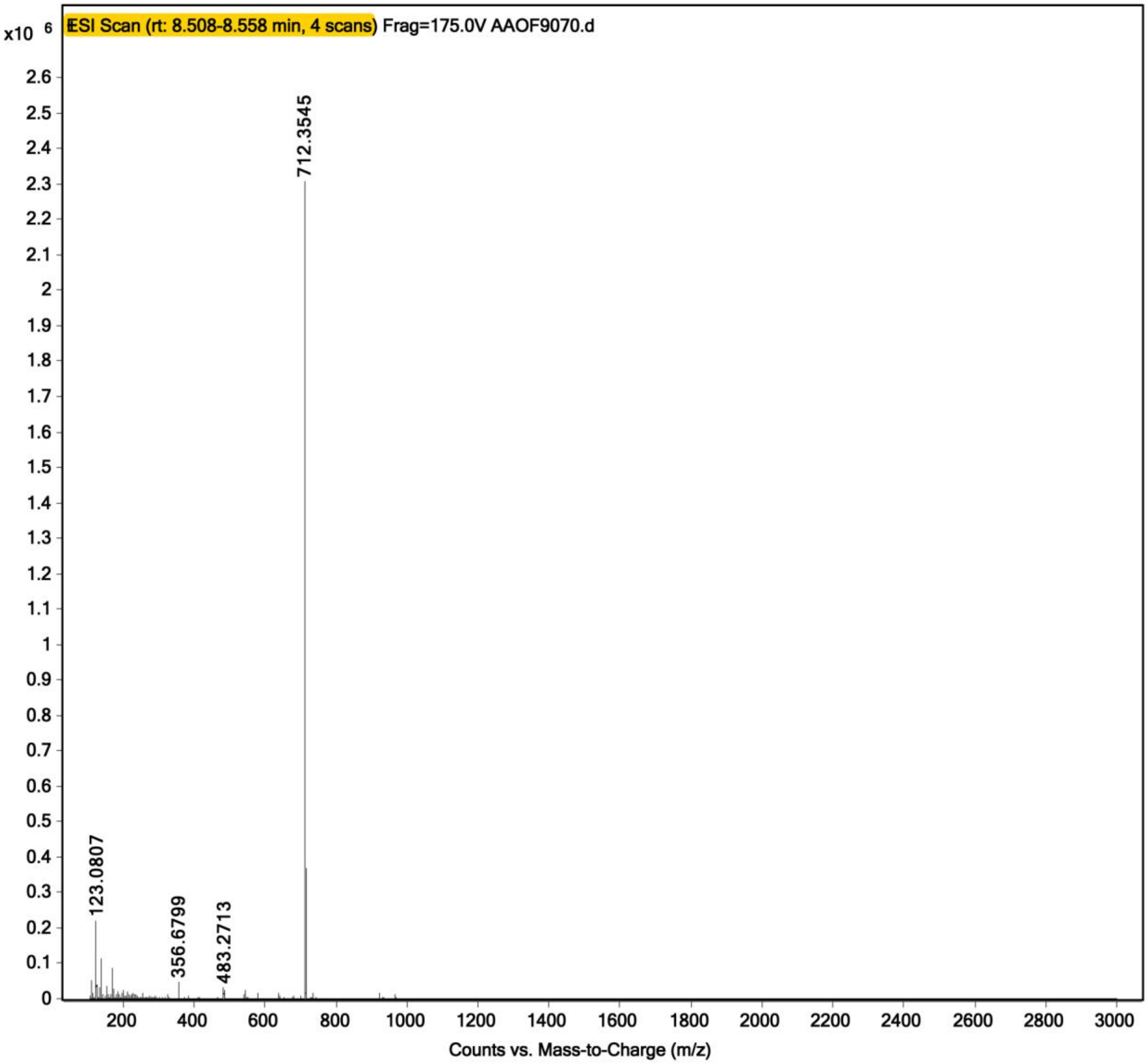

Figure S12. The ESI mass spectrum of the separated HPLC peak region 8.508−8.558 min (see Figure 15A) with the ammonium dication ionization species of compound **3**. The ESI−MS fragment peaks (relative intensity of the 100% base peak) were *m*/*z* 712.3545 (100%), 123.0807 (16.5%), 356.6799 (2.1%), and 483.2713 (1.3%).

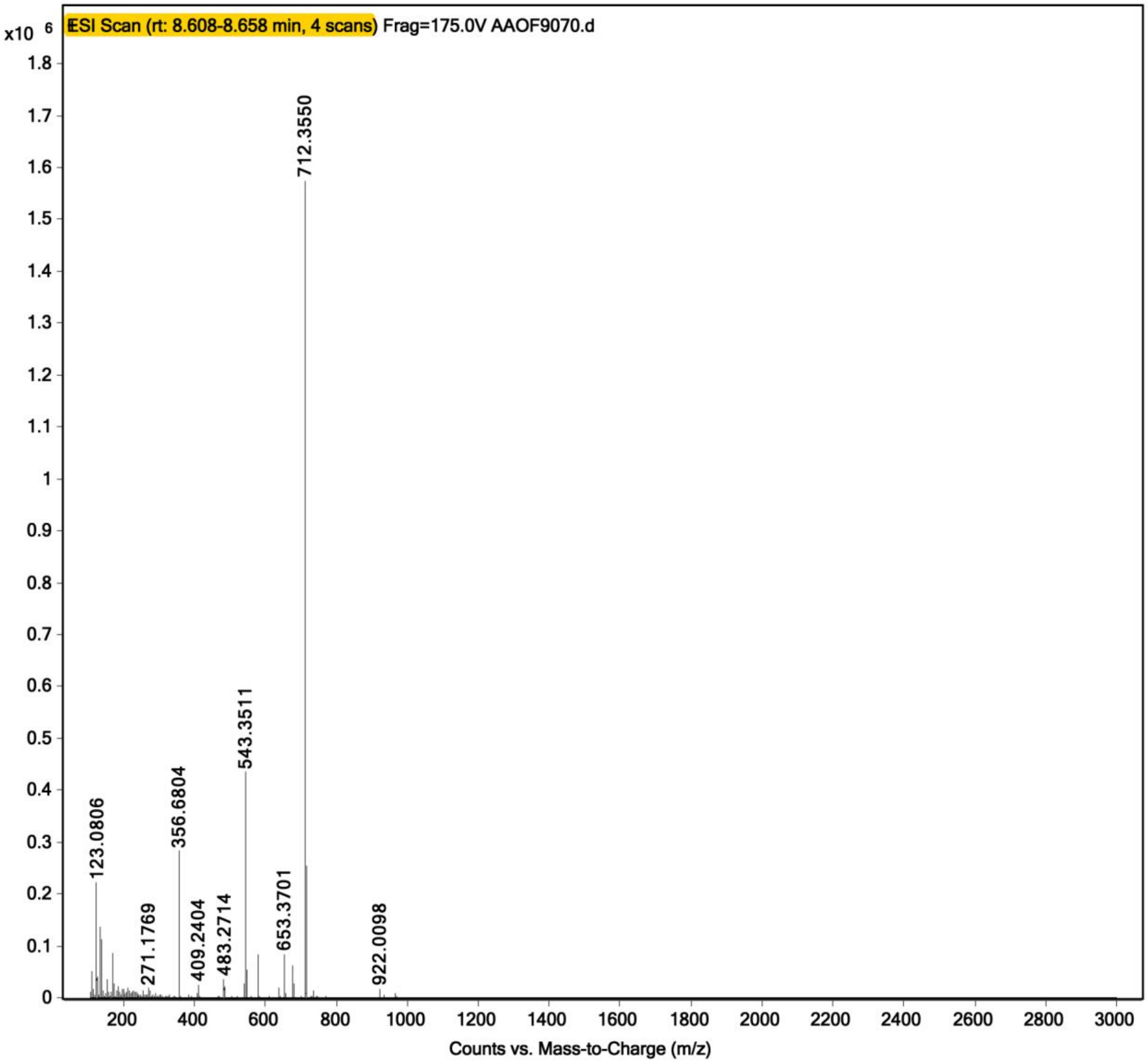

Figure S13. The ESI mass spectrum of the separated HPLC peak region 8.608−8.658 min (see Figure 15A) with the ammonium monocation ionization species of compound **3**. The ESI−MS fragment peaks (relative intensity of the 100% base peak) were *m*/*z* 712.3550 (100%), 543.3511 (27.9%), 356.6804 (18.1%), 123.0806 (14.2%), 653.3701 (5.4%), 483.2714 (2.5%), and 409.2404 (1.7%). The *m*/*z* 922.0098 is an internal control substance (reference cation) added to the analysis for auto-calibration of the mass spectrometer.

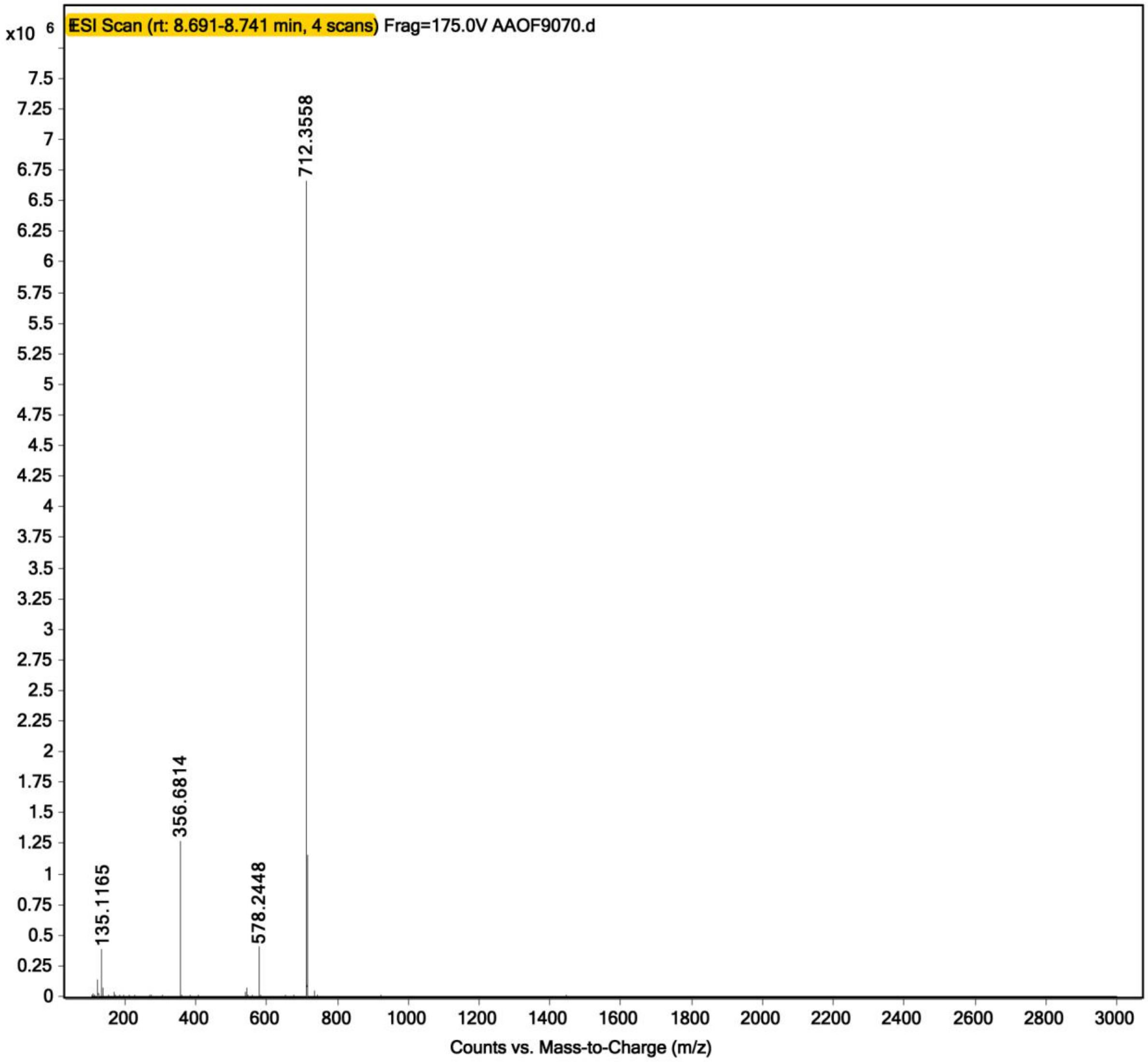

Figure S14. The ESI mass spectrum of the separated HPLC peak region 8.691−8.741 min (see Figure 15A) with the ammonium monocation ionization species of compound **3**. The ESI−MS fragment peaks (relative intensity of the 100% base peak) were *m*/*z* 712.3558 (100%), 356.6814 (19.1%), 578.2448 (6.3%), and 135.1165 (5.8%).

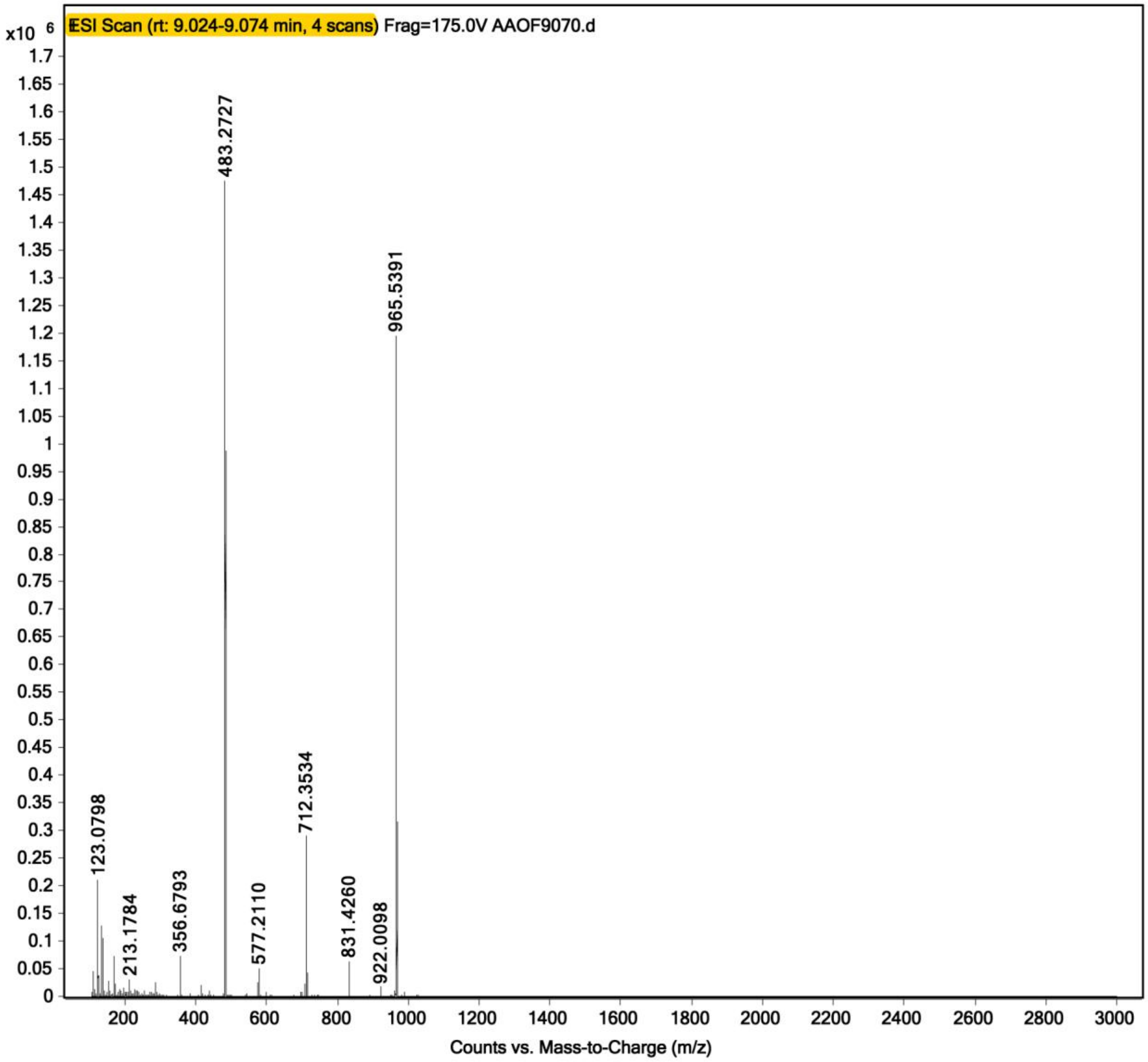

Figure S15. The ESI mass spectrum of the separated HPLC peak region 9.024−9.074 min (see Figure 15A) with the ylide dication ionization species of compound **3**. The ESI−MS fragment peaks (relative intensity of the 100% base peak) were *m*/*z* 483.2727 (100%), 965.5391 (80.9%), 712.3534 (19.5%), 123.0798 (14.1%), 356.6793 (5.0%), 831.4260 (4.3%), 577.2110 (3.4%), and 213.1784 (2.3%). The *m*/*z* 922.0098 is an internal control substance (reference cation) added to the analysis for auto-calibration of the mass spectrometer.

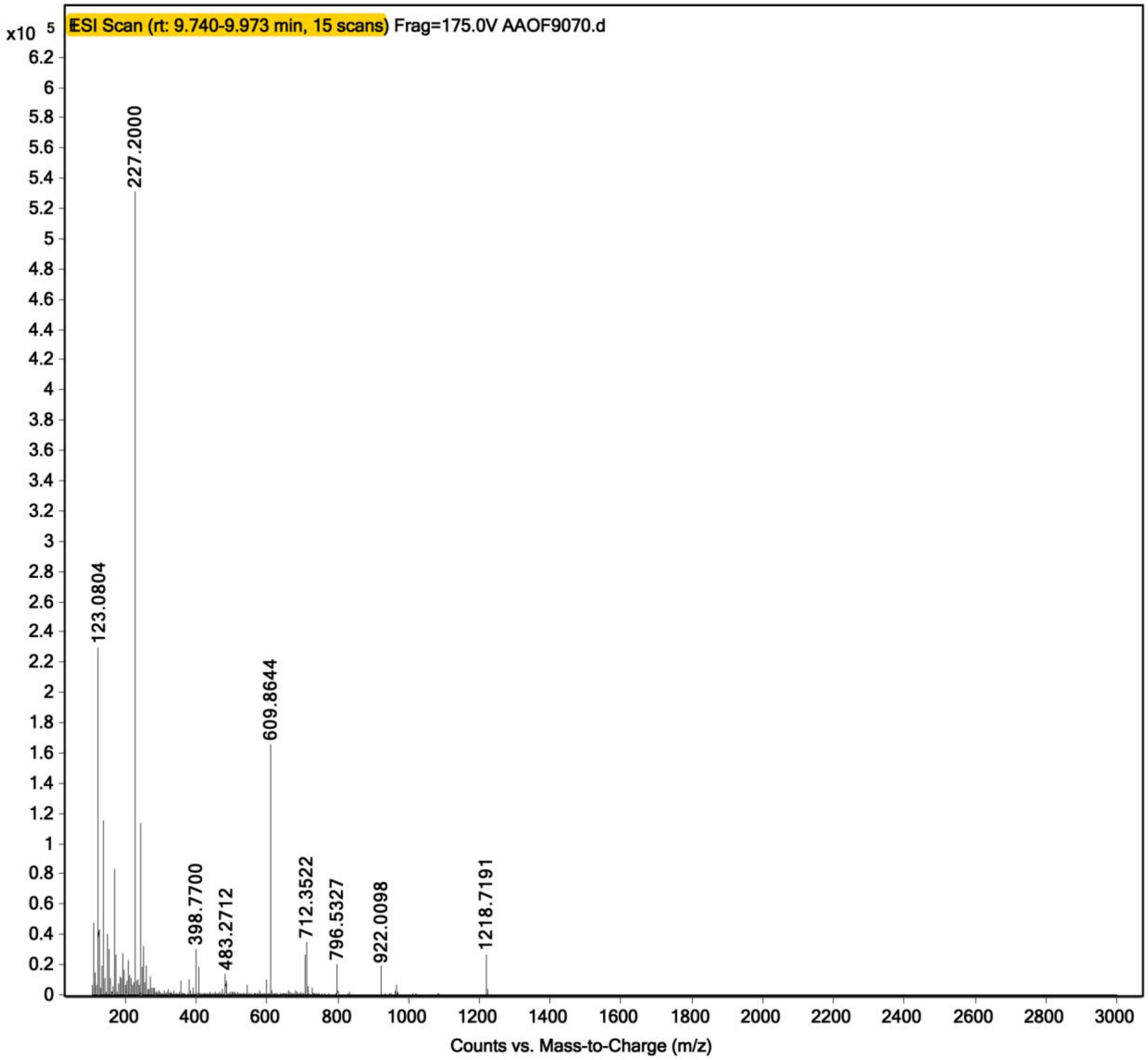

Figure S16. The ESI mass spectrum of the separated HPLC peak region 9.740−9.973 min (see Figure 15A) with the ylide monocation ionization species of compound **3**. The ESI−MS fragment peaks (relative intensity of the 100% base peak) were *m*/*z* 227.2000 (100%), 123.0804 (47.2%), 609.8644 (31.0%), 712.3522 (6.6%), 398.7700 (5.8%), 1218.7191 (5.0%), 796.5327 (4.0%), and 483.2712 (2.5%). The *m*/*z* 922.0098 is an internal control substance (reference cation) added to the analysis for auto-calibration of the mass spectrometer.

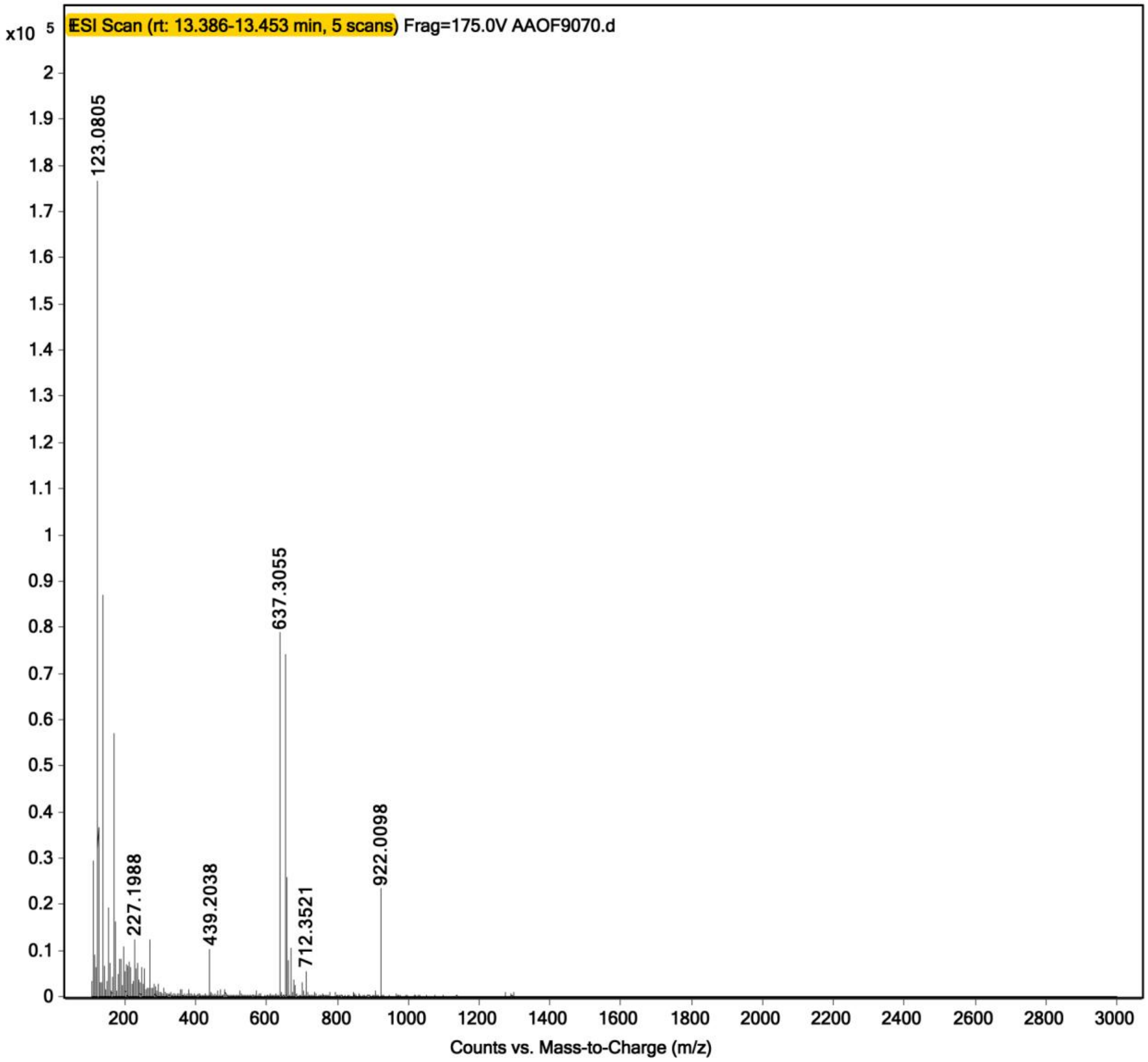

Figure S17. The ESI mass spectrum of the separated HPLC peak region 13.386−13.453 min (see Figure 15A) with the neutral ylide ionization species of compound **3**. The ESI−MS fragment peaks (relative intensity of the 100% base peak) were *m*/*z* 123.0805 (100%), 637.3055 (45.6%), 227.1988 (7.1%), 439.2038 (5.8%), and 712.3521 (3.2%). The *m*/*z* 922.0098 is an internal control substance (reference cation) added to the analysis for auto-calibration of the mass spectrometer.

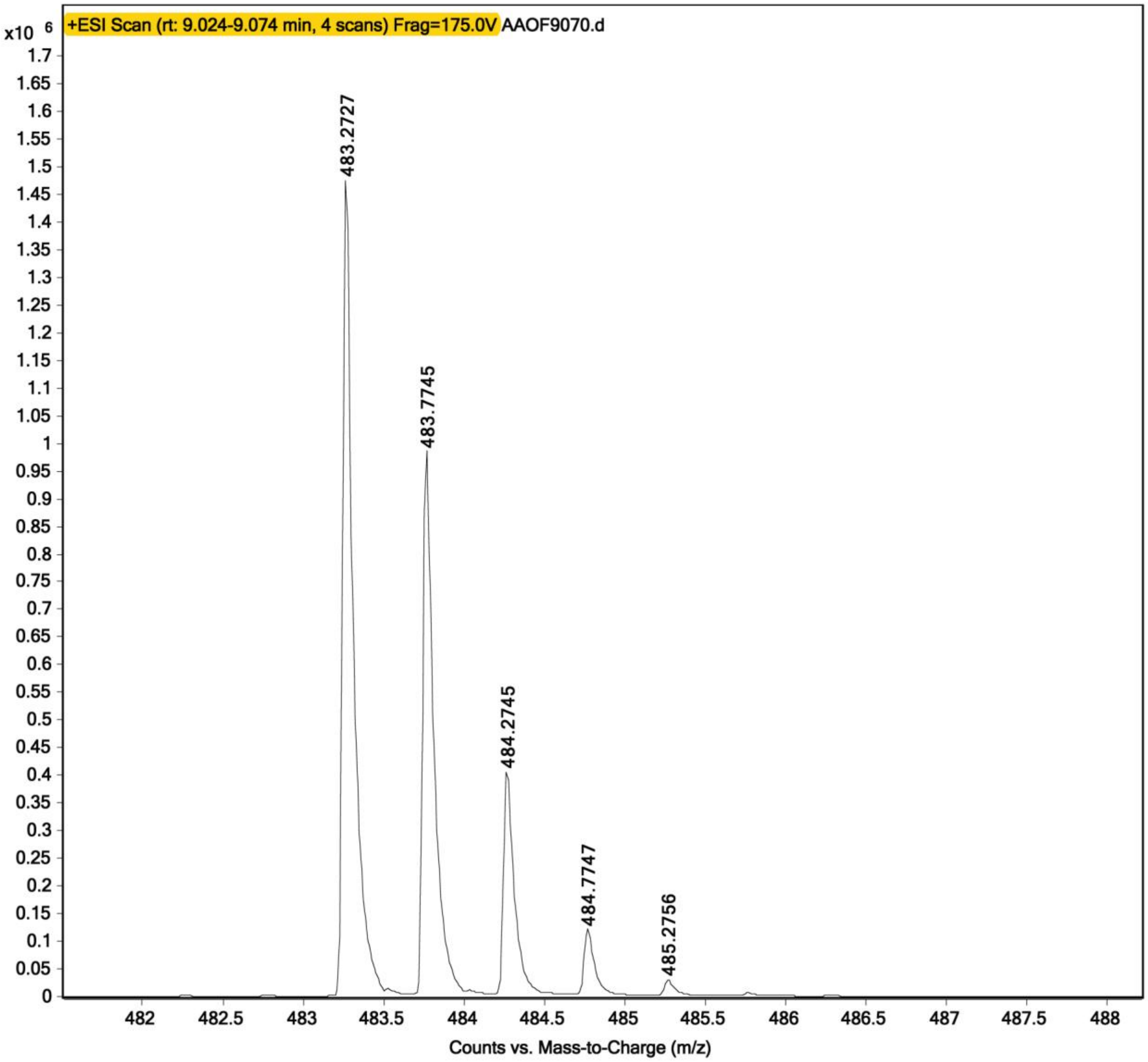

Figure S18. The enlargement of the ESI mass spectrum of the separated HPLC peak region 9.024−9.074 min (see Figure 15A) with the ylide dication ionization species of compound **3**, showing the 0.5 Da isotope peak spacing of the dication $(C_{58}H_{74}N_6O_5S)^{2+}$ *m*/*z* 483.2727 Da.

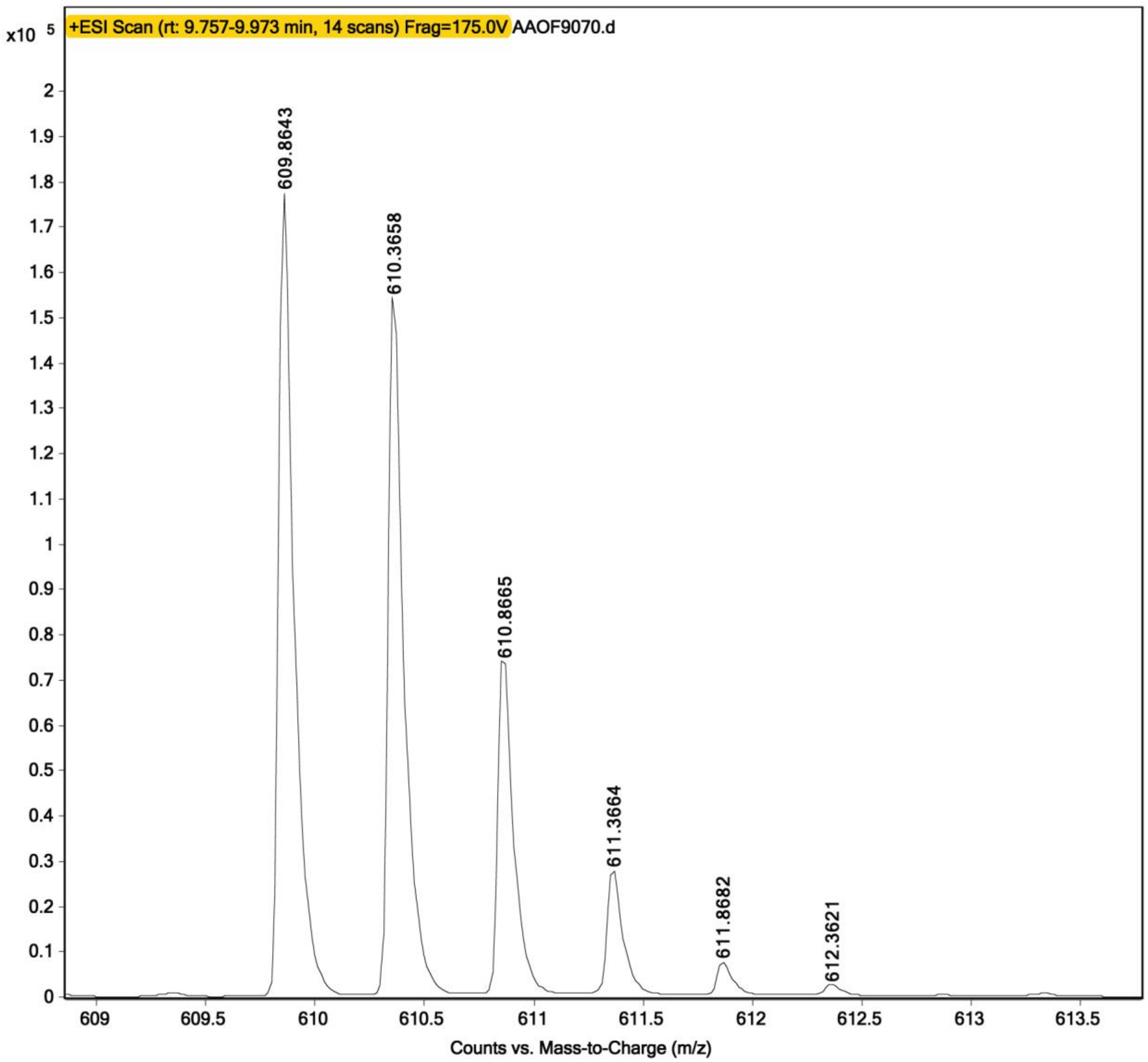

Figure S19. The enlargement of the ESI mass spectrum of the separated HPLC peak region 9.757−9.973 min (see Figure 15A) with the ylide monocation ionization species of compound **3**, showing the 0.5 Da isotope peak spacing of the dication $(C_{74}H_{89}N_7O_7S)^{2+}$ *m*/*z* 609.8643 Da.

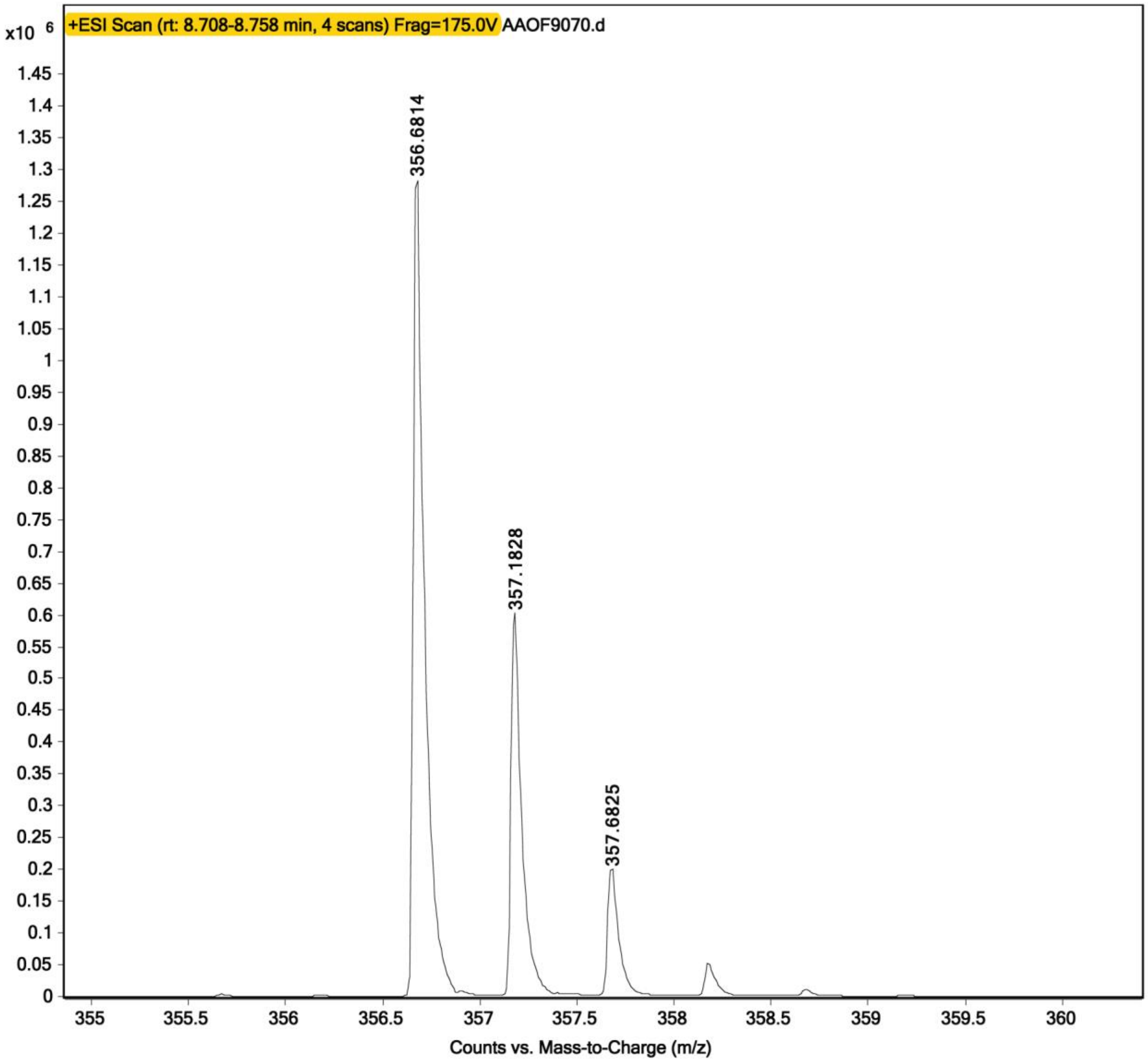

Figure S20. The enlargement of the ESI mass spectrum of the separated HPLC peak region 8.706−8.758 min (see Figure 15A) with the ammonium monocation ionization species of compound **3**, showing the 0.5 Da isotope peak spacing of the dication $(C_{40}H_{49}N_4O_6S)^{2+}$ *m*/*z* 356.6814 Da.

Table S1. National Cancer Institute (NCI) Developmental Therapeutics Program (DTP) 60-cancer cell 5-dose testing of compound **1** (**PT162**), compound **2** (**PT166**), and compound **3** (**PT167**) (with cellular p53 status).

Cancer cell line	Compound	**GI50** (µM)	**TGI** (µM)	**LC50** (µM)
**Leukemia** (p53 status: *, termination; del, deletion; fs, frame shift; Lit., Literature)				
**CCRF-CEM** mutant p53 R175H, R248Q	**1**	1.46	> 50	> 50
	**2**	0.422	> 100	> 100
	**3**	0.904	2.47	14.9
**HL-60 (TB)** mutant p53 p.M1_*394del	**1**	0.87	2.71	> 50
	**2**	0.113	1.26	> 100
	**3**	0.168	1.03	6.85
**K-562** mutant p53 p.Q136fs*13	**1**	1.46	36.2	> 50
	**2**	0.362	> 100	> 100
	**3**	—	—	—
**MOLT-4** mutant p53 Lit. uncertain	**1**	1.32	4.09	> 50
	**2**	0.548	> 100	> 100
	**3**	0.562	1.93	13.3
**RPMI-8226** mutant p53 E285K	**1**	1.32	3.99	> 50
	**2**	0.349	8.65	> 100
	**3**	0.757	1.76	> 33.3
**SR** wild-type p53	**1**	1.49	> 50	> 50
	**2**	0.279	> 100	> 100
	**3**	—	—	—
**Non-small-cell lung cancer** (p53 status: *, termination; del, deletion; fs, frame shift)				
**A549** wild-type p53	**1**	2.13	8.70	24.2
	**2**	3.23	> 100	> 100
	**3**	1.61	6.00	18.2
**EKVX** mutant p53 splicing defect	**1**	—	—	—
	**2**	#	> 100	> 100
	**3**	2.68	8.46	23.6
**HOP-62** mutant p53 splicing defect	**1**	2.66	10.1	29.7
	**2**	0.700	> 100	> 100
	**3**	2.14	7.43	20.2
**HOP-92** mutant p53 R175L	**1**	1.14	3.06	12.6
	**2**	—	—	—
	**3**	0.897	4.08	15.1
**NCI-H226** Lit. inconclusive	**1**	2.76	10.8	31.2
	**2**	1.74	> 100	> 100
	**3**	1.96	9.97	> 33.3
**NCI-H23** mutant p53 M246I	**1**	1.53	6.36	28.2
	**2**	0.438	> 100	> 100
	**3**	1.38	5.33	16.4
**NCI-H322M** mutant p53 R248L	**1**	1.26	5.10	16.0
	**2**	0.619	> 100	> 100
	**3**	2.57	7.03	16.3
**NCI-H460** wild-type p53	**1**	1.19	2.71	17.9
	**2**	0.429	> 100	> 100
	**3**	1.22	3.79	13.5

**NCI-H522**<br>mutant p53<br>p.P191fs*56	**1**	0.731	1.55	3.30
	**2**	0.550	> 100	> 100
	**3**	0.699	1.88	8.33
**Colon cancer** (p53 status: *, termination; del, deletion; fs, frame shift; Lit., Literature)				
**COLO 205**<br>mutant p53<br>Lit. uncertain	**1**	1.09	3.13	24.8
	**2**	0.280	0.622	9.13
	**3**	0.683	1.52	3.61
**HCC-2998**<br>mutant p53<br>R213X	**1**	1.22	5.31	22.6
	**2**	0.272	0.674	> 100
	**3**	0.635	1.47	3.55
**HCT-116**<br>wild-type p53	**1**	0.728	2.01	6.47
	**2**	0.377	10.6	> 100
	**3**	0.896	3.07	11.0
**HCT-15**<br>mutant p53<br>Lit. uncertain	**1**	1.10	3.91	18.4
	**2**	4.03	> 100	> 100
	**3**	1.56	5.96	17.2
**HT29**<br>mutant p53<br>R273H	**1**	0.857	2.82	14.6
	**2**	0.336	> 100	> 100
	**3**	0.958	2.81	11.3
**KM-12**<br>mutant p53<br>Lit. uncertain	**1**	1.65	6.75	20.6
	**2**	0.255	0.801	> 100
	**3**	1.08	4.52	22.5
**SW-620**<br>mutant p53<br>R273H, P309S	**1**	1.25	3.95	17.2
	**2**	0.369	> 100	> 100
	**3**	0.814	2.06	8.74
**CNS cancer (brain tumor)** (p53 status: *, termination; del, deletion; fs, frame shift)				
**SF-268**<br>mutant p53<br>R273H	**1**	1.53	5.41	30.8
	**2**	0.581	> 100	> 100
	**3**	1.37	6.54	29.9
**SF-295**<br>mutant p53<br>R248Q	**1**	3.05	10.4	28.2
	**2**	0.549	> 100	> 100
	**3**	1.53	5.92	15.4
**SF-539**<br>mutant p53<br>Lit. uncertain	**1**	1.01	2.48	7.59
	**2**	0.418	20.5	> 100
	**3**	1.14	4.30	13.8
**SNB-19**<br>mutant p53<br>R273H	**1**	1.92	7.27	19.9
	**2**	0.686	> 100	> 100
	**3**	1.65	6.20	19.5
**SNB-75**<br>mutant p53<br>E258K	**1**	2.34	9.73	27.9
	**2**	0.441	> 100	> 100
	**3**	1.40	6.68	20.1
**U251**<br>mutant p53<br>R273H	**1**	1.12	2.69	8.79
	**2**	0.479	> 100	> 100
	**3**	—	—	—
**Melanoma** (p53 status: *, termination; del, deletion; fs, frame shift; Lit., Literature)				
**LOX IMVI**<br>wild-type p53	**1**	0.505	1.27	3.18
	**2**	0.710	> 100	> 100
	**3**	0.873	2.86	14.3

**MALME-3M**<br>wild-type p53	**1**	0.839	1.86	4.12
	**2**	0.394	> 100	> 100
	**3**	0.830	3.04	14.4
**M14**<br>mutant p53<br>G266E	**1**	0.803	2.08	6.82
	**2**	0.284	0.735	> 100
	**3**	0.822	2.26	9.06
**MDA-MB-435**<br>mutant p53<br>G266E	**1**	0.825	2.11	6.50
	**2**	0.233	0.838	> 100
	**3**	0.779	3.50	12.4
**SK-MEL-2**<br>mutant p53<br>G245S	**1**	1.06	3.20	15.6
	**2**	> 100	> 100	> 100
	**3**	0.861	2.76	12.4
**SK-MEL-28**<br>mutant p53<br>L145R	**1**	1.66	7.91	21.9
	**2**	—	> 100	> 100
	**3**	1.02	4.07	13.6
**SK-MEL-5**<br>wild-type p53	**1**	1.52	6.28	18.6
	**2**	0.373	4.71	> 100
	**3**	0.810	2.25	8.65
**UACC-257**<br>wild-type p53	**1**	3.08	10.8	29.4
	**2**	0.646	> 100	> 100
	**3**	4.51	10.3	23.6
**UACC-62**<br>wild-type p53	**1**	1.16	3.68	14.2
	**2**	0.625	> 100	> 100
	**3**	1.61	6.38	18.0
**Ovarian cancer** (p53 status: *, termination; del, deletion; fs, frame shift; Lit., Literature)				
**IGROV1**<br>mutant p53<br>Lit. uncertain	**1**	0.886	2.34	8.65
	**2**	1.18	> 100	> 100
	**3**	1.45	5.95	20.9
**OVCAR-3**<br>mutant p53<br>R248Q	**1**	1.04	2.82	9.97
	**2**	0.217	0.459	—
	**3**	1.66	6.53	18.5
**OVCAR-4**<br>mutant p53<br>Lit. uncertain	**1**	1.16	5.45	18.4
	**2**	0.507	> 100	> 100
	**3**	2.17	8.94	30.0
**OVCAR-5**<br>Lit. inconclusive	**1**	0.977	2.76	10.8
	**2**	9.67	> 100	> 100
	**3**	1.17	5.03	14.2
**OVCAR-8**<br>mutant p53<br>splicing defect	**1**	2.07	8.48	35.3
	**2**	0.970	> 100	> 100
	**3**	2.03	7.49	24.3
**NCI/ADR-RES**<br>mutant p53<br>splicing defect	**1**	1.44	4.55	> 50
	**2**	25.3	> 100	> 100
	**3**	5.43	12.5	28.6
**SK-OV-3**<br>no p53 protein<br>expressed	**1**	5.11	11.4	25.5
	**2**	1.39	> 100	> 100
	**3**	3.00	7.51	17.6

**Renal cancer** (p53 status: *, termination; del, deletion; fs, frame shift; Lit., Literature)				
**786-0**	**1**	0.848	1.85	4.05
mutant p53	**2**	0.818	> 100	> 100
Lit. uncertain	**3**	1.30	5.28	17.6
**A498**	**1**	1.19	7.05	21.2
wild-type p53	**2**	2.09	> 100	> 100
	**3**	1.07	3.28	12.4
**ACHN**	**1**	0.874	2.14	5.55
wild-type p53	**2**	8.50	> 100	> 100
	**3**	3.68	7.99	17.3
**CAKI-1**	**1**	1.28	4.70	15.4
wild-type p53	**2**	7.12	> 100	> 100
	**3**	3.20	7.37	16.5
**RXF 393**	**1**	0.811	1.56	3.02
mutant p53	**2**	0.298	0.747	> 100
R175H	**3**	1.70	6.65	20.9
**SN12C**	**1**	1.41	4.43	15.8
mutant p53	**2**	0.828	> 100	> 100
E336X	**3**	2.23	6.90	17.4
**TK-10**	**1**	1.85	7.17	21.5
mutant p53	**2**	6.77	> 100	> 100
L264R	**3**	3.66	8.35	19.0
**UO-31**	**1**	0.817	1.96	4.73
wild-type p53	**2**	> 100	> 100	> 100
	**3**	2.16	7.20	18.3
**Prostate cancer** (p53 status: *, termination; del, deletion; fs, frame shift; ¯, null p53 protein)				
**PC-3**	**1**	1.40	5.17	21.4
mutant p53¯	**2**	0.255	> 100	> 100
p.K139fs*31	**3**	1.45	5.64	17.1
**DU-145**	**1**	1.47	4.61	15.2
mutant p53	**2**	0.341	1.44	> 100
P223L, V274F	**3**	2.46	7.10	17.3
**Breast cancer** (p53 status: *, termination; del, deletion; fs, frame shift)				
**MCF7**	**1**	1.07	6.19	45.8
wild-type p53	**2**	0.415	> 100	> 100
	**3**	0.862	3.44	> 33.3
**MDA-MB-231**	**1**	2.09	7.41	22.7
mutant p53	**2**	0.359	1.88	> 100
R280K	**3**	0.994	2.99	12.4
**HS 578T**	**1**	1.92	11.8	> 50
mutant p53	**2**	> 100	> 100	> 100
V157F	**3**	1.22	6.76	> 33.3
**BT-549**	**1**	1.02	2.77	10.2
mutant p53	**2**	0.294	0.924	> 100
R249S	**3**	1.55	5.89	16.5
**T-47D**	**1**	1.34	6.72	38.0
mutant p53	**2**	> 100	> 100	> 100
L194F	**3**	1.83	9.40	> 33.3

**MDA-MB-468** mutant p53 R273H	**1**	0.797	1.68	3.55
	**2**	0.158	0.464	> 100
	**3**	0.657	1.75	10.7
**Mean of Inhibition Data** (**MID**)		**GI50** (μM)	**TGI** (μM)	**LC50** (μM)
$10^{\mathbf{MID}}$	**1**	1.288	4.677	16.596
$10^{\mathbf{MID}}$	**2**	0.933	32.359	95.499
$10^{\mathbf{MID}}$	**3**	1.349	4.571	15.849

#, The **GI50** value could not being calculated due to positive growth.

—, Not tested, because cancer cell line not available at NCI test facility.

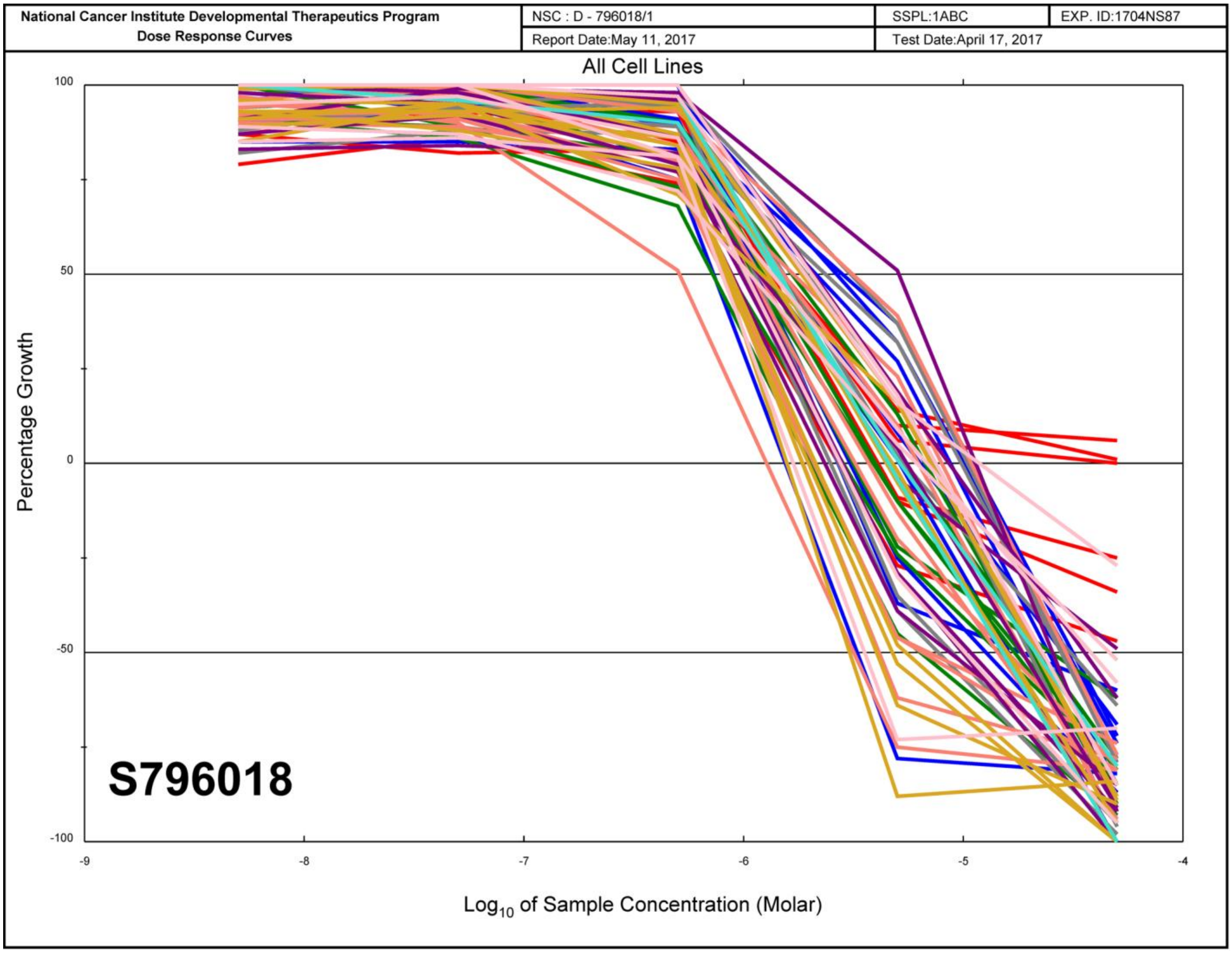

Figure S21. Graphic allover presentation of the antineoplastic activity in the National Cancer Institute (NCI) Developmental Therapeutics Program (DTP) 60-cancer cell 5-dose testing exhibited by the pure drug compound **1** (**PT162**, **NSC 796018**) with **All Cell Lines** in one graphic.

National Cancer Institute Developmental Therapeutics Program
In-Vitro Testing Results

NSC : D - 796018 / 1	Experiment ID : 1704NS87	Test Type : 08	Units : Molar
Report Date : May 11, 2017	Test Date : April 17, 2017	QNS :	MC :
COMI : PT162	Stain Reagent : SRB Dual-Pass Related	SSPL : 1ABC	

			Log10 Concentration													
	Time		Mean Optical Densities					Percent Growth								
Panel/Cell Line	Zero	Ctrl	-8.3	-7.3	-6.3	-5.3	-4.3	-8.3	-7.3	-6.3	-5.3	-4.3	GI50	TGI	LC50
Leukemia															
CCRF-CEM	0.434	1.625	1.618	1.661	1.441	0.557	0.503	99	103	85	10	6	1.46E-6	> 5.00E-5	> 5.00E-5
HL-60(TB)	0.750	2.047	1.769	1.864	1.715	0.548	0.396	79	86	74	-27	-47	8.70E-7	2.71E-6	> 5.00E-5
K-562	0.269	1.764	1.761	1.653	1.593	0.354	0.267	100	93	89	6	.	1.46E-6	3.62E-5	> 5.00E-5
MOLT-4	0.735	2.452	2.235	2.329	2.332	0.670	0.553	87	93	93	-9	-25	1.32E-6	4.09E-6	> 5.00E-5
RPMI-8226	0.587	2.220	2.252	2.299	2.117	0.528	0.386	102	105	94	-10	-34	1.32E-6	3.99E-6	> 5.00E-5
SR	0.441	1.725	1.564	1.491	1.504	0.615	0.457	87	82	83	14	1	1.49E-6	> 5.00E-5	> 5.00E-5
Non-Small Cell Lung Cancer															
A549/ATCC	0.361	1.704	1.693	1.730	1.557	0.724	0.053	99	102	89	27	-85	2.13E-6	8.70E-6	2.42E-5
HOP-62	0.745	2.044	1.904	1.969	2.004	1.165	0.193	89	94	97	32	-74	2.66E-6	1.01E-5	2.97E-5
HOP-92	1.114	1.649	1.626	1.647	1.603	0.837	0.142	96	100	91	-25	-87	1.14E-6	3.06E-6	1.26E-5
NCI-H226	0.841	1.823	1.875	1.867	1.711	1.200	0.233	105	105	89	37	-72	2.76E-6	1.08E-5	3.12E-5
NCI-H23	0.511	1.825	1.804	1.748	1.686	0.618	0.157	98	94	89	8	-69	1.53E-6	6.36E-6	2.82E-5
NCI-H322M	0.874	2.125	1.936	1.933	1.909	0.885	-0.004	85	85	83	1	-100	1.26E-6	5.10E-6	1.60E-5
NCI-H460	0.221	2.164	2.313	2.308	2.213	0.139	0.088	108	107	103	-37	-60	1.19E-6	2.71E-6	1.79E-5
NCI-H522	0.892	2.118	2.215	2.030	1.815	0.200	0.162	108	93	75	-78	-82	7.31E-7	1.55E-6	3.30E-6
Colon Cancer															
COLO 205	0.508	1.801	1.785	1.795	1.634	0.396	0.192	99	99	87	-22	-62	1.09E-6	3.13E-6	2.48E-5
HCC-2998	0.694	2.339	2.238	2.288	2.016	0.729	0.156	94	97	80	2	-78	1.22E-6	5.31E-6	2.26E-5
HCT-116	0.195	1.586	1.457	1.393	1.147	0.108	0.014	91	86	68	-45	-93	7.28E-7	2.01E-6	6.47E-6
HCT-15	0.192	1.407	1.294	1.276	1.176	0.174	0.037	91	89	81	-10	-81	1.10E-6	3.91E-6	1.84E-5
HT29	0.230	1.418	1.436	1.288	1.093	0.175	0.047	102	89	73	-24	-80	8.57E-7	2.82E-6	1.46E-5
KM12	0.508	2.477	2.465	2.492	2.271	0.774	0.052	99	101	90	13	-90	1.65E-6	6.75E-6	2.06E-5
SW-620	0.265	1.779	1.766	1.770	1.625	0.238	0.041	99	99	90	-10	-85	1.25E-6	3.95E-6	1.72E-5
CNS Cancer															
SF-268	0.537	1.916	1.923	1.840	1.847	0.568	0.194	101	94	95	2	-64	1.53E-6	5.41E-6	3.08E-5
SF-295	0.604	2.280	2.086	2.200	2.241	1.223	0.129	88	95	98	37	-79	3.05E-6	1.04E-5	2.82E-5
SF-539	1.012	2.658	2.490	2.556	2.485	0.614	0.018	90	94	89	-39	-98	1.01E-6	2.48E-6	7.59E-6
SNB-19	0.445	1.952	1.950	1.949	1.867	0.726	0.019	100	100	94	19	-96	1.92E-6	7.27E-6	1.99E-5
SNB-75	0.886	1.781	1.618	1.671	1.668	1.169	0.198	82	88	87	32	-78	2.34E-6	9.73E-6	2.79E-5
U251	0.334	1.564	1.609	1.612	1.515	0.216	0.016	104	104	96	-35	-95	1.12E-6	2.69E-6	8.79E-6
Melanoma															
LOX IMVI	0.333	2.352	2.284	2.153	1.354	0.085	0.063	97	90	51	-75	-81	5.05E-7	1.27E-6	3.18E-6
MALME-3M	0.572	1.296	1.261	1.241	1.169	0.217	0.126	95	92	82	-62	-78	8.39E-7	1.86E-6	4.12E-6
M14	0.420	1.538	1.434	1.433	1.258	0.226	0.108	91	91	75	-46	-74	8.03E-7	2.08E-6	6.82E-6
MDA-MB-435	0.592	2.489	2.328	2.357	2.047	0.320	0.114	92	93	77	-46	-81	8.25E-7	2.11E-6	6.50E-6
SK-MEL-2	1.031	2.002	2.045	1.977	1.848	0.822	0.202	104	97	84	-20	-80	1.06E-6	3.20E-6	1.56E-5
SK-MEL-28	0.753	2.281	2.125	2.119	1.974	1.098	0.071	90	89	80	23	-91	1.66E-6	7.91E-6	2.19E-5
SK-MEL-5	0.632	2.957	2.993	2.933	2.653	0.876	0.030	102	99	87	10	-95	1.52E-6	6.28E-6	1.86E-5
UACC-257	1.062	2.071	2.030	2.114	1.997	1.452	0.250	96	104	93	39	-77	3.08E-6	1.08E-5	2.94E-5
UACC-62	0.631	2.480	2.374	2.437	2.232	0.548	0.037	94	98	87	-13	-94	1.16E-6	3.68E-6	1.42E-5
Ovarian Cancer															
IGROV1	0.375	1.766	1.742	1.690	1.480	0.229	0.055	98	95	79	-39	-85	8.86E-7	2.34E-6	8.65E-6
OVCAR-3	0.417	1.444	1.440	1.423	1.309	0.298	0.001	100	98	87	-29	-100	1.04E-6	2.82E-6	9.97E-6
OVCAR-4	0.826	1.689	1.578	1.616	1.489	0.857	0.073	87	92	77	4	-91	1.16E-6	5.45E-6	1.84E-5
OVCAR-5	0.581	1.404	1.263	1.273	1.259	0.414	0.045	83	84	82	-29	-92	9.77E-7	2.76E-6	1.08E-5
OVCAR-8	0.581	2.270	2.357	2.368	2.279	0.894	0.220	105	106	101	19	-62	2.07E-6	8.48E-6	3.53E-5
NCI/ADR-RES	0.487	1.643	1.650	1.641	1.596	0.467	0.251	101	100	96	-4	-49	1.44E-6	4.55E-6	> 5.00E-5
SK-OV-3	0.894	1.876	1.787	1.867	1.854	1.399	0.071	91	99	98	51	-92	5.11E-6	1.14E-5	2.55E-5
Renal Cancer															
786-0	0.403	1.759	1.632	1.687	1.540	0.147	0.041	91	95	84	-64	-90	8.48E-7	1.85E-6	4.05E-6
A498	1.422	2.245	2.125	2.205	2.005	1.551	0.159	85	95	71	16	-89	1.19E-6	7.05E-6	2.12E-5
ACHN	0.379	1.642	1.589	1.581	1.405	0.199	-0.002	96	95	81	-48	-100	8.74E-7	2.14E-6	5.55E-6
CAKI-1	0.767	2.753	2.532	2.597	2.481	0.749	0.004	89	92	86	-2	-100	1.28E-6	4.70E-6	1.54E-5
RXF 393	0.685	1.323	1.358	1.334	1.239	0.080	0.112	105	102	87	-88	-84	8.11E-7	1.56E-6	3.02E-6
SN12C	0.391	1.784	1.773	1.781	1.719	0.371	0.020	99	100	95	-5	-95	1.41E-6	4.43E-6	1.58E-5
TK-10	0.865	1.676	1.618	1.621	1.630	0.998	0.100	93	93	94	16	-88	1.85E-6	7.17E-6	2.15E-5
UO-31	0.608	2.142	2.029	1.962	1.804	0.285	-0.007	93	88	78	-53	-100	8.17E-7	1.96E-6	4.73E-6
Prostate Cancer															
PC-3	0.440	1.517	1.515	1.477	1.406	0.453	0.089	100	96	90	1	-80	1.40E-6	5.17E-6	2.14E-5
DU-145	0.338	1.467	1.493	1.480	1.437	0.326	-0.002	102	101	97	-4	-100	1.47E-6	4.61E-6	1.52E-5
Breast Cancer															
MCF7	0.346	2.158	1.966	1.929	1.650	0.443	0.166	89	87	72	5	-52	1.07E-6	6.19E-6	4.58E-5
MDA-MB-231/ATCC	0.547	1.458	1.511	1.536	1.487	0.707	0.081	106	108	103	18	-85	2.09E-6	7.41E-6	2.27E-5
HS 578T	0.815	1.655	1.671	1.681	1.631	0.952	0.593	102	103	97	16	-27	1.92E-6	1.18E-5	> 5.00E-5
BT-549	1.139	2.003	1.962	1.978	1.880	0.803	0.053	95	97	86	-30	-95	1.02E-6	2.77E-6	1.02E-5
T-47D	0.703	1.599	1.461	1.471	1.429	0.780	0.296	85	86	81	9	-58	1.34E-6	6.72E-6	3.80E-5
MDA-MB-468	0.640	1.327	1.331	1.332	1.198	0.174	0.190	101	101	81	-73	-70	7.97E-7	1.68E-6	3.55E-6

Figure S22. Tabular list of the antineoplastic activity exhibited by the pure drug compound **1** (**PT162**, **NSC 796018**) in the National Cancer Institute (NCI) Developmental Therapeutics Program (DTP) 60-cancer cell 5-dose testing with the Mean Optical Densities of the used vital stain sulforhodamine B (SRB) retained in the cells given. **Percent Growth**, **GI50** (**Growth Inhibition 50%**), **TGI** (**Total Growth Inhibition = 0% Growth**), and **LC50** (**Lethal Concentration 50% = –50% Growth**) are given in linear concentration units**.**

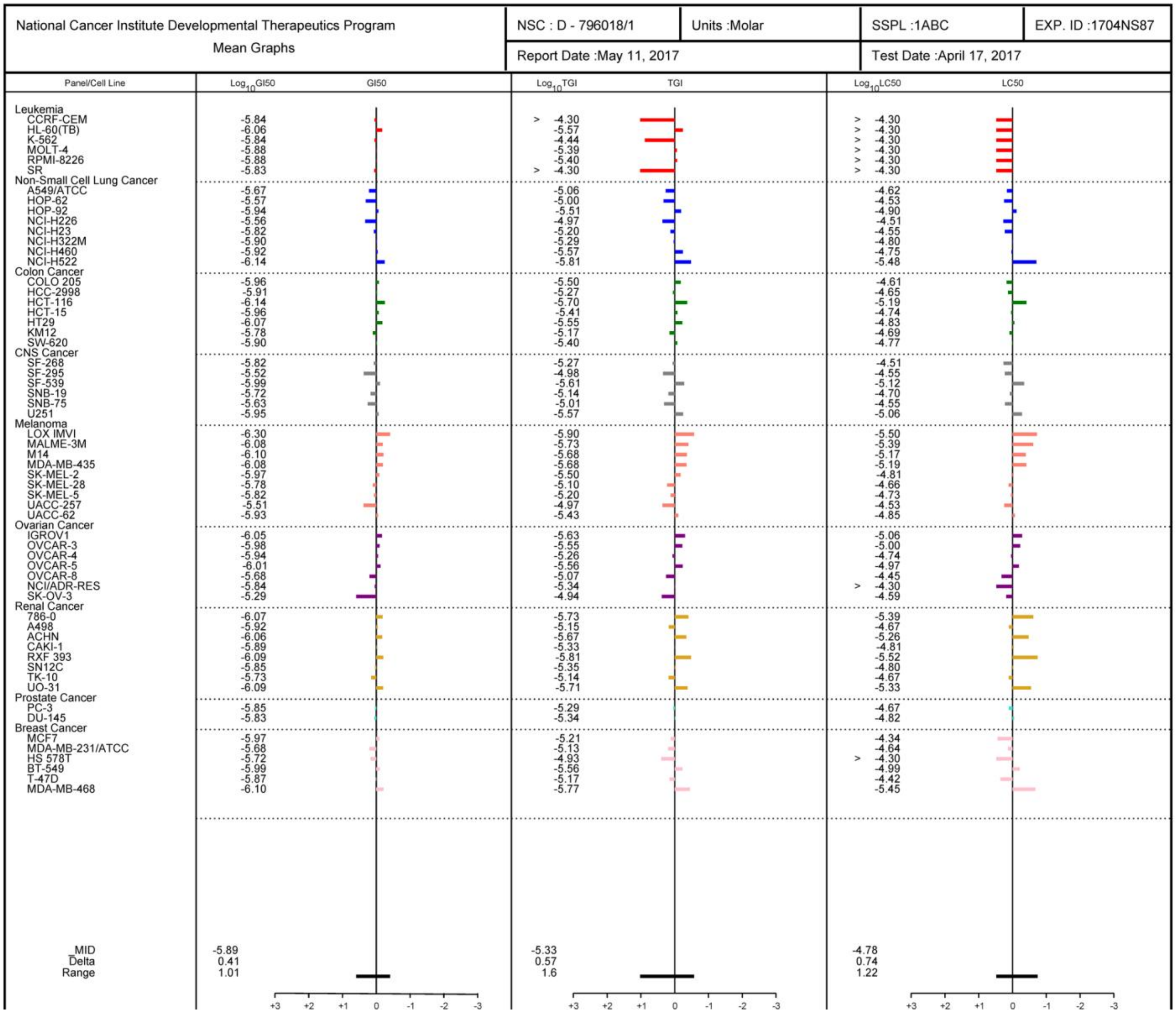

Figure S23. Graphic table presentation of the antineoplastic activity exhibited by the pure drug compound **1** (**PT162**, **NSC 796018**) in the National Cancer Institute (NCI) Developmental Therapeutics Program (DTP) 60-cancer cell 5-dose testing with the **Percent Growth**, **GI50** (**Growth Inhibition 50%**), **TGI** (**Total Growth Inhibition = 0% Growth**), and **LC50** (**Lethal Concentration 50% = –50% Growth**) given in linear concentration units. This is the summary of the results expressed in log_{10} units. The defined tumor inhibition criterion (**GI50**, **TGI**, or **LC50**) is recalculated in log_{10} expression, and the colored bars which indicate then the sensitivity of the individual tumor cell line to the agent are given, but in log_{10} units. **Bar to the left: less sensitive**

than mean; **Bar to the right: more sensitive than mean**. The most important feature is at the bottom of the page: the **MID** (**Mean of Inhibition Data**) indicates the mean concentration for all tested cell lines required for the drug to reach the defined tumor inhibition criterion (**GI50**, **TGI**, or **LC50**). It is given in **$\mathbf{Log_{10}}$GI50**, **$\mathbf{Log_{10}}$TGI** and **$\mathbf{Log_{10}}$LC50**. From that values the corresponding **MID** is calculated. The more negative the **MID**, the more potent is the drug. The **MID** can be transformed from logarithmic into linear concentrations by the formula: $c = 10^{\mathbf{MID}}$.

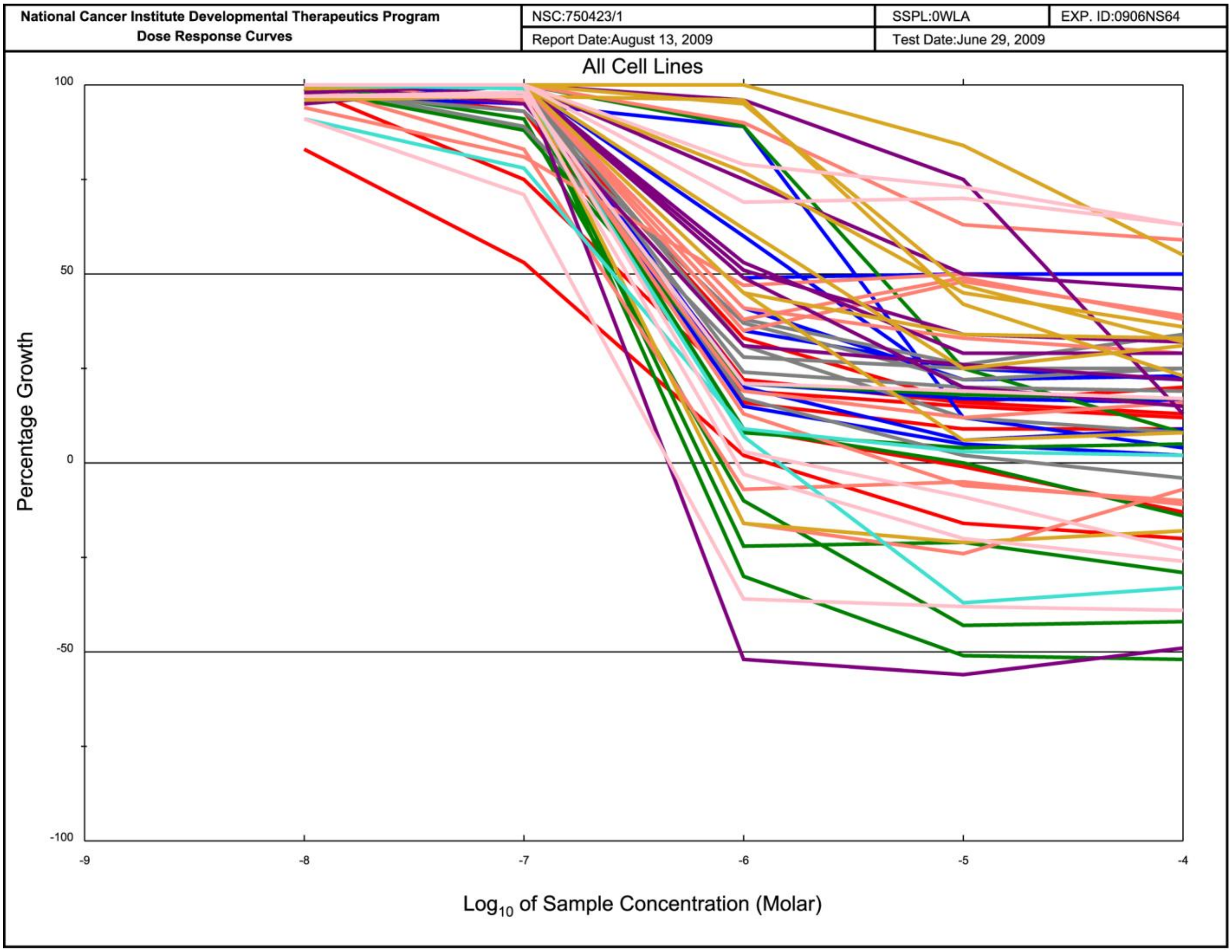

Figure S24. Graphic allover presentation of the antineoplastic activity in the National Cancer Institute (NCI) Developmental Therapeutics Program (DTP) 60-cancer cell 5-dose testing exhibited by the drug compound **2** (**PT166**, **NSC 750423**) with **All Cell Lines** in one graphic.

National Cancer Institute Developmental Therapeutics Program
In-Vitro Testing Results

NSC : 750423 / 1	Experiment ID : 0906NS64	Test Type : 08	Units : Molar
Report Date : August 13, 2009	Test Date : June 29, 2009	QNS :	MC :
COMI : colch-TSC (84886)	Stain Reagent : SRB Dual-Pass Related	SSPL : 0WLA	

			Log10 Concentration												
	Time		Mean Optical Densities					Percent Growth							
Panel/Cell Line	Zero	Ctrl	-8.0	-7.0	-6.0	-5.0	-4.0	-8.0	-7.0	-6.0	-5.0	-4.0	GI50	TGI	LC50
**Leukemia**															
CCRF-CEM	0.356	1.223	1.293	1.199	0.545	0.496	0.531	108	97	22	16	20	4.22E-7	> 1.00E-4	> 1.00E-4
HL-60(TB)	0.825	2.422	2.153	1.668	0.854	0.694	0.664	83	53	2	-16	-20	1.13E-7	1.26E-6	> 1.00E-4
K-562	0.287	1.715	1.711	1.622	0.510	0.417	0.422	100	93	16	9	9	3.62E-7	> 1.00E-4	> 1.00E-4
MOLT-4	0.609	1.986	2.143	1.948	1.068	0.825	0.792	111	97	33	16	13	5.48E-7	> 1.00E-4	> 1.00E-4
RPMI-8226	0.864	1.665	1.739	1.658	0.933	0.859	0.751	109	99	9	-1	-13	3.49E-7	8.65E-6	> 1.00E-4
SR	0.312	1.029	1.020	0.850	0.449	0.417	0.400	99	75	19	15	12	2.79E-7	> 1.00E-4	> 1.00E-4
**Non-Small Cell Lung Cancer**															
A549/ATCC	0.337	1.197	1.167	1.154	1.102	0.444	0.375	97	95	89	12	4	3.23E-6	> 1.00E-4	> 1.00E-4
EKVX	0.663	1.690	1.653	1.702	1.171	1.172	1.182	96	101	49	50	50	.	> 1.00E-4	> 1.00E-4
HOP-62	0.341	1.030	1.041	1.039	0.621	0.495	0.496	102	101	41	22	23	7.00E-7	> 1.00E-4	> 1.00E-4
NCI-H226	0.619	1.115	1.148	1.182	0.915	0.716	0.699	107	113	60	19	16	1.74E-6	> 1.00E-4	> 1.00E-4
NCI-H23	0.352	0.990	1.015	1.018	0.477	0.391	0.412	104	104	20	6	9	4.38E-7	> 1.00E-4	> 1.00E-4
NCI-H322M	0.533	1.061	1.061	1.090	0.720	0.665	0.649	100	106	35	25	22	6.19E-7	> 1.00E-4	> 1.00E-4
NCI-H460	0.117	1.142	1.287	1.240	0.275	0.165	0.138	114	110	15	5	2	4.29E-7	> 1.00E-4	> 1.00E-4
NCI-H522	0.482	1.412	1.668	1.706	0.682	0.639	0.633	127	132	21	17	16	5.50E-7	> 1.00E-4	> 1.00E-4
**Colon Cancer**															
COLO 205	0.180	0.657	0.737	0.727	0.127	0.089	0.087	117	115	-30	-51	-52	2.80E-7	6.22E-7	9.13E-6
HCC-2998	0.536	1.610	1.614	1.667	0.420	0.424	0.382	100	105	-22	-21	-29	2.72E-7	6.74E-7	> 1.00E-4
HCT-116	0.147	1.082	1.131	1.140	0.229	0.151	0.127	105	106	9	.	-14	3.77E-7	1.06E-5	> 1.00E-4
HCT-15	0.284	1.537	1.587	1.568	1.394	0.595	0.380	104	102	89	25	8	4.03E-6	> 1.00E-4	> 1.00E-4
HT29	0.209	1.360	1.338	1.328	0.296	0.259	0.262	98	97	8	4	5	3.36E-7	> 1.00E-4	> 1.00E-4
KM12	0.237	1.196	1.231	1.110	0.214	0.135	0.138	104	91	-10	-43	-42	2.55E-7	8.01E-7	> 1.00E-4
SW-620	0.160	1.104	1.096	0.995	0.355	0.328	0.321	99	88	21	18	17	3.69E-7	> 1.00E-4	> 1.00E-4
**CNS Cancer**															
SF-268	0.444	1.433	1.412	1.360	0.809	0.660	0.693	98	93	37	22	25	5.81E-7	> 1.00E-4	> 1.00E-4
SF-295	0.687	2.195	2.253	2.266	1.151	0.869	0.812	104	105	31	12	8	5.49E-7	> 1.00E-4	> 1.00E-4
SF-539	0.373	1.398	1.401	1.442	0.546	0.393	0.357	100	104	17	2	-4	4.18E-7	2.05E-5	> 1.00E-4
SNB-19	0.607	1.561	1.625	1.665	0.971	0.852	0.928	107	111	38	26	34	6.86E-7	> 1.00E-4	> 1.00E-4
SNB-75	0.502	0.959	0.968	0.908	0.632	0.617	0.617	102	89	28	25	25	4.41E-7	> 1.00E-4	> 1.00E-4
U251	0.207	1.094	1.088	1.141	0.421	0.388	0.380	99	105	24	20	19	4.79E-7	> 1.00E-4	> 1.00E-4
**Melanoma**															
LOX IMVI	0.261	1.596	1.631	1.653	0.802	0.706	0.650	103	104	41	33	29	7.10E-7	> 1.00E-4	> 1.00E-4
MALME-3M	0.417	0.566	0.574	0.560	0.445	0.436	0.442	106	96	19	12	16	3.94E-7	> 1.00E-4	> 1.00E-4
M14	0.317	1.044	1.086	1.080	0.266	0.240	0.294	106	105	-16	-24	-7	2.84E-7	7.35E-7	> 1.00E-4
MDA-MB-435	0.370	1.324	1.361	1.163	0.345	0.351	0.329	104	83	-7	-5	-11	2.33E-7	8.38E-7	> 1.00E-4
SK-MEL-2	0.484	0.922	0.992	1.029	0.879	0.758	0.740	116	125	90	63	59	> 1.00E-4	> 1.00E-4	> 1.00E-4
SK-MEL-28	0.262	0.870	0.832	0.754	0.546	0.569	0.543	94	81	47	50	46	.	> 1.00E-4	> 1.00E-4
SK-MEL-5	0.571	2.052	2.059	2.045	0.764	0.535	0.514	100	99	13	-6	-10	3.73E-7	4.71E-6	> 1.00E-4
UACC-257	0.448	0.815	0.816	0.818	0.588	0.629	0.589	100	101	38	49	38	6.46E-7	> 1.00E-4	> 1.00E-4
UACC-62	0.821	2.155	2.248	2.246	1.294	1.457	1.338	107	107	35	48	39	6.25E-7	> 1.00E-4	> 1.00E-4
**Ovarian Cancer**															
IGROV1	0.360	1.694	1.624	1.727	1.043	0.815	0.784	95	102	51	34	32	1.18E-6	> 1.00E-4	> 1.00E-4
OVCAR-3	0.320	0.943	0.965	0.952	0.154	0.140	0.165	104	101	-52	-56	-49	2.17E-7	4.59E-7	.
OVCAR-4	0.463	1.261	1.315	1.219	0.713	0.672	0.635	107	95	31	26	22	5.07E-7	> 1.00E-4	> 1.00E-4
OVCAR-5	0.348	0.719	0.699	0.720	0.626	0.532	0.518	95	100	75	50	46	9.67E-6	> 1.00E-4	> 1.00E-4
OVCAR-8	0.507	1.475	1.514	1.542	0.984	0.702	0.650	104	107	49	20	15	9.70E-7	> 1.00E-4	> 1.00E-4
NCI/ADR-RES	0.222	0.710	0.702	0.714	0.690	0.589	0.284	98	101	96	75	13	2.53E-5	> 1.00E-4	> 1.00E-4
SK-OV-3	0.510	1.213	1.385	1.453	0.886	0.715	0.712	124	134	53	29	29	1.39E-6	> 1.00E-4	> 1.00E-4
**Renal Cancer**															
786-0	0.371	1.447	1.471	1.491	0.854	0.440	0.461	102	104	45	6	8	8.18E-7	> 1.00E-4	> 1.00E-4
A498	0.688	1.527	1.621	1.608	1.207	0.897	0.949	111	110	62	25	31	2.09E-6	> 1.00E-4	> 1.00E-4
ACHN	0.320	1.117	1.222	1.221	1.076	0.692	0.574	113	113	95	47	32	8.50E-6	> 1.00E-4	> 1.00E-4
CAKI-1	0.659	1.875	1.821	1.835	1.822	1.171	0.942	96	97	96	42	23	7.12E-6	> 1.00E-4	> 1.00E-4
RXF 393	0.650	0.840	0.858	0.858	0.547	0.514	0.535	110	109	-16	-21	-18	2.98E-7	7.47E-7	> 1.00E-4
SN12C	0.370	1.309	1.301	1.362	0.793	0.691	0.678	99	106	45	34	33	8.28E-7	> 1.00E-4	> 1.00E-4
TK-10	0.504	0.957	1.011	1.036	0.852	0.706	0.669	112	117	77	45	36	6.77E-6	> 1.00E-4	> 1.00E-4
UO-31	0.645	1.192	1.278	1.279	1.251	1.105	0.944	116	116	111	84	55	> 1.00E-4	> 1.00E-4	> 1.00E-4
**Prostate Cancer**															
PC-3	1.013	2.121	2.023	1.875	1.118	1.048	1.039	91	78	9	3	2	2.55E-7	> 1.00E-4	> 1.00E-4
DU-145	0.296	1.074	1.079	1.068	0.350	0.187	0.199	101	99	7	-37	-33	3.41E-7	1.44E-6	> 1.00E-4
**Breast Cancer**															
MCF7	0.241	1.345	1.312	1.320	0.468	0.447	0.434	97	98	21	19	17	4.15E-7	> 1.00E-4	> 1.00E-4
MDA-MB-231/ATCC	0.446	0.922	0.990	0.962	0.462	0.408	0.342	114	108	3	-9	-23	3.59E-7	1.88E-6	> 1.00E-4
HS 578T	0.568	1.728	1.974	1.959	1.484	1.418	1.299	121	120	79	73	63	> 1.00E-4	> 1.00E-4	> 1.00E-4
BT-549	0.938	1.523	1.506	1.506	0.906	0.749	0.698	97	97	-3	-20	-26	2.94E-7	9.24E-7	> 1.00E-4
T-47D	0.646	1.231	1.240	1.234	1.047	1.054	1.012	102	101	69	70	63	> 1.00E-4	> 1.00E-4	> 1.00E-4
MDA-MB-468	0.476	1.315	1.241	1.074	0.306	0.295	0.289	91	71	-36	-38	-39	1.58E-7	4.64E-7	> 1.00E-4

Figure S25. Tabular list of the antineoplastic activity exhibited by the drug compound **2** (**PT166**, **NSC 750423**) in the National Cancer Institute (NCI) Developmental Therapeutics Program (DTP) 60-cancer cell 5-dose testing with the Mean Optical Densities of the used vital stain sulforhodamine B (SRB) retained in the cells given. **Percent Growth**, **GI50** (**Growth Inhibition 50%**), **TGI** (**Total Growth Inhibition = 0% Growth**), and **LC50** (**Lethal Concentration 50% = –50% Growth**) are given in linear concentration units.

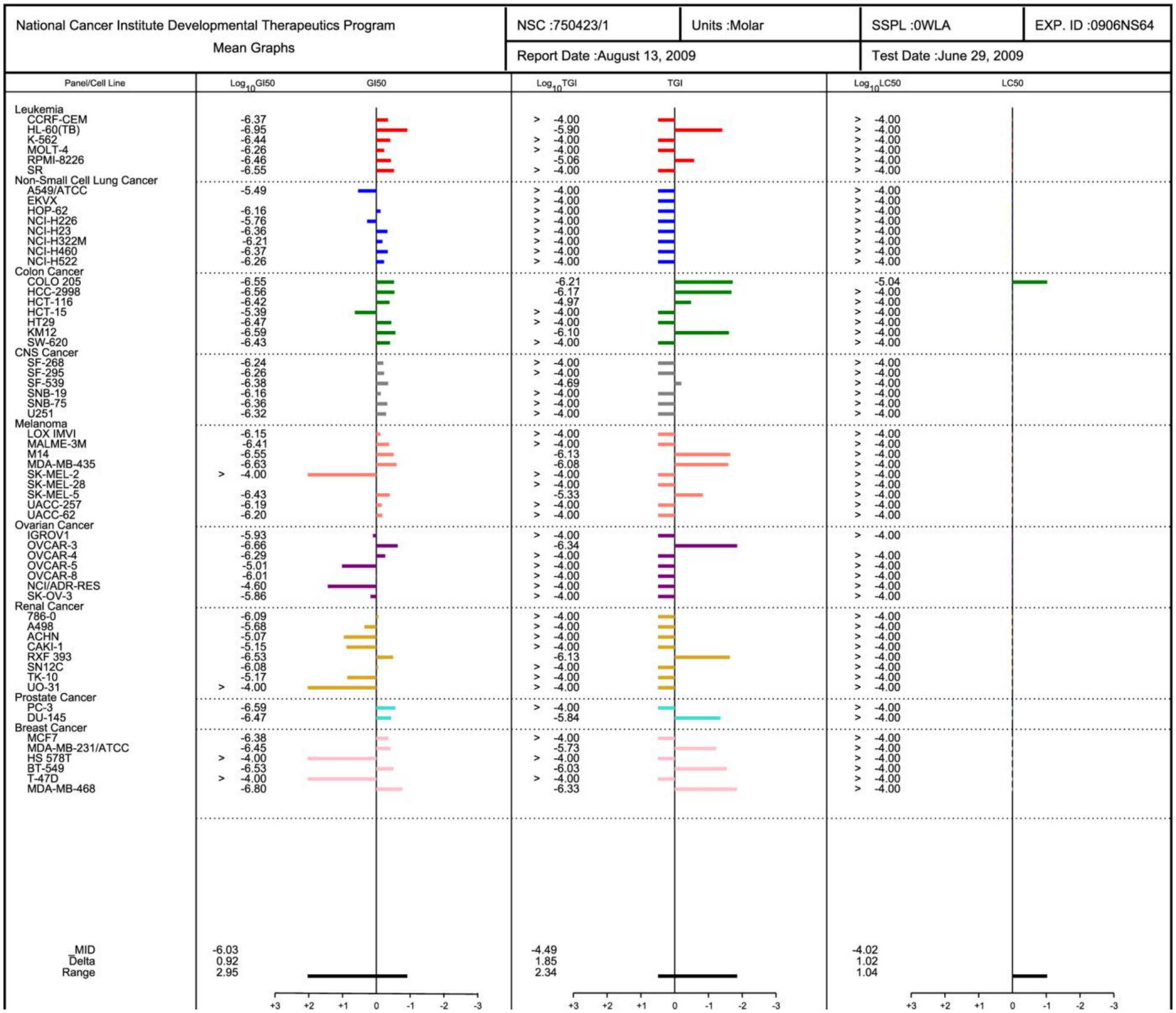

Figure S26. Graphic table presentation of the antineoplastic activity exhibited by the drug compound **2** (**PT166**, **NSC 750423**) in the National Cancer Institute (NCI) Developmental Therapeutics Program (DTP) 60-cancer cell 5-dose testing with the **Percent Growth**, **GI50** (**Growth Inhibition 50%**), **TGI** (**Total Growth Inhibition = 0% Growth**), and **LC50** (**Lethal Concentration 50% = –50% Growth**) given in linear concentration units. This is the summary of the results expressed in log_{10} units. The defined tumor inhibition criterion (**GI50**, **TGI**, or **LC50**) is recalculated in log_{10} expression, and the colored bars which indicate then the sensitivity of the individual tumor cell line to the agent are given, but in log_{10} units. **Bar to the left: less sensitive**

than mean; **Bar to the right: more sensitive than mean**. The most important feature is at the bottom of the page: the **MID** (**Mean of Inhibition Data**) indicates the mean concentration for all tested cell lines required for the drug to reach the defined tumor inhibition criterion (**GI50**, **TGI**, or **LC50**). It is given in **Log_{10}GI50**, **Log_{10}TGI** and **Log_{10}LC50**. From that values the corresponding **MID** is calculated. The more negative the **MID**, the more potent is the drug. The **MID** can be transformed from logarithmic into linear concentrations by the formula: $c = 10^{MID}$.

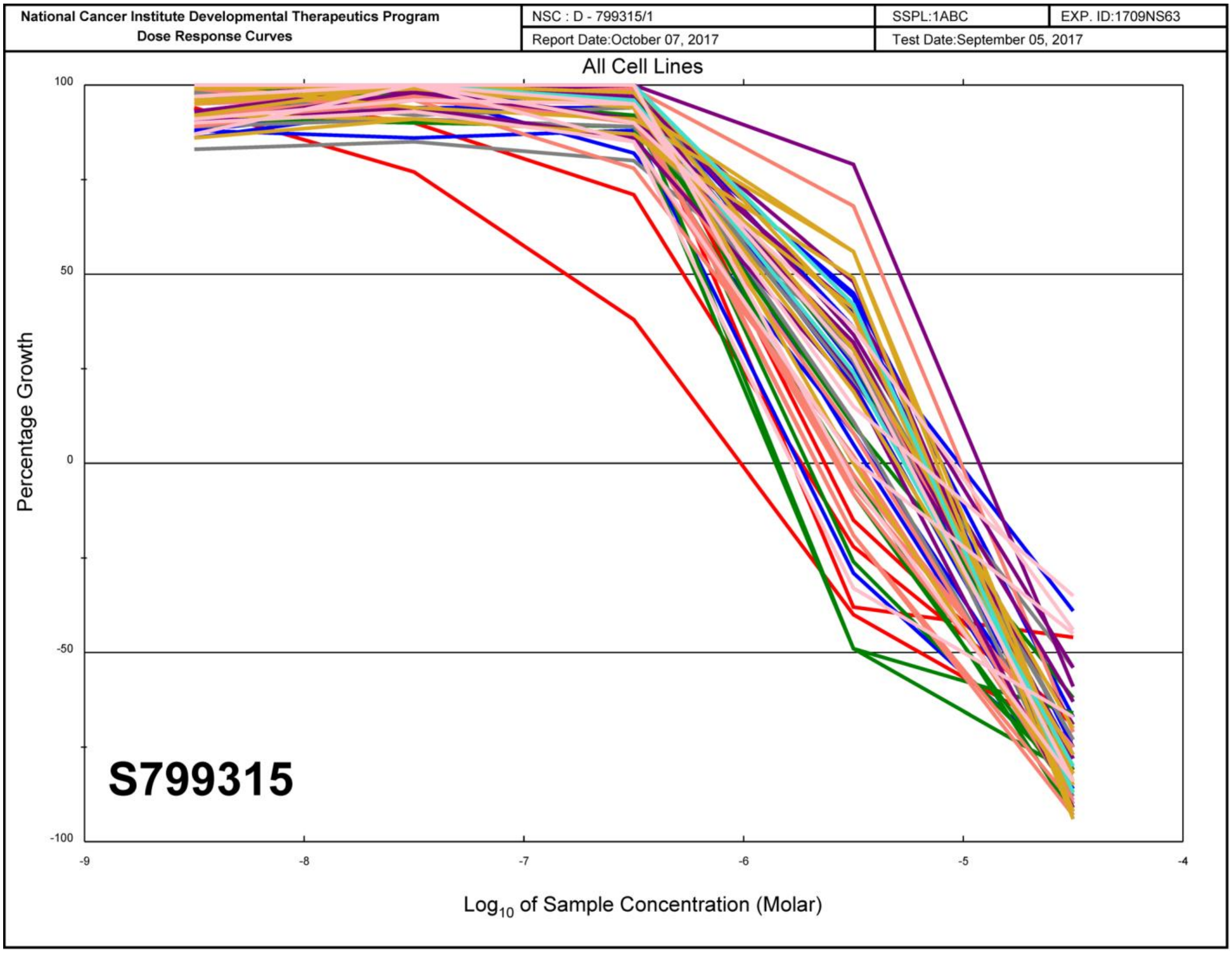

Figure S27. Graphic allover presentation of the antineoplastic activity in the National Cancer Institute (NCI) Developmental Therapeutics Program (DTP) 60-cancer cell 5-dose testing exhibited by the drug compound **3** (**PT167**, **NSC 799315**) with **All Cell Lines** in one graphic.

National Cancer Institute Developmental Therapeutics Program
In-Vitro Testing Results

NSC : D - 799315 / 1	Experiment ID : 1709NS63	Test Type : 08	Units : Molar
Report Date : October 07, 2017	Test Date : September 05, 2017	QNS :	MC :
COMI : PT167	Stain Reagent : SRB Dual-Pass Related	SSPL : 1ABC	

			Log10 Concentration												
	Time		Mean Optical Densities					Percent Growth							
Panel/Cell Line	Zero	Ctrl	-8.5	-7.5	-6.5	-5.5	-4.5	-8.5	-7.5	-6.5	-5.5	-4.5	GI50	TGI	LC50
Leukemia															
CCRF-CEM	0.461	2.449	2.482	2.556	2.443	0.393	0.144	102	105	100	-15	-69	9.04E-7	2.47E-6	1.49E-5
HL-60(TB)	0.857	3.020	2.897	2.528	1.689	0.517	0.236	94	77	38	-40	-73	1.68E-7	1.03E-6	6.85E-6
MOLT-4	0.638	2.703	2.656	2.504	2.108	0.498	0.201	98	90	71	-22	-69	5.62E-7	1.93E-6	1.33E-5
RPMI-8226	0.451	1.817	1.889	1.916	1.799	0.281	0.242	105	107	99	-38	-46	7.57E-7	1.76E-6	> 3.33E-5
Non-Small Cell Lung Cancer															
A549/ATCC	0.341	1.522	1.533	1.524	1.532	0.654	0.078	101	100	101	26	-77	1.61E-6	6.00E-6	1.82E-5
EKVX	0.693	2.180	2.008	2.089	2.090	1.368	0.231	88	94	94	45	-67	2.68E-6	8.46E-6	2.36E-5
HOP-62	0.633	1.719	1.565	1.652	1.632	1.068	0.159	86	94	92	40	-75	2.14E-6	7.43E-6	2.02E-5
HOP-92	1.136	1.750	1.704	1.738	1.639	1.184	0.223	93	98	82	8	-80	8.97E-7	4.08E-6	1.51E-5
NCI-H226	0.603	1.414	1.429	1.431	1.398	0.893	0.366	102	102	98	36	-39	1.96E-6	9.97E-6	> 3.33E-5
NCI-H23	0.495	1.655	1.654	1.628	1.620	0.739	0.091	100	98	97	21	-82	1.38E-6	5.33E-6	1.64E-5
NCI-H322M	0.700	1.763	1.748	1.716	1.717	1.170	0.055	99	96	96	44	-92	2.57E-6	7.03E-6	1.63E-5
NCI-H460	0.246	1.834	1.927	1.994	1.961	0.327	0.036	106	110	108	5	-86	1.22E-6	3.79E-6	1.35E-5
NCI-H522	0.948	2.248	2.098	2.062	2.086	0.673	0.174	88	86	88	-29	-82	6.99E-7	1.88E-6	8.33E-6
Colon Cancer															
COLO 205	0.433	1.448	1.425	1.472	1.398	0.219	0.147	98	102	95	-49	-66	6.83E-7	1.52E-6	3.61E-6
HCC-2998	0.479	1.564	1.468	1.450	1.440	0.244	0.085	91	90	89	-49	-82	6.35E-7	1.47E-6	3.55E-6
HCT-116	0.152	1.300	1.211	1.263	1.187	0.147	0.010	92	97	90	-3	-93	8.96E-7	3.07E-6	1.10E-5
HCT-15	0.218	1.575	1.470	1.543	1.523	0.590	0.042	92	98	96	27	-81	1.56E-6	5.96E-6	1.72E-5
HT29	0.184	0.975	0.982	0.980	0.968	0.170	0.023	101	101	99	-8	-88	9.58E-7	2.81E-6	1.13E-5
KM12	0.366	1.900	1.874	1.856	1.782	0.513	0.138	98	97	92	10	-62	1.08E-6	4.52E-6	2.25E-5
SW-620	0.245	1.570	1.638	1.631	1.544	0.182	0.040	105	105	98	-26	-84	8.14E-7	2.06E-6	8.74E-6
CNS Cancer															
SF-268	0.648	2.073	2.041	1.963	1.990	0.965	0.301	98	92	94	22	-54	1.37E-6	6.54E-6	2.99E-5
SF-295	0.787	2.563	2.370	2.402	2.368	1.323	0.075	89	91	89	30	-90	1.53E-6	5.92E-6	1.54E-5
SF-539	0.784	2.356	2.243	2.299	2.275	0.956	0.097	93	96	95	11	-88	1.14E-6	4.30E-6	1.38E-5
SNB-19	0.422	1.532	1.536	1.626	1.553	0.723	0.113	100	108	102	27	-73	1.65E-6	6.20E-6	1.95E-5
SNB-75	0.874	1.816	1.657	1.677	1.631	1.172	0.237	83	85	80	32	-73	1.40E-6	6.68E-6	2.01E-5
Melanoma															
LOX IMVI	0.360	2.108	2.017	2.054	1.943	0.337	0.089	95	97	91	-6	-75	8.73E-7	2.86E-6	1.43E-5
MALME-3M	0.535	1.269	1.265	1.218	1.160	0.517	0.125	99	93	85	-3	-77	8.30E-7	3.04E-6	1.44E-5
M14	0.339	1.194	1.148	1.183	1.148	0.275	0.033	95	99	95	-19	-90	8.22E-7	2.26E-6	9.06E-6
MDA-MB-435	0.461	2.023	1.847	1.965	1.680	0.493	0.051	89	96	78	2	-89	7.79E-7	3.50E-6	1.24E-5
SK-MEL-2	0.961	2.171	2.118	2.127	2.059	0.884	0.177	96	96	91	-8	-82	8.61E-7	2.76E-6	1.24E-5
SK-MEL-28	0.593	1.761	1.665	1.727	1.641	0.690	0.077	92	97	90	8	-87	1.02E-6	4.07E-6	1.36E-5
SK-MEL-5	0.640	2.822	2.761	2.828	2.681	0.517	0.043	97	100	94	-19	-93	8.10E-7	2.25E-6	8.65E-6
UACC-257	1.116	2.133	2.086	2.182	2.119	1.811	0.327	95	105	99	68	-71	4.51E-6	1.03E-5	2.36E-5
UACC-62	0.845	2.428	2.362	2.385	2.274	1.342	0.171	96	97	90	31	-80	1.61E-6	6.38E-6	1.80E-5
Ovarian Cancer															
IGROV1	0.536	1.959	2.009	1.995	1.925	0.865	0.169	103	103	98	23	-69	1.45E-6	5.95E-6	2.09E-5
OVCAR-3	0.498	1.651	1.697	1.643	1.547	0.870	0.110	104	99	91	32	-78	1.66E-6	6.53E-6	1.85E-5
OVCAR-4	0.624	1.245	1.185	1.232	1.188	0.877	0.286	90	98	91	41	-54	2.17E-6	8.94E-6	3.00E-5
OVCAR-5	0.527	1.467	1.371	1.411	1.338	0.715	0.046	90	94	86	20	-91	1.17E-6	5.03E-6	1.42E-5
OVCAR-8	0.403	1.457	1.515	1.528	1.536	0.765	0.148	105	107	107	34	-63	2.03E-6	7.49E-6	2.43E-5
NCI/ADR-RES	0.428	1.273	1.280	1.252	1.278	1.099	0.175	101	98	101	79	-59	5.43E-6	1.25E-5	2.86E-5
SK-OV-3	0.689	1.473	1.417	1.479	1.449	1.064	0.087	93	101	97	48	-87	3.00E-6	7.51E-6	1.76E-5
Renal Cancer															
786-0	0.443	1.813	1.755	1.803	1.737	0.706	0.104	96	99	94	19	-77	1.30E-6	5.28E-6	1.76E-5
A498	1.401	1.875	1.938	1.910	1.883	1.393	0.178	113	107	102	.	-87	1.07E-6	3.28E-6	1.24E-5
ACHN	0.423	1.617	1.516	1.612	1.544	1.098	0.033	92	100	94	56	-92	3.68E-6	7.99E-6	1.73E-5
CAKI-1	0.470	2.123	1.894	1.967	1.908	1.286	0.030	86	91	87	49	-94	3.20E-6	7.37E-6	1.65E-5
RXF 393	0.723	1.349	1.317	1.374	1.336	0.912	0.214	95	104	98	30	-70	1.70E-6	6.65E-6	2.09E-5
SN12C	0.570	1.726	1.713	1.791	1.741	1.024	0.087	99	106	101	39	-85	2.23E-6	6.90E-6	1.74E-5
TK-10	0.681	1.672	1.687	1.610	1.581	1.233	0.110	101	94	91	56	-84	3.66E-6	8.35E-6	1.90E-5
UO-31	0.517	1.694	1.601	1.592	1.542	1.005	0.092	92	91	87	41	-82	2.16E-6	7.20E-6	1.83E-5
Prostate Cancer															
PC-3	0.366	1.536	1.498	1.536	1.494	0.644	0.074	97	100	96	24	-80	1.45E-6	5.64E-6	1.71E-5
DU-145	0.330	1.311	1.319	1.359	1.314	0.746	0.045	101	105	100	42	-87	2.46E-6	7.10E-6	1.73E-5
Breast Cancer															
MCF7	0.402	2.309	2.117	2.178	2.018	0.414	0.223	90	93	85	1	-45	8.62E-7	3.44E-6	> 3.33E-5
MDA-MB-231/ATCC	0.623	1.590	1.619	1.624	1.587	0.593	0.101	103	104	100	-5	-84	9.94E-7	2.99E-6	1.24E-5
HS 578T	0.866	1.542	1.544	1.584	1.508	0.970	0.567	100	106	95	15	-35	1.22E-6	6.76E-6	> 3.33E-5
BT-549	0.788	1.785	1.698	1.740	1.730	1.065	0.124	91	96	95	28	-84	1.55E-6	5.89E-6	1.65E-5
T-47D	0.691	1.313	1.232	1.332	1.248	0.915	0.388	87	103	90	36	-44	1.83E-6	9.40E-6	> 3.33E-5
MDA-MB-468	0.748	1.767	1.734	1.778	1.611	0.502	0.250	97	101	85	-33	-67	6.57E-7	1.75E-6	1.07E-5

Figure S28. Tabular list of the antineoplastic activity exhibited by the drug compound **3** (**PT167**, **NSC 799315**) in the National Cancer Institute (NCI) Developmental Therapeutics Program (DTP) 60-cancer cell 5-dose testing with the Mean Optical Densities of the used vital stain sulforhodamine B (SRB) retained in the cells given. **Percent Growth**, **GI50** (**Growth Inhibition 50%**), **TGI** (**Total Growth Inhibition = 0% Growth**), and **LC50** (**Lethal Concentration 50% = –50% Growth**) are given in linear concentration units.

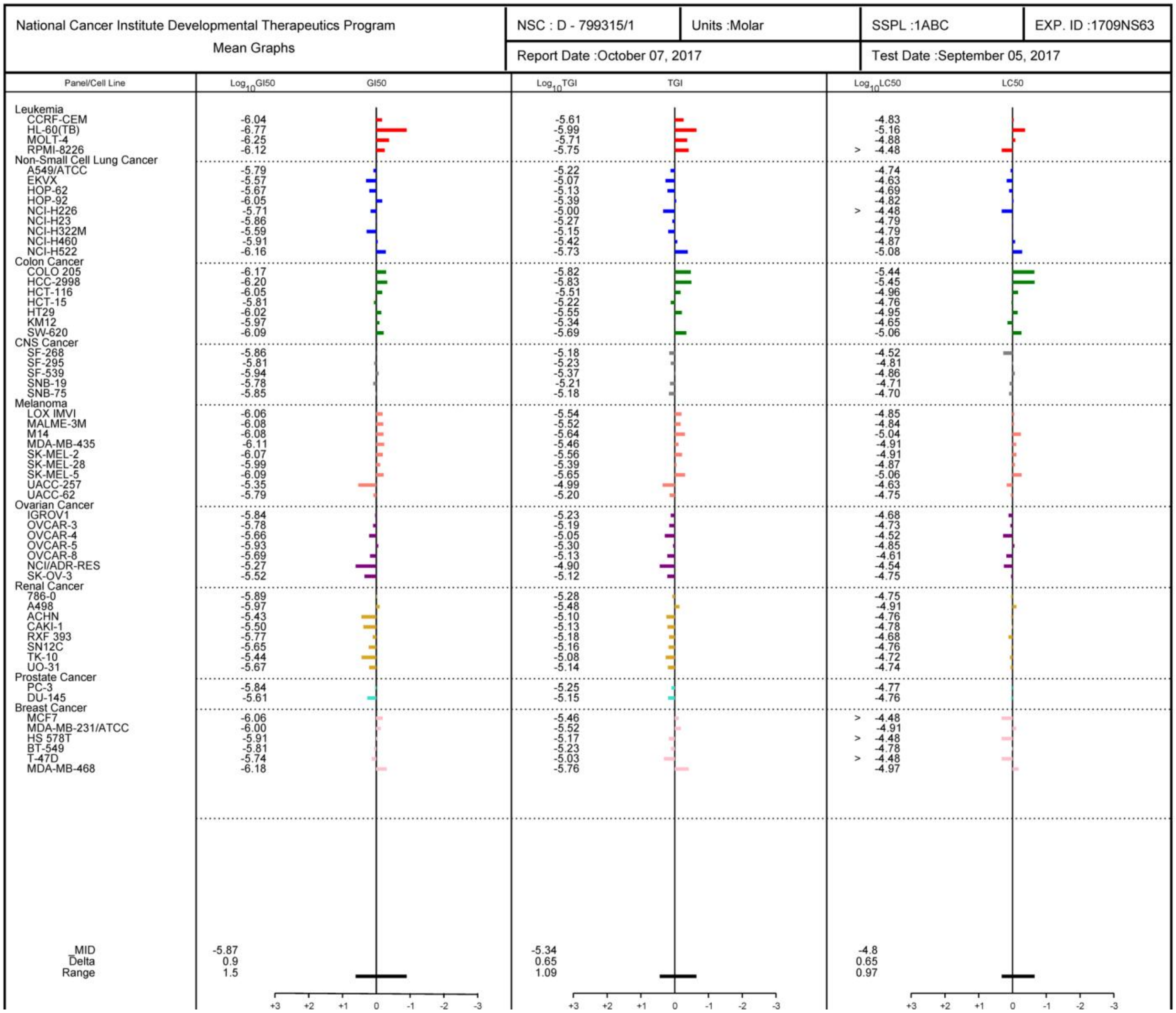

Figure S29. Graphic table presentation of the antineoplastic activity exhibited by the drug compound **3** (**PT167**, **NSC 799315**) in the National Cancer Institute (NCI) Developmental Therapeutics Program (DTP) 60-cancer cell 5-dose testing with the **Percent Growth**, **GI50** (**Growth Inhibition 50%**), **TGI** (**Total Growth Inhibition = 0% Growth**), and **LC50** (**Lethal Concentration 50% = –50% Growth**) given in linear concentration units. This is the summary of the results expressed in log_{10} units. The defined tumor inhibition criterion (**GI50**, **TGI**, or **LC50**) is recalculated in log_{10} expression, and the colored bars which indicate then the sensitivity of the individual tumor cell line to the agent are given, but in log_{10} units. **Bar to the left: less sensitive**

than mean; **Bar to the right: more sensitive than mean**. The most important feature is at the bottom of the page: the **MID** (**Mean of Inhibition Data**) indicates the mean concentration for all tested cell lines required for the drug to reach the defined tumor inhibition criterion (**GI50**, **TGI**, or **LC50**). It is given in **Log_{10}GI50**, **Log_{10}TGI** and **Log_{10}LC50**. From that values the corresponding **MID** is calculated. The more negative the **MID**, the more potent is the drug. The **MID** can be transformed from logarithmic into linear concentrations by the formula: $c = 10^{MID}$.
